# Bacterial Communities Associated with *Deschampsia antarctica* in Chronically Diesel-Contaminated Soils: Candidate Taxa for Microbe-Assisted Phytoremediation

**DOI:** 10.3390/microorganisms14091917

**Published:** 2026-08-31

**Authors:** Camila Tassano, Matías Javier Garavaglia, Marcos Leopoldo Esteso, Catalina Basile Dazzi, Juan Orlowski, Walter Patricio Mac Cormack, Jaco Vangronsveld, Sofie Thijs, Lucas Adolfo Mauro Ruberto, Francisco Massot

**Affiliations:** 1 Instituto Antártico Argentino, San Martín 1650, Buenos Aires, Argentina; camilatassano@gmail.com (C.T.);; 2 Laboratorio de Aplicaciones Biotecnológicas y Microbiología, Secretaría de Investigación, Universidad Nacional de Hurlingham, Hurlingham 1688, Buenos Aires, Argentina; matias.garavaglia@unahur.edu.ar; 3 Instituto de Nanobiotecnología, Facultad de Farmacia y Bioquímica, Universidad de Buenos Aires–CONICET, Ciudad Autónoma de Buenos Aires 1113, Buenos Aires, Argentina; 4 Consejo Nacional de Investigaciones Científicas y Técnicas, Buenos Aires 1425, Buenos Aires, Argentina; 5 Environmental Microbiology, Centre for Environmental Sciences, Department of Biology, Faculty of Sciences, Hasselt University, 3590 Diepenbeek, Belgium; 6 Department of Plant Physiology and Biophysics, Institute of Biological Sciences, Maria Curie-Skłodowska University, 20-031 Lublin, Poland

**Keywords:** *Deschampsia antarctica*, Antarctic soils, diesel contamination, phytoremediation, rhizospheric bacteria, endophytic bacteria, 16S rRNA amplicon sequencing, hydrocarbon-degrading bacteria, plant microbiome, PICRUSt2

## Abstract

More than six decades of human activity in Antarctica resulted in chronic diesel contamination of soils surrounding research stations. Phytoremediation assisted by the native vascular plant *Deschampsia antarctica* is one of the few remediation strategies compatible with the Antarctic Treaty System guidelines. Securing suitable root-associated microbial resources is central to this approach. This work characterized the bacterial communities of the rhizosphere and root endosphere of *D. antarctica* growing in a chronically diesel-contaminated soil at Carlini Station and in three pristine sites on 25 de Mayo (King George) Island, South Shetland Islands, combining culture-independent 16S rRNA gene amplicon sequencing, PICRUSt2 functional prediction and culture-dependent bacterial strain isolation. The rhizospheric microbial community was consistently more diverse than those inhabiting the endosphere and was more strongly structured by site and associated soil physicochemical variables, with the contaminated site showing the most distinct composition, whereas the endosphere remained comparatively stable and host-selected. The community associated with the chronically contaminated site showed pronounced compositional differences and higher predicted abundances of hydrocarbon-degradation pathways in the rhizosphere. *Polaromonas*, *Rhodococcus*, *Mycobacterium* and *Devosia* were repeatedly associated with the community from the contaminated site across taxonomic, biomarker and predicted functional analyses. Members of the genera *Polaromonas*, *Rhodococcus* and *Devosia* were also recovered in culture. These taxa represent suitable candidates for the future development of microbe-assisted phytoremediation strategies in Antarctica.

## 1. Introduction

More than six decades of scientific and logistical activity in Antarctica have left considerable environmental liabilities, particularly in ice-free coastal areas where most research stations are located. Even today, petroleum hydrocarbons (as Diesel Antarctic Blends mainly) remain the primary energy source in most of the Antarctic stations [1,2]. As a result, terrestrial diesel spills are a recurrent reality: transport and routine fuel use for power generation, together with transfer mishandling and the weathering of storage tanks, has chronically contaminated soils in the vicinity of the Antarctic stations [3]. Implementation of clean-up activities in these sites is difficult due to the isolation of the stations, logistic costs, extreme weather conditions and environmental protocols of the Antarctic governance system [4]. This gives rise to the need to apply environmentally friendly remediation methods accepted by the international Antarctic community. In this regard, bioremediation constitutes a suitable biotechnological tool for the clean-up of hydrocarbons from Antarctic soils [5].

Within this constrained scenario, phytoremediation emerges as a promising and protocol-compliant strategy [6,7,8]. Moreover, microorganism-assisted phytoremediation using hydrocarbonoclastic plant-growth promoting bacteria has shown promise to stimulate the degradation of hydrocarbons and in the rhizosphere to promote plant growth and nutrient acquisition in these harsh conditions [9,10]. It involves an increase in the rate of xenobiotic degradation by increasing the numbers and catabolic activity of associated bacteria within the root zone of plants [11]. This process exploits the synergistic interactions between plants and microorganisms to mitigate or even completely eliminate the harmful effects of contaminants in the environment. The presence of plants can alleviate problems that are common in soils of extremely cold climates: the increased viscosity of hydrocarbons, with the consequent decrease in their water solubility, impairs the natural attenuation of hydrophobic contaminants by lowering their bioavailability. Furthermore, the continuous freezing and thawing cycle of water promotes hydrocarbon mobility, altering the original distribution of the contamination. Due to the guidelines from The Antarctic Treaty and its Environment Protection Protocol, the introduction of allochthonous organisms is forbidden. This restricts the phytoremediation approach to only two native vascular plants, *Deschampsia antarctica* É. Desv. (Poaceae; Antarctic grass) and *Colobanthus quitensis* (Caryophyllaceae, Antarctic pearlwort). The potential of *D. antarctica* has been investigated by Basile Dazzi et al. [12], demonstrating that in field conditions, individuals can tolerate diesel in soil up to 40,000 mg kg^−1^ without experiencing a 50% reduction in biomass, root growth, or chlorophyll content.

Securing this unique psychrophilic microbial resource is central to the strategy, since the plants alone seldom achieve complete hydrocarbon removal. Root-associated bacteria capable of degrading hydrocarbons while promoting plant growth can improve both plant performance under contaminant stress and degradation of the hydrocarbons [13]. Microbiomes can be studied and harnessed through two complementary paradigms: a bottom-up and a top-down approach [14]. The bottom-up approach is culture-dependent: microorganisms are isolated, identified, and characterized on the basis of one or more desired traits to later be combined into defined inocula or synthetic communities (SynComs) [15,16]. This route provides a good control over composition and function, but it carries a well-documented weakness: the in vitro performance of an isolate does not necessarily predict its behaviour, ecological role, or persistence once it is reintroduced into the plant, and the competitive fitness and ecological stability of bottom-up assemblies are frequently poor in native soil [16]. As a result, strain selection based on isolation only often leads to laborious screening and unsuccessful application in the field. The top-down approach is culture-independent, and uses high-throughput methods to characterize the microbiome around the roots [17], identifying the set of microorganisms that are ecologically relevant for the desired functionalities. These microorganisms can be stimulated directly or indirectly and their genetic potential harnessed to optimize functions at the individual and ecosystem levels, like promoting hydrocarbon catabolism and alleviating plant stress. Neither paradigm is sufficient on its own, and current consensus favours their integration [18]. In practice, this can be operationalised as sequencing-guided cultivation, in which amplicon profiles direct whom to target and under which conditions to recover strains, turning blind isolation into a focused effort [19].

A profound understanding of the ecology of the *D. antarctica* root microbiome under chronic hydrocarbon contamination is required for the development of a suitable and effective phytoremediation approach based on robust information. In this study, the bacterial communities associated with *D. antarctica* growing in a chronically diesel-contaminated soil at Carlini Station and in three pristine sites on ASPA 132, Potter peninsula, 25 de Mayo (King George) Island were characterized using both culture-dependent and independent-methods. The following scientific questions guided the experimental design of this work: (i) does the root associated microbiome of the contaminated soil differ from those of pristine microbiomes? (ii) do the rhizosphere and endosphere harbor different and complementary microbial assemblages? Is it equivalent to prospect for phytoremediation-useful microorganisms in the rhizosphere versus the endosphere? (iii) is it possible to identify cultivable, hydrocarbonoclastic strains to begin designing an effective and robust phytoremediation tool? By answering these questions, this work aims to provide the first concrete steps toward an integrated, microbe-assisted phytoremediation strategy in the Antarctic continent.

## 2. Materials and Methods

### 2.1. Study Sites and Sample Collection

**Figure 1:**
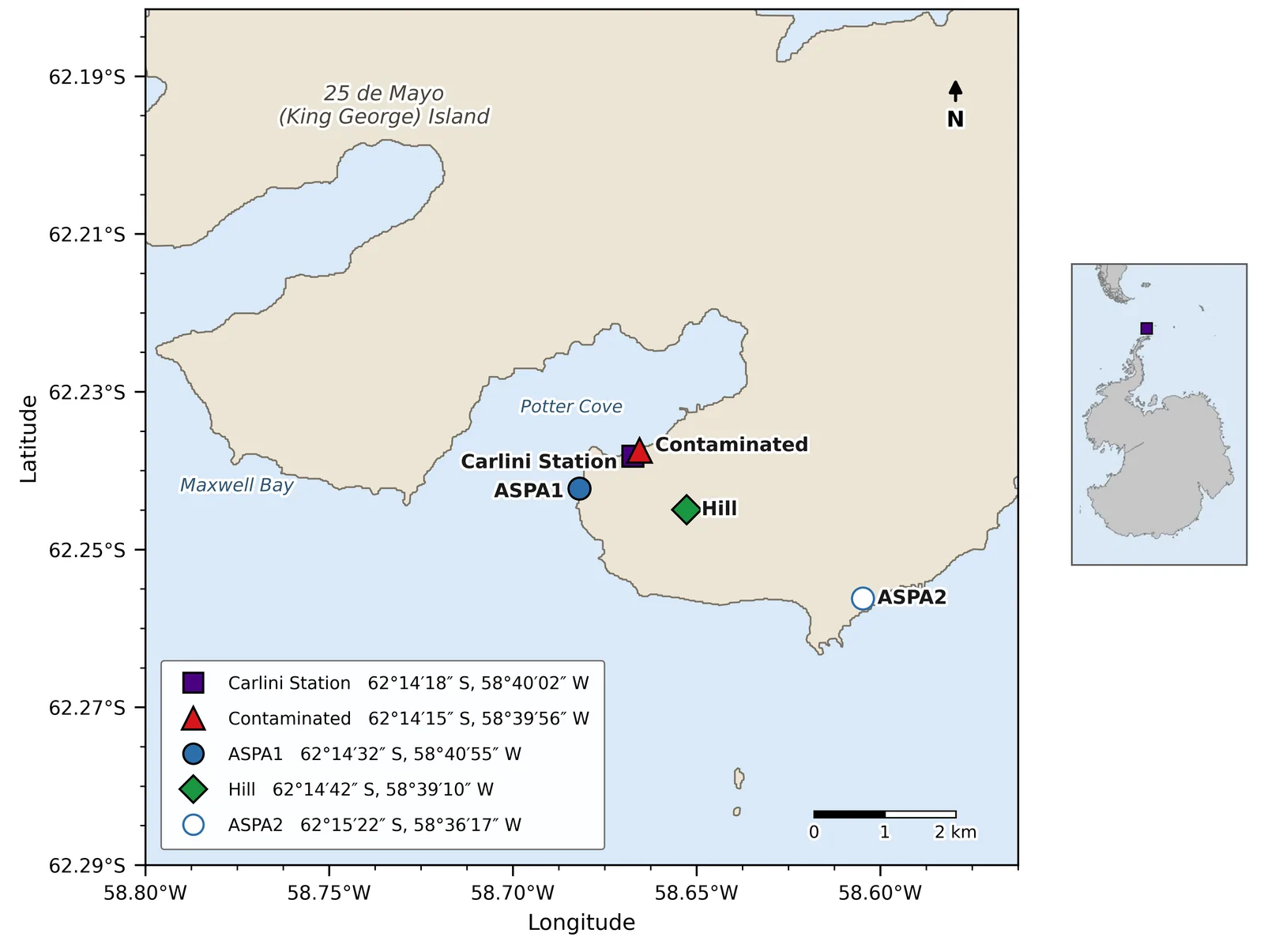
Map of sampling sites near Carlini Station, 25 de Mayo (King George) Island, South Shetland Islands, Antarctica. Three *D. antarctica* individuals were sampled at each pristine site and six at the contaminated site (Supplementary Table S2).

Sampling was conducted in the vicinity of Carlini Station, 25 de Mayo (King George) Island, South Shetland Islands, Antarctica (Figure 1). Four sites representing contrasting environmental conditions were selected: (1) ASPA1 (62°15′22.3″ S, 58°36′17.2″ W), an Antarctic Specially Protected Area (ASPA) characterized by low organic matter content and sandy soils; (2) ASPA2 (62°14′32.2″ S, 58°40′54.8″ W), a second ASPA with the highest organic carbon, organic matter and phosphorus contents among the sites analyzed (both protected areas have restricted human access and limited anthropogenic disturbance); (3) Hill (62°14′41.8″ S, 58°39′09.6″ W), a non-contaminated site located outside protected areas and characterized by lower sand content and a sandy loam texture; and (4) a contaminated site (62°14′14.8″ S, 58°39′55.7″ W) located adjacent to the station power generation area and chronically exposed to Antarctic Blend Diesel (ABD) and lubricants for more than three decades. Detailed soil physicochemical characteristics and total petroleum hydrocarbon concentrations are provided in Supplementary Table S1.

This study is based on two independent field campaigns. During the 2019–2020 Antarctic summer campaign, 15 *D. antarctica* individuals were collected at the four sites, yielding 15 rhizosphere and 15 root endosphere samples, resulting in a total of 30 samples. The sampling design included three rhizosphere and three endosphere samples from each pristine site (ASPA1, ASPA2 and Hill), and six rhizosphere and six endosphere samples from the contaminated site. The number and distribution of samples across sites and plant compartments are summarized in Supplementary Table S2. During the 2020–2021 Antarctic summer campaign, a second and independent sampling was performed at the contaminated site for the culture-dependent isolation of rhizosphere bacteria (Section 2.7).

In both campaigns, plants were selected according to uniform criteria in order to minimize phenology as a source of variation. All sampled *D. antarctica* individuals were well-established, mature tussock-forming plants of comparable developmental stage, bearing inflorescences at the time of collection (January, austral summer; reproductive phase). Plant size ranged from ~10 to 20 cm across sites, a variation likely reflecting differences in soil conditions rather than developmental stage. Seedlings and senescent individuals were excluded to minimize plant age and phenology as sources of variation in the root-associated microbial communities.

Rhizospheric soil was defined as the soil that remained attached to the root after gentle agitation. This fraction was recovered by washing the root systems in a sterile extraction solution containing 0.9% (*w*/*v*) NaCl and 0.01% (*v*/*v*) Tween 80, and the resulting suspension was centrifuged. Root endosphere samples consisted of root tissue after three successive washes with the same extraction solution to remove all soil attached to the root. Samples were immediately stored at −80 °C until DNA extraction. Compartment definitions and the washing procedure followed methods established for root microbiome studies [20]. In the 2020–2021 campaign, only the rhizosphere was sampled, following the same criterion and using the same extraction solution (Section 2.7).

### 2.2. DNA Extraction

Rhizosphere DNA was extracted using the DNeasy PowerSoil^®^ Kit (QIAGEN, Hilden, Germany). Five hundred milligrams of the rhizosphere pellet recovered as described in Section 2.1 was used for DNA extraction. Before extraction, samples were treated with lysozyme according to the manufacturer’s instructions and incubated at 37 °C. Three freeze–thaw cycles (−70 °C/65 °C) were subsequently applied before continuing with the standard extraction protocol. Endosphere DNA was extracted using the DNeasy PowerPlant^®^ Pro Kit (QIAGEN, Hilden, Germany). Washed roots (Section 2.1) were cut into small fragments and homogenized, and DNA extraction was performed using 50 mg of plant tissue following the manufacturer’s protocol.

### 2.3. Amplicon Library Preparation and Sequencing

The V3–V4 region of the bacterial 16S rRNA gene was amplified using primers 341F (CCTACGGGNGGCWGCAG) and 805R (GACTACHVGGGTATCTAATCC). Amplicon libraries were prepared in-house using the Nextera XT library preparation protocol and indexed with the Nextera XT Index Kit (Illumina, San Diego, CA, USA). Sequencing was performed on an Illumina MiSeq platform using paired-end 2 × 301 bp chemistry. Demultiplexed paired-end reads were used for all downstream analyses. The 16S rRNA gene amplicon sequencing data generated in this study have been deposited in the NCBI Sequence Read Archive SRA under BioProject accession number PRJNA1483961.

### 2.4. Bioinformatics and Statistical Analysis

Raw paired-end 16S rRNA gene sequences were processed in R v4.4.1 [21] using DADA2 v1.30.0 [22]. Reads were truncated at 260 bp (forward) and 240 bp (reverse) based on quality score distributions. Reads containing ambiguous bases were removed during quality filtering. Amplicon sequence variants (ASVs) were inferred following the standard DADA2 workflow, including dereplication, error-rate learning, pooled sample inference, paired-end read merging and chimera removal. Paired reads were merged using a minimum overlap of 12 bp, and only sequences within the expected size range of the V3–V4 region of the 16S rRNA gene (402–428 bp) were retained. Chimeric sequences were identified and removed using the consensus method. Taxonomic assignment was performed against the SILVA v138.2 reference database [23]. Sequences classified as chloroplasts, mitochondria or non-bacterial taxa were removed before downstream analyses. The resulting ASV table was used for all subsequent diversity, taxonomic and functional analyses. Prior to alpha-diversity analyses, and to account for differences in sequencing depth among samples, the ASV table was rarefied to 8678 sequences per sample, corresponding to the lowest sequencing depth obtained, and all samples were retained after rarefaction. Rarefaction curves reached a plateau at this depth, indicating that the sequencing effort was sufficient to capture the diversity of the bacterial communities analyzed. Alpha diversity was assessed using the Shannon diversity index and the number of observed ASVs. Differences among sampling sites were evaluated separately for endosphere and rhizosphere communities using Kruskal–Wallis tests. When significant differences were detected, pairwise comparisons were performed using Dunn’s test with Bonferroni correction. Statistical analyses were conducted in R v4.4.1.

Beta diversity was evaluated using Principal Coordinates Analysis (PCoA) based on Bray–Curtis dissimilarities calculated from relative abundance data. Analyses were conducted separately for endosphere and rhizosphere communities to evaluate specific patterns within each plant compartment. Differences in community composition among sites were assessed using permutational multivariate analysis of variance (PERMANOVA; adonis2) with site as a four-level factor. The relationship between microbial community structure and soil physicochemical variables (TOC, OM, clay, silt, sand and phosphorus) was assessed by fitting these variables post hoc onto the ordination with the envfit function; both analyses were performed with the vegan package [24] in R v4.4.1 using 999 permutations. Only variables showing significant associations (*p* ≤ 0.05) were displayed in the ordination plots. Envfit provides a descriptive, correlative measure of the association between each variable and the ordination and does not partition variance or test causal effects. Total petroleum hydrocarbon (TPH) concentration was not included as an explanatory variable because it was below the analytical limit of detection at all three pristine sites (Supplementary Table S1) and therefore acted as a binary indicator of the contaminated site rather than as an independent gradient. Homogeneity of multivariate dispersion was assessed using the betadisper test [25]. Differentially abundant taxa were identified using Linear Discriminant Analysis Effect Size (LEfSe) implemented in the microbiomeMarker package [26]. Prior to analysis, count data were normalized using counts per million (CPM). Samples were grouped according to plant compartment (endosphere or rhizosphere) and, within each compartment, into two groups: samples from the chronically contaminated site and samples from the three pristine sites combined. Because only one contaminated location was available, this contrast compares a single site against three pooled sites and therefore does not constitute a replicated test of a contamination effect; LEfSe was used here as an exploratory biomarker-discovery tool to identify candidate taxa associated with the contaminated site. Taxa were considered significant biomarkers when they exhibited an LDA score ≥ 3.0 (log10 scale) and a significance threshold of α = 0.01 based on the Wilcoxon rank-sum test. Functional prediction analyses were performed using the PICRUSt2 full pipeline [27] to infer the functional potential of the core microbiome. Core ASVs were defined as those present in at least 50% of samples within each site–compartment group and with a mean relative abundance ≥ 0.1%. The complete list of core ASVs and their taxonomic assignments is provided in Supplementary Table S3. Weighted Nearest Sequenced Taxon Index (NSTI) values were generated for each sample to assess prediction accuracy. Lower NSTI values indicate closer phylogenetic relatedness to reference genomes and, therefore, greater confidence in the inferred functional profiles [27] (Supplementary Table S4). A curated subset of 669 KEGG Orthologs (KOs) associated with hydrocarbon degradation, nutrient cycling, siderophore production and other plant growth-promoting traits was selected for downstream analyses (Supplementary Table S5). Pathway abundances were estimated by summing the relative abundances of KOs assigned to each pathway and calculating mean values. Values were log10-transformed prior to visualization.

For each KO, differences among sites were evaluated using Kruskal–Wallis tests, followed by Dunn’s post hoc pairwise comparisons against the contaminated site. *p* values were adjusted using the Benjamini–Hochberg method. KOs were considered significant when adjusted *p* values were below 0.05. Among these, the KOs discussed here were selected according to their ecological relevance and the degradation pathways with which they were associated. To identify the microbial groups predicted to contribute to the selected functions, stratified PICRUSt2 outputs were used. For each selected KO, functional contributions were summarized at the genus level. Genera lacking genus assignment were grouped as unclassified. For visualization, the ten main contributing genera were retained for each KO and minor contributors were grouped as “Other”. Mean contributions at the genus level were calculated for each site and displayed as stacked bar plots.

### 2.5. Soil Physicochemical Characterization

Soil physicochemical analyses were performed at the Soil Science Department of the Faculty of Agronomy, University of Buenos Aires (FAUBA). Total organic carbon (TOC) was determined using the modified Walkley–Black method [28], and organic matter (OM) was estimated from the resulting oxidizable organic carbon values. Available phosphorus (*p*) was determined using the Bray and Kurtz method [29] according to IRAM–SAGyP 29570-1:2010, and soil texture was determined using the Bouyoucos hydrometer method [30]. Sand, silt and clay percentages were used to assign soil textural classes.

### 2.6. Hydrocarbon Quantification by Infrared Spectroscopy

For hydrocarbon quantification, soil samples (5 g) were extracted with 20 mL of tetrachloroethylene (IR quality, Sintorgan, Villa Martelli, Buenos Aires, Argentina) in 40 mL Teflon tubes (Nalgene, Rochester, NY, USA). A spoon-tip of sodium sulfate was added to each tube in order to remove any remaining aqueous fraction in the organic phase. After that, 0.5 g of Silica Gel 60 (0.063–0.200 mm, Merck, Darmstadt, Germany) was added to each tube to remove polar compounds from the organic phase. Flasks were shaken (200 rpm) for 3 h at 25 °C. Afterwards, samples were centrifuged (15 min, 7600 *g*), filtered to avoid the presence of soil particles and transferred to a quartz cell for analysis. Five replicates were extracted and measured at each sampling time. The results were expressed as the average of those five determinations. Hydrocarbon quantification was performed at Carlini’s scientific station laboratory using a Fourier Transform Infrared spectrophotometer (IR Affinity 1, Shimadzu, Corp., Kyoto, Japan), employing a modification of ASTM D7066 method. Calibration was performed with diesel fuel standards prepared in HPLC-grade tetrachloroethylene, and peak areas in the range of 2850 to 3000 cm^−1^ were considered. The Infrared spectra determination was set for 45 scans per sample and the resolution was set at 2.0. Samples were measured according to the same protocol, and final values were obtained by extrapolating each sample’s peak area in the respective calibration curve.

### 2.7. Bacterial Strain Collection: Isolation and Identification

Bacterial isolation was performed on *D. antarctica* individuals collected at the contaminated site (62°14′14.8″ S, 58°39′55.7″ W) during the 2020–2021 Antarctic summer campaign. Only the rhizosphere compartment was recovered, as defined in Section 2.1. Approximately 4 g of root tissue was washed with the extraction solution (0.9% *w*/*v* NaCl, 0.01% *v*/*v* Tween 80) to recover the rhizosphere soil. The resulting washing suspensions were plated onto R2A agar supplemented with root extract and cycloheximide (100 mg L^−1^) to suppress fungal growth. To prepare the root extract, 1 g of washed roots was homogenized with glass beads in 2 mL microcentrifuge tubes at 2000 rpm for 2 min. The resulting homogenate was added to 0.5 L of R2A medium before sterilization, corresponding to a final root concentration of 2 g L^−1^. Isolates were identified by full-length 16S rRNA gene sequencing using a MinION device (Oxford Nanopore Technologies, Oxford, UK). The approximately 1500 bp 16S rRNA gene was amplified using the universal primers Bac_27f and Bac_1492r carrying Oxford Nanopore Technologies tail sequences. Libraries were prepared according to the ONT Ligation Sequencing V14 amplicon protocol using the SQK-LSK114 and EXP-PBC096 kits for multiplexed sequencing on R10.4.1 flow cells (FLO-MIN114). Real-time basecalling and demultiplexing were performed using MinKNOW, and taxonomic classification was conducted with the EPI2ME bacterial 16S workflow.

## 3. Results and Discussion

### 3.1. Alpha and Beta Diversity

**Figure 2:**
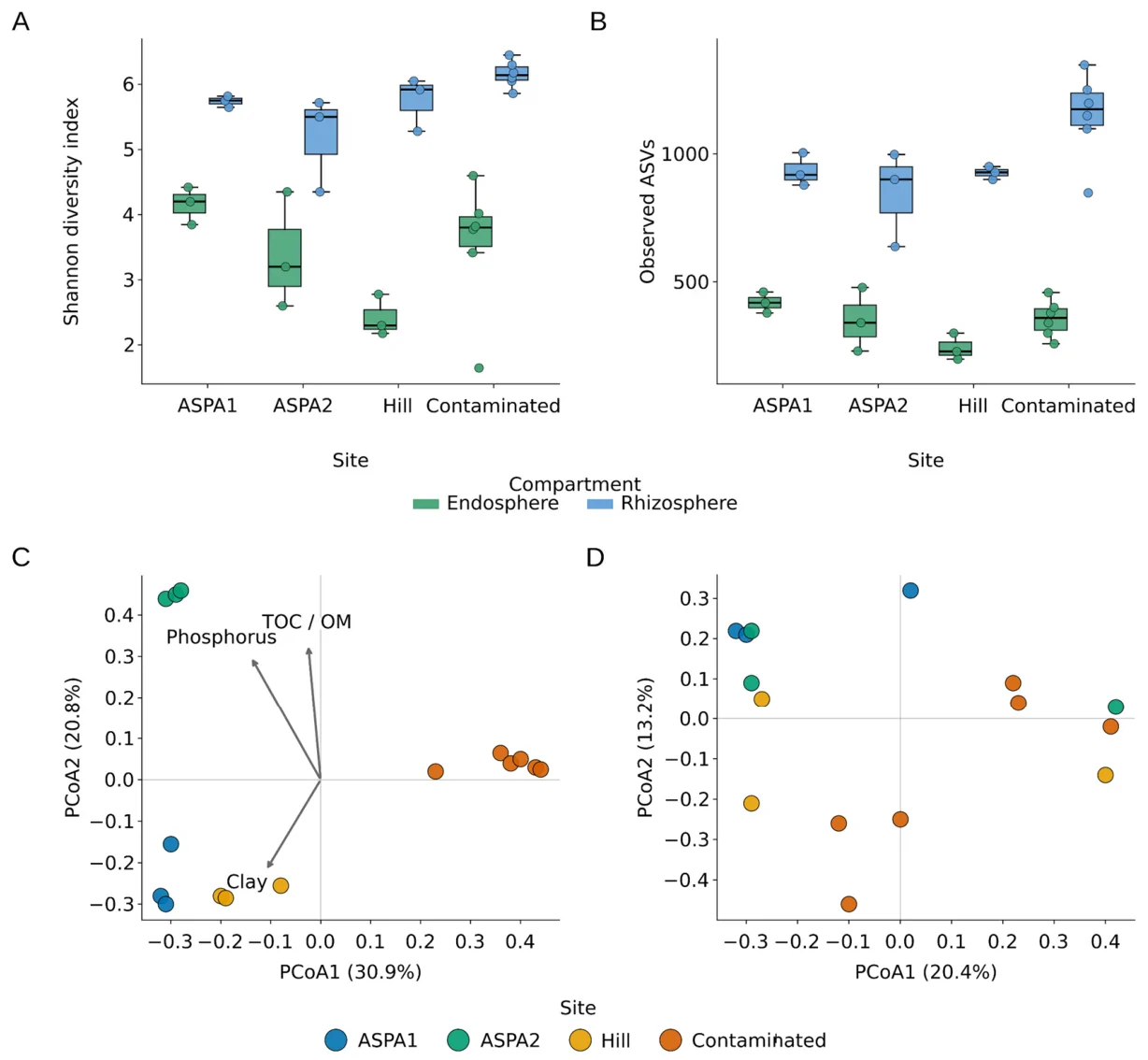
Alpha and beta diversity of bacterial communities associated with *Deschampsia antarctica* across sampling sites. (**A**,**B**) Shannon diversity index and observed ASVs in endosphere and rhizosphere communities from ASPA1, ASPA2, Hill, and contaminated sites (*n* = 3 plants per pristine site and *n* = 6 at the contaminated site, sampled in both compartments; 30 samples in total; Supplementary Table S2). Boxes represent the interquartile range, horizontal lines indicate the median, and points correspond to individual samples. Differences among sites within each compartment were evaluated using Kruskal–Wallis tests. Shannon diversity differed significantly among rhizosphere sites at the global level (H = 8.13, *p* = 0.043), although no pairwise comparison remained significant after post hoc correction. No significant differences were detected for rhizosphere richness or for endosphere Shannon diversity and richness. (**C**) Principal Coordinates Analysis (PCoA) of rhizosphere communities based on Bray–Curtis dissimilarities. (**D**) Principal Coordinates Analysis (PCoA) of endosphere communities based on Bray–Curtis dissimilarities. Differences in community composition among sites were assessed by PERMANOVA (rhizosphere: R^2^ = 0.645, *p* = 0.001; endosphere: R^2^ = 0.244, *p* = 0.139). Significant soil physicochemical variables were fitted onto the ordination using envfit; arrows indicate correlative associations and do not imply causal effects.

A total of 8316 ASVs were obtained after data filtering. Across all sites, the rhizosphere consistently showed higher diversity than the endosphere (Figure 2A,B). While endosphere microbiomes showed relatively homogeneous richness (observed ASVs: H = 5.56, *p* = 0.135) and Shannon diversity (H = 5.20, *p* = 0.158), the rhizosphere displayed significant differences in Shannon diversity (H = 8.13, *p* = 0.043), although no significant differences in richness (H = 6.73, *p* = 0.081). No pairwise comparison remained significant after post hoc correction, indicating that the global difference was not attributable to any single contrast between sites. Shannon diversity in the rhizosphere ranged from 5.21 at ASPA2 to 6.19 at the contaminated site, whereas endosphere values ranged from 2.36 at Hill to 4.24 at ASPA1, with Hill also showing the lowest endosphere richness (660 ASVs). ASV richness followed a similar pattern, with the contaminated site at the upper end of the rhizosphere range. The contaminated site therefore showed the highest values in the rhizosphere but not in the endosphere, and no pairwise difference was statistically supported. The higher diversity and richness observed in the rhizosphere compared to the endosphere have been widely reported across plant–microorganism systems, where microbial communities become progressively filtered from the rhizosphere to the endosphere [31,32,33]. This behavior is consistent with the plant microbiome assembly model proposed by Bulgarelli et al. [34] in which microorganisms are initially recruited into the rhizosphere and subsequently filtered as they enter the root interior. In the case of *D. antarctica*, similar trends have been described, with rhizosphere communities typically showing higher diversity (Shannon ~6) and richness (~1500 OTUs), while the endosphere exhibits lower values (Shannon ~3–4; <500 OTUs) [35,36,37,38], consistent with the patterns observed in this study.

Principal Coordinates Analysis (PCoA) revealed that rhizosphere samples clustered according to their site of origin, showing a clear separation between sites (Figure 2C). PERMANOVA analysis indicated that 64.5% of the variation could be explained by site (R^2^ = 0.645; F = 6.66; *p* = 0.001). Betadisper did not indicate significant differences in dispersion between sites (permutest, *p* = 0.477), supporting the interpretation that the observed differences reflect shifts in community composition rather than differences within group variability. Of the six soil variables fitted post hoc onto the ordination (envfit), phosphorus (r^2^ = 0.983; *p* = 0.001), total organic carbon (TOC; r^2^ = 0.942; *p* = 0.001), organic matter (OM; r^2^ = 0.942; *p* = 0.001) and clay (r^2^ = 0.596; *p* = 0.006) were significantly associated with rhizosphere community composition, whereas sand (r^2^ = 0.344; *p* = 0.090) and silt (r^2^ = 0.297; *p* = 0.129) were not. In contrast, the PCoA ordination revealed only limited site-related variation in endosphere community composition, with samples showing partial clustering and a relatively homogeneous microbial assemblage across sites (Figure 2D). PERMANOVA analysis indicated that site explained only 24.4% of the total observed variation in microbial community composition (R^2^ = 0.244; F = 1.19; *p* = 0.139). The homogeneity of the dispersion (Betadisper) did not detect significant differences (permutest, *p* = 0.400), supporting these results. Overall, these findings indicate that the site had a limited and nonsignificant effect on endosphere community structure in the sampled *D. antarctica* individuals. Regarding edaphic variables, none of the evaluated soil properties showed significant associations with endosphere bacterial community composition (*p* > 0.05). Although silt showed the highest explanatory value (r^2^ = 0.272; *p* = 0.157), it did not reach statistical significance. Likewise, sand (r^2^ = 0.147; *p* = 0.380), phosphorus (r^2^ = 0.081; *p* = 0.622), clay (r^2^ = 0.022; *p* = 0.887), total organic carbon (TOC; r^2^ = 0.004; *p* = 0.978), and organic matter (OM; r^2^ = 0.004; *p* = 0.978) were not significantly correlated with endosphere community composition.

Previous studies on Antarctic vascular plants have identified soil physicochemical characteristics and microbiome compartmentalization as major determinants of microbial community structure [35,37]. Rhizosphere communities in the present study showed pronounced spatial variation, whereas endosphere communities exhibited comparatively greater stability across sites. As the rhizosphere is the interface between plant roots and soil, it is highly responsive to edaphic conditions and compounds released by plant roots, which can promote the enrichment of microorganisms through the rhizosphere effect [39,40,41].

Regarding patterns associated with the contaminated site, alpha diversity was neither increased nor reduced in either compartment (Figure 2A,B). Gillan et al. [42] proposed that alpha diversity is a limited indicator of environmental stress, particularly under chronic contamination, where communities with similar diversity levels may differ substantially in composition, as sensitive taxa are replaced by tolerant ones. Our study system meets this condition of chronic exposure, as the contaminated site has received Antarctic Blend Diesel and lubricants for more than three decades. The concentration of total petroleum hydrocarbons at the contaminated site was 900 ± 50 mg kg^−1^, whereas TPH concentrations at ASPA1, ASPA2 and Hill were below the analytical limit of detection (Supplementary Table S1). Consistent with this scenario, the contaminated site was characterized by a shift in community composition rather than by a loss of diversity. Rhizosphere communities were compositionally distinct at all four sites (Figure 2C), but whereas the pristine sites differed from one another mainly along the second ordination axis, the contaminated site was separated from all of them along the first. In the same sense, contaminants may alter microbial functional potential without necessarily affecting alpha diversity [43]. Under hydrocarbon exposure, changes in root exudation may favor microbial taxa capable of exploiting compounds released by plant roots while tolerating contamination stress [44,45]. The greater representation of such taxa may account for the compositional turnover described above, particularly in oligotrophic soils where the contaminant can itself act as a carbon substrate [46,47]. These patterns are also compatible with the known plasticity of rhizosphere communities, which can adjust their composition and functional potential in response to environmental disturbances and may support the establishment of specialized microbiomes with enhanced hydrocarbon-degradation capacity near plant roots [48,49,50,51]. Accordingly, the pronounced compositional differences observed at the contaminated site suggest a greater representation of microorganisms potentially adapted to pollutant stress. Given the history of diesel input at the contaminated site and the TPH concentrations detected there, in contrast to the undetectable levels at the three pristine sites, the pattern is ecologically consistent with hydrocarbon exposure, although its contribution cannot be separated from other site-specific characteristics.

Endophytic communities appeared to be more strongly shaped by host selection than by environmental conditions. This is consistent with a previous study that showed that host species explained a substantially larger proportion of variation in endosphere composition than in rhizosphere communities [32]. Even under hydrocarbon stress, the host plant remains a major factor structuring endophytic bacterial communities [9]. Due to their intimate association with plant tissues, endophytic microorganisms are frequently linked to plant fitness and stress tolerance mechanisms, including phytohormone production and ACC deaminase activity [52,53]. These contrasting patterns highlight the complementary roles of both compartments. While the rhizosphere, being strongly structured by environmental factors, represents a reservoir of diverse and potentially specialized hydrocarbon-degrading microorganisms, the endosphere, shaped by host-mediated selection, may harbor functionally relevant taxa associated with plant fitness and stress tolerance. Therefore, both compartments constitute valuable targets for the isolation of microorganisms with potential applications in phytoremediation.

### 3.2. Taxonomic Composition of Bacterial Communities

**Figure 3:**
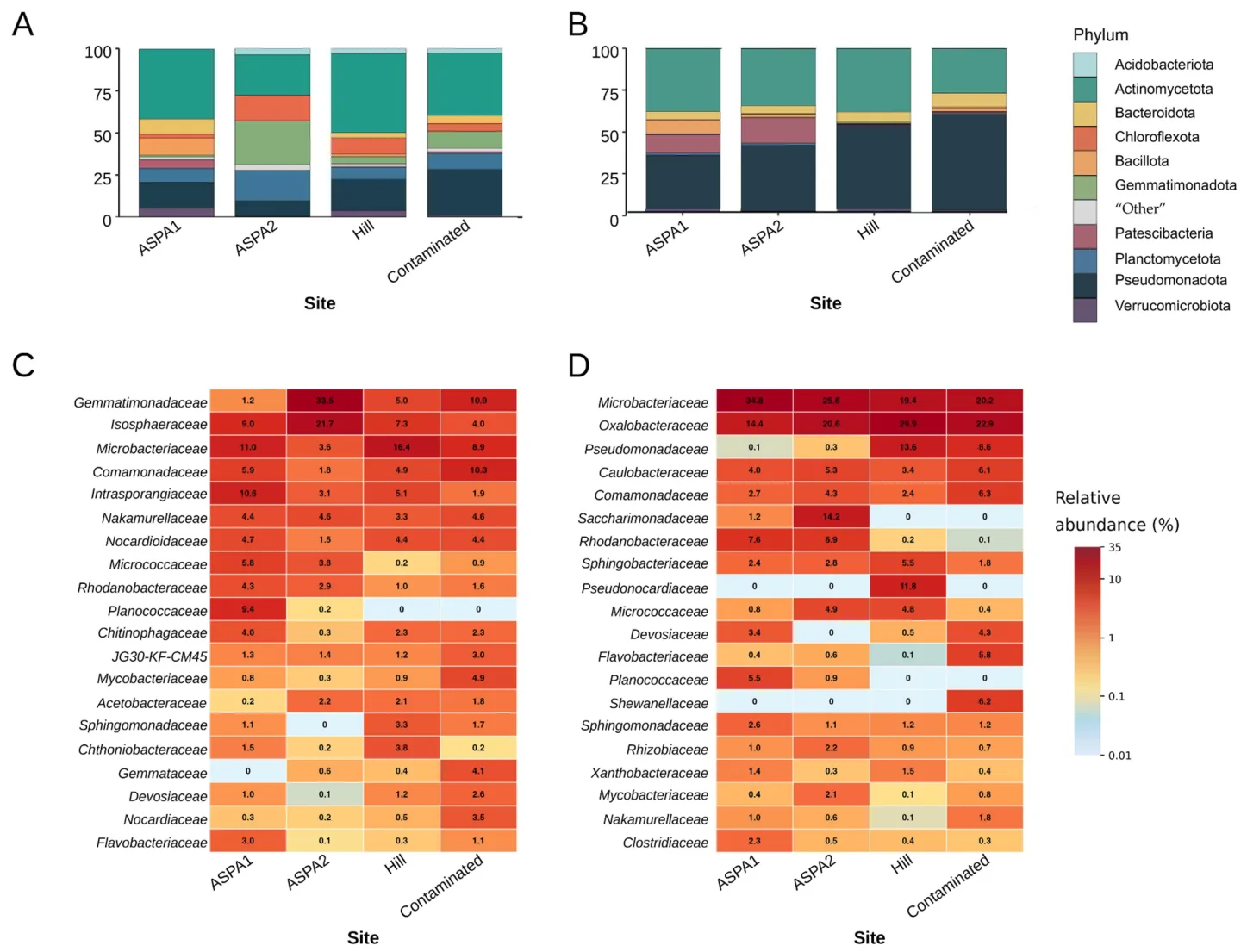
Taxonomic composition of bacterial communities associated with *Deschampsia antarctica* across sampling sites. (**A**) Mean relative abundance of the dominant bacterial phyla in rhizosphere communities from ASPA1, ASPA2, Hill and contaminated sites. (**B**) Mean relative abundance of the dominant bacterial phyla in endosphere communities from ASPA1, ASPA2, Hill and contaminated sites. (**C**) Heatmap showing the relative abundance (%) of the 20 most abundant bacterial families in rhizosphere communities. (**D**) Heatmap showing the relative abundance (%) of the 20 most abundant bacterial families in endosphere communities.

At the phylum level (Figure 3A,B), rhizosphere and endosphere communities exhibited broadly similar taxonomic profiles, being mainly dominated by Actinomycetota and Pseudomonadota across all sites. Actinomycetota represented, on average, 37.1% of rhizosphere communities and 32.6% of endosphere communities, whereas Pseudomonadota accounted for 19.4% and 50.3%, respectively. These phyla have likewise been identified as the dominant bacterial groups in previous studies of *D. antarctica* microbiomes [38,54]. Despite the shared dominance of these two phyla, clear compartment-specific differences were observed.

Rhizosphere communities showed a broader representation of several phyla, including Gemmatimonadota (10.1%), Planctomycetota (10.4%), Chloroflexota (7.2%) and Bacillota (2.4%, on average). In contrast, endosphere communities displayed lower diversity and a stronger dominance of Pseudomonadota (50.3%). Comparable patterns have been reported in other studies of microbiomes associated with Antarctic plants [36,55,56,57]. The comparison among sites revealed differences in phylum-level composition in both plant compartments, most notably a higher relative abundance of Pseudomonadota at the contaminated site. At the contaminated site, Pseudomonadota accounted for 26.9% of the rhizosphere community and 59.9% of the endosphere community. This trend was most pronounced in endosphere communities, where this phylum became increasingly dominant, whereas rhizosphere communities retained substantial contributions from Actinomycetota (36.8%) and other less abundant phyla, preserving a broader taxonomic structure despite contamination. The differences observed at the phylum level were further resolved when communities were examined at the family level. Multiple rhizosphere families identified in the present study (Figure 3C), including Chitinophagaceae, Comamonadaceae and Sphingomonadaceae, have also been reported in *D. antarctica* rhizosphere communities [36,55,57]. At this taxonomic level, patterns associated with contamination became more apparent. Rhizosphere samples from the contaminated site exhibited higher relative abundances of Comamonadaceae (10.3%), Mycobacteriaceae (4.9%), Gemmataceae (4.1%), Nocardiaceae (3.5%) and Devosiaceae (2.6%), suggesting the enrichment of bacterial groups potentially adapted to hydrocarbon-impacted environments. Beyond the effects of contamination, specific differences in each site were also evident among pristine locations. ASPA2 was particularly enriched in Gemmatimonadaceae (33.5%) and Isosphaeraceae (21.7%), whereas ASPA1 showed higher relative abundances of Intrasporangiaceae (10.6%), Planococcaceae (9.4%) and Micrococcaceae (5.8%). Hill samples were characterized by a high relative abundance of Microbacteriaceae (16.4%), together with comparatively greater abundances of Chthoniobacteraceae (3.8%) and Sphingomonadaceae (3.3%).

Endosphere communities displayed a more conserved taxonomic structure, consistent with the patterns observed in beta diversity analyses (Figure 2D). A common set of dominant bacterial families was detected across all sampling sites (Figure 3D), suggesting the presence of a stable plant-associated microbiome. Among these, Microbacteriaceae and Oxalobacteraceae were the most abundant families across both pristine and contaminated environments, representing on average 25.0% and 22.0% of the endosphere community, respectively, and constituting major components of the endosphere community irrespective of site conditions. The predominance of Microbacteriaceae has been repeatedly identified as a common and abundant component of the root microbiome of Antarctic vascular plants, and has been proposed as a member of the Antarctic plant core microbiome [56,58]. Similarly, Oxalobacteraceae was reported by Znój et al. [58] as a widespread and abundant member of the *D. antarctica* root endosphere. Numerous additional families detected in the analyzed samples have also been reported as characteristic members of Antarctic plant endospheres. These include Sphingomonadaceae, Sphingobacteriaceae, Chitinophagaceae and Rhizobiaceae [56,58]. Despite this conserved background, contaminated endosphere samples exhibited a distinct compositional profile characterized by increased relative abundances of Shewanellaceae (6.2%), Flavobacteriaceae (5.8%), Devosiaceae (4.3%) and Nakamurellaceae (1.8%). Shewanellaceae showed the strongest association with contaminated conditions, being scarcely represented in pristine sites. Differences between sampling sites were also evident within endosphere communities; however, these variations were generally driven by families with relatively low abundances compared with the dominant endosphere taxa. Pseudonocardiaceae was mainly associated with Hill (11.8%), whereas Pseudomonadaceae was abundant in both Hill (13.6%) and contaminated samples (8.6%). ASPA1 showed enrichment of Rhodanobacteraceae (7.6%) and Planococcaceae (5.5%), whereas ASPA2 was enriched in Saccharimonadaceae (14.2%), Rhizobiaceae (2.2%), and Mycobacteriaceae (2.1%) relative to the other pristine sites. These observations indicate that site-related variation mainly affected the less abundant fraction of the endosphere microbiome, while the dominant community structure remained comparatively stable. In the contaminated site, this pattern may reflect the combined influence of chronic hydrocarbon exposure and local environmental conditions.

### 3.3. Differentially Abundant Taxa (LEfSe)

**Figure 4:**
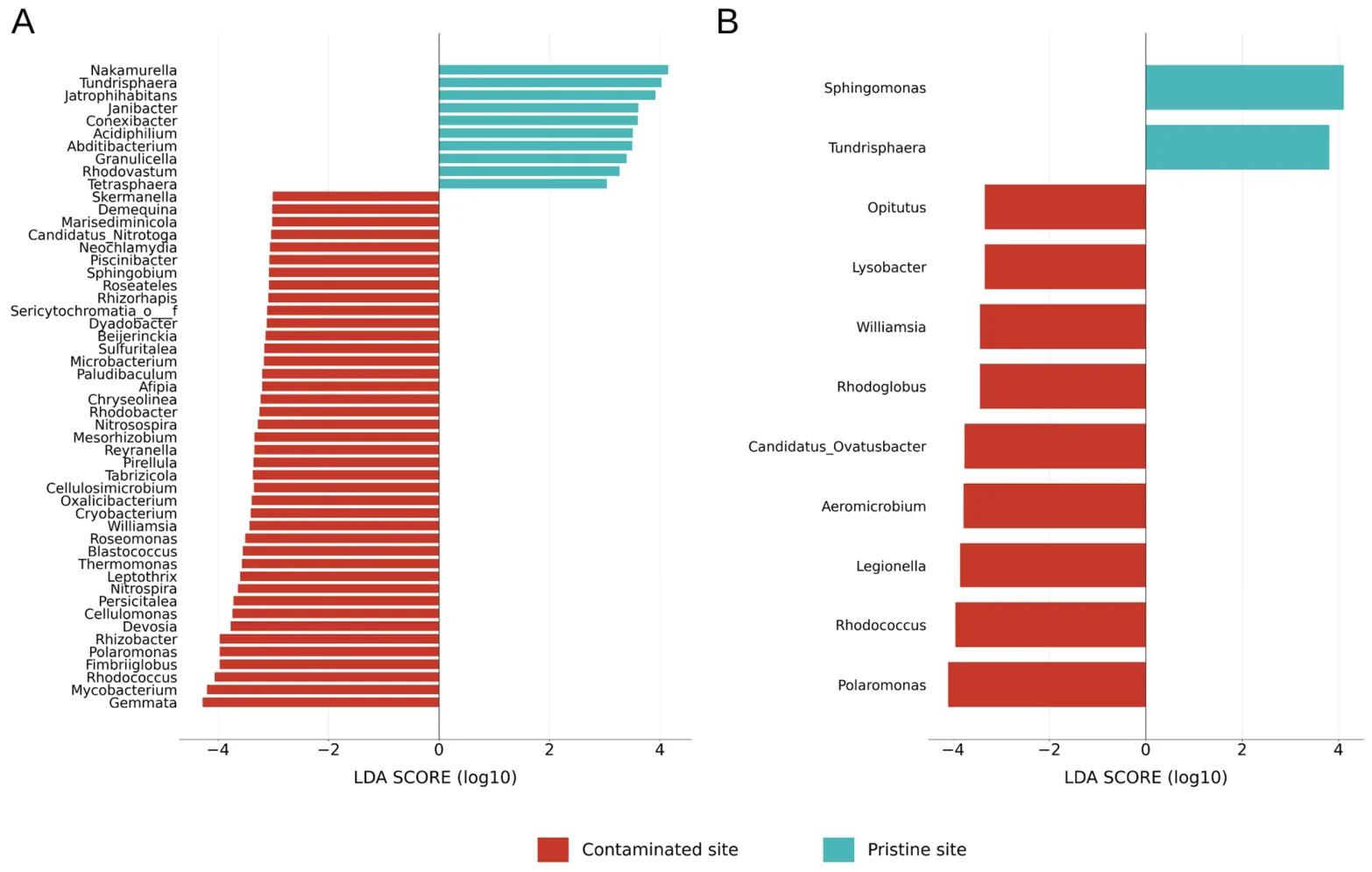
Differentially abundant bacterial genera identified by LEfSe analysis in endosphere and rhizosphere communities. (**A**) Genera significantly associated with rhizosphere samples from the contaminated site and the three pristine sites. (**B**) Genera significantly associated with endosphere samples from the contaminated site and the three pristine sites. LEfSe analysis was performed at the genus level using CPM-normalized abundances, with samples grouped as the contaminated site versus the three pristine sites combined. Taxa were retained when they met the Kruskal–Wallis and Wilcoxon significance thresholds of α = 0.01 and had an absolute LDA score ≥ 3.0. Positive LDA scores indicate genera associated with communities from the pristine sites, whereas negative scores indicate genera associated with communities from the contaminated site.

While relative abundance analyses revealed broad taxonomic differences between the contaminated site and the pristine sites, these patterns do not necessarily identify the taxa most strongly associated with each environmental condition. To identify potential microbial biomarkers, differential abundance analyses were performed using LEfSe (Figure 4). This approach revealed distinct sets of bacterial genera associated with samples from the contaminated site and the three pristine sites in both plant compartments. *Polaromonas*, *Rhodococcus* and *Williamsia* were identified as biomarkers associated with samples from the contaminated site in both rhizosphere and endosphere communities. In addition, contaminated rhizosphere communities were enriched in *Mycobacterium*, *Gemmata*, *Fimbriiglobus*, *Rhizobacter*, *Devosia*, *Cellulomonas* and *Bradyrhizobium*, whereas rhizosphere communities from the pristine site were characterized by *Nakamurella*, *Tundrisphaera*, *Janibacter* and *Conexibacter*. The endosphere exhibited a more restricted set of differentially abundant genera. In addition to *Polaromonas*, *Rhodococcus* and *Williamsia*, the endosphere community at the contaminated site was associated with *Legionella*, *Aeromicrobium*, *Candidatus Ovatusbacter*, *Rhodoglobus*, *Lysobacter* and *Opitutus*, whereas endosphere communities from the pristine sites were characterized by *Sphingomonas* and *Tundrisphaera*. Relative abundance analyses revealed the dominant bacterial groups within each community and identified major taxonomic differences between the contaminated site and the pristine sites. LEfSe complemented these observations by identifying bacterial genera that most clearly distinguished these groups of samples. Together, these approaches allowed us to distinguish taxa that were not only abundant members of the community but were also strongly associated with either the contaminated-site samples or the pristine-site samples. The combined use of community composition and differential abundance analyses has become a common strategy for identifying microbial taxa associated with contrasting sample groups [59,60,61]. Among the genera associated with the contaminated site, *Polaromonas* belongs to Comamonadaceae, a family that exhibited higher relative abundances at the contaminated site in both compartments. In contrast, *Rhodococcus* and *Williamsia* are members of Nocardiaceae, a family that remained relatively stable across rhizosphere sites and was not among the dominant families in endosphere communities. Additional genera detected in the rhizosphere community at the contaminated site included *Mycobacterium*, *Devosia* and *Gemmata*. These belong to Mycobacteriaceae, Devosiaceae and Gemmataceae, respectively, families that also exhibited higher relative abundances at the contaminated site. Their identification through both relative abundance and biomarker analyses indicates that these taxa were not only common members of the rhizosphere community at the contaminated site, but also prominent components of its taxonomic profile. This repeated association across independent analyses further supports their relevance within this community. In contrast, rhizosphere communities from the pristine sites were characterized by *Tundrisphaera* and *Janibacter*. Their respective families, Isosphaeraceae and Intrasporangiaceae, were also among the taxa enriched in pristine rhizosphere communities in the relative abundance analyses. A more limited set of differentially abundant genera was detected in endosphere communities, consistent with the comparatively conserved taxonomic structure observed in this compartment. *Rhodoglobus*, identified as a biomarker of contaminated endosphere communities, belongs to Microbacteriaceae, one of the dominant bacterial families detected across sites. Likewise, *Sphingomonas*, associated with endosphere pristine communities, belongs to Sphingomonadaceae, a family previously identified as being a characteristic component of the microbiomes from pristine environments.

### 3.4. Predicted Functional Profiles of the Core Microbiome

**Figure 5:**
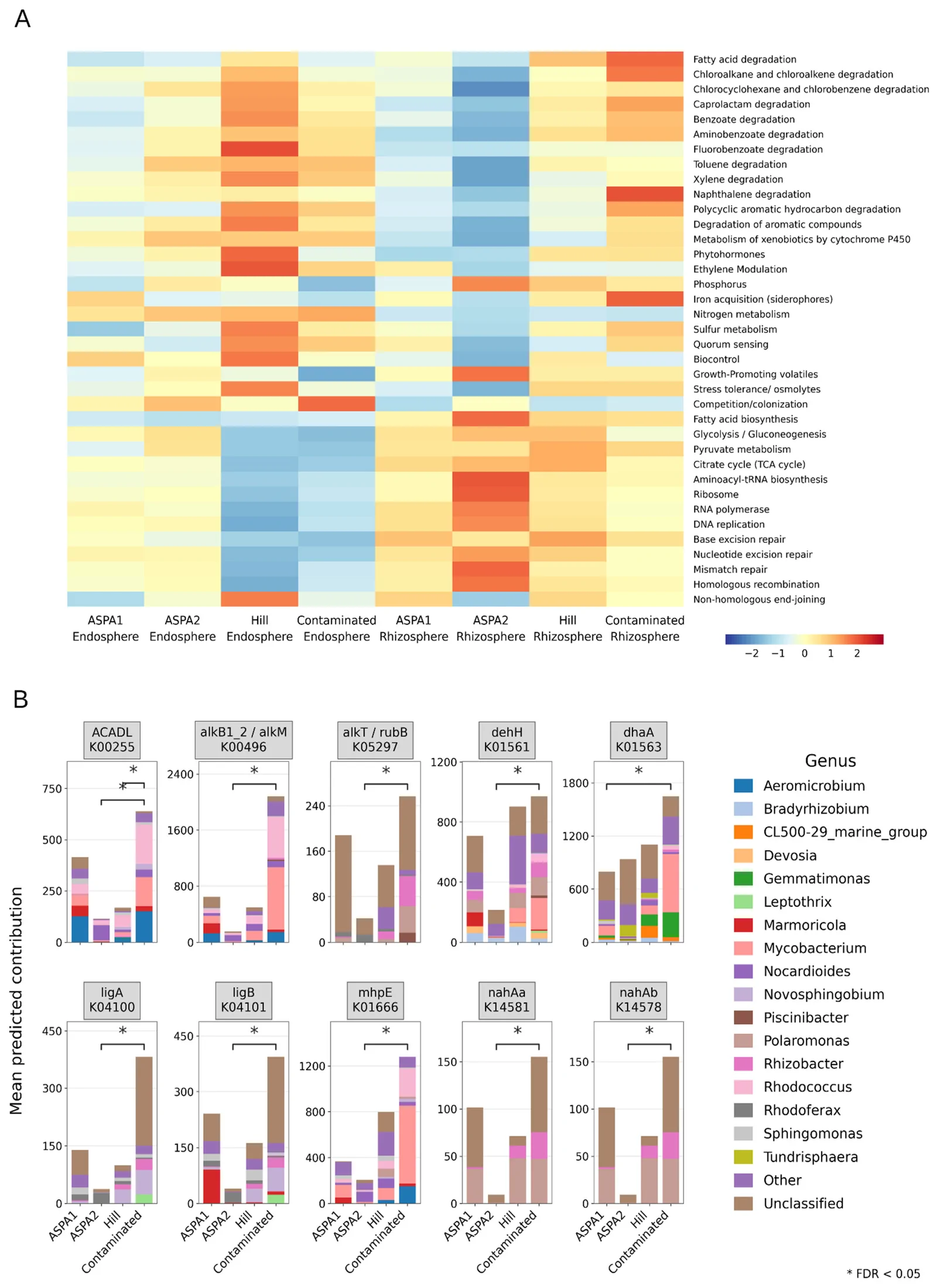
Predicted functional profiles and taxonomic contributors of the *Deschampsia antarctica* core microbiome. (**A**) Heatmap showing the relative abundance of selected KEGG pathways inferred with PICRUSt2 from core bacterial communities associated with *Deschampsia antarctica*. Pathways related to hydrocarbon degradation, xenobiotic metabolism, nutrient acquisition, plant growth promotion, stress tolerance, central metabolism and DNA repair are shown. Columns represent site–compartment combinations and rows represent predicted functional pathways. Relative abundances were log10-transformed and standardized as Z-scores across pathways prior to visualization, with warmer colors indicating higher relative abundance and cooler colors indicating lower relative abundance. Panel (**A**) provides a descriptive visualization of predicted pathway profiles. (**B**) Stacked bar plots showing the predicted contribution of the main bacterial genera to selected KEGG Orthologs (KOs) associated with hydrocarbon degradation in rhizosphere communities, inferred from stratified PICRUSt2 analyses. Bars represent mean predicted contributions for each sampling site. For each KO, the ten most abundant contributing genera were retained, whereas the remaining taxa were grouped as Other. Taxa lacking genus-level assignment were grouped as Unclassified. Differences in predicted KO abundances among sites were evaluated using Kruskal–Wallis tests followed by Dunn’s post hoc comparisons against the contaminated site, with Benjamini–Hochberg correction for multiple testing. Asterisks indicate significant differences (FDR < 0.05).

Functional prediction using PICRUSt has been widely employed to investigate microbial communities associated with phytoremediation processes and plants growing under hydrocarbon stress [62,63]. More recently, this tool has also been applied in ecological studies of Antarctic plant microbiomes, including *D. antarctica*, providing insights into their inferred metabolic potential [35,57]. PICRUSt2 predicted a total of 5845 KEGG Orthologs (KOs) across the core microbiome dataset. Weighted NSTI values averaged 0.178, with 86.7% of samples presenting values below 0.30 (Supplementary Table S4). From the complete set of predicted functions, 669 KOs associated with hydrocarbon degradation, plant growth promotion, housekeeping genes and other ecological functions of interest were selected for downstream analyses (Supplementary Table S5). The predicted functional profiles revealed clear differences between endosphere and rhizosphere communities (Figure 5A). In general, rhizosphere microbiomes exhibited higher predicted relative abundances of pathways associated with cellular growth and central metabolism, while endosphere communities displayed more conserved predicted functional profiles, with fewer pronounced differences across sites.

The strongest predicted functional signature within rhizosphere microbiomes was observed in samples from the contaminated site. These samples displayed the highest predicted abundances of pathways involved in the degradation of benzoate, naphthalene, polycyclic aromatic hydrocarbons, chlorocyclohexane, chlorobenzene, and chloroalkane degradation, together with several other xenobiotic degradation pathways. Higher predicted abundances were also observed for pathways associated with fatty acid degradation and siderophore production. Within the pristine rhizosphere communities, ASPA2 was distinguished by the highest predicted abundances of pathways associated with cellular growth and central cellular processes, including DNA replication, RNA polymerase, ribosome, aminoacyl-tRNA biosynthesis and fatty acid biosynthesis. In comparison, ASPA1 displayed a comparatively homogeneous predicted functional profile, without differences in the predicted abundances of either growth-promoting or degradation-related pathways. Hill exhibited a distinct predicted functional pattern, with relatively high abundances of pathways involved in benzoate, aminobenzoate, caprolactam and fatty acid degradation, together with sulfur metabolism. Higher predicted abundances were also observed for central metabolic pathways, including glycolysis/gluconeogenesis, pyruvate metabolism and the citrate cycle, as well as pathways associated with osmotic stress tolerance.

Endosphere communities displayed comparatively conserved predicted functional profiles across sites. However, ASPA1 and ASPA2 clustered together and exhibited similar predicted relative abundances of pathways associated with DNA replication, transcription, translation and DNA repair, including RNA polymerase, ribosome, aminoacyl-tRNA biosynthesis, mismatch repair, homologous recombination and nucleotide excision repair. Communities from Hill and contaminated locations also clustered together, displaying consistently lower predicted abundances across these housekeeping functions.

Unlike the rhizosphere community from the contaminated location, the corresponding endosphere community did not show a marked increase in the predicted representation of hydrocarbon degradation pathways. Instead, the predicted relative abundances of these pathways were generally comparable to those observed in the other endosphere groups, closely resembling that observed in ASPA2. Although the predicted functional profiles of the Hill and contaminated endosphere communities were broadly similar, the Hill endosphere showed a comparatively higher predicted representation of pathways associated with aromatic compound degradation, polycyclic aromatic hydrocarbon degradation, fluorobenzoate degradation, sulfur metabolism, quorum sensing, biocontrol and exopolysaccharide biosynthesis. In addition, similar predicted abundance patterns for sulfur metabolism, osmotic stress tolerance and biocontrol-related functions were observed in the Hill endosphere and rhizosphere. These results suggest that the taxonomic composition of both compartments at Hill may support a partially shared predicted functional potential.

The enrichment of hydrocarbon degradation pathways predicted in the contaminated rhizosphere is consistent with PICRUSt-based analyses of contaminated environments and phytoremediation systems [62,63], and suggests that this compartment may harbor a greater genomic potential for hydrocarbon degradation than the endosphere. The high predicted representation of these pathways in the rhizosphere suggests that this compartment may posses a greater genomic potential for hydrocarbon degradation than the endosphere [49,51]. Previous PICRUSt-based studies also offer an interpretation for the profile observed at ASPA2, which presented the highest concentrations of organic matter, total organic carbon and phosphorus among all sampling sites (Supplementary Table S1). Patterns of this kind have been reported in analyses of nutrient-enriched soils, where increased resource availability is associated with a greater representation of biosynthetic functions [64,65,66]. Resource-rich environments may support greater investment in transcriptional and translational machinery, thereby potentially increasing protein synthesis capacity and growth potential [67]. At the opposite end of this gradient, ASPA1 exhibited the lowest concentrations of organic matter and organic carbon, which may account for its reduced functional potential for microbial growth and metabolism.

Perhaps the most unexpected pattern was observed at the Hill location, particularly within the endosphere. Despite the absence of documented hydrocarbon contamination and TPH concentrations below the analytical limit of detection (Supplementary Table S1), both compartments at this site exhibited high predicted abundances of degradation-related pathways, while the endosphere also showed predicted abundances of several ecological and plant-associated functions. One possible interpretation is that the Hill communities harbor a distinct genomic potential shaped by local environmental conditions. Many pathways commonly associated with aromatic compound degradation also participate in the transformation of naturally occurring substrates [68], which may account for their higher predicted representation at this site.

Functional predictions based on 16S rRNA gene data should be interpreted with caution, as they depend on the availability of closely related reference genomes and provide no direct evidence of gene abundance, expression or metabolic activity [27]. Nevertheless, the relatively low NSTI values obtained in this study indicate that the predictions were generally based on taxa with reasonably close sequenced relatives, reducing some of the uncertainty associated with reference genome availability.

### 3.5. Predicted Genus-Level Contributors to Hydrocarbon-Degradation Genes

The distinctive predicted functional profiles observed in the rhizosphere of plants from the contaminated site and in the endosphere communities from Hill plants prompted a more detailed examination of individual KOs. This analysis revealed a marked contrast between compartments: 171 KOs differed significantly among rhizosphere communities from the different locations, compared with 24 in the endosphere (Kruskal–Wallis test; Supplementary Tables S6 and S7). In the rhizosphere, 85 of these remained significant after Dunn’s post hoc comparisons against the contaminated site with Benjamini–Hochberg correction, and the corresponding differences in predicted abundance were larger. Site-related variation in predicted functional profiles was therefore more evident in the rhizosphere, and subsequent analyses focused on this compartment.

Statistical comparisons identified multiple predicted gene families, represented as KEGG Orthologs (KOs), associated with hydrocarbon degradation pathways that differed significantly in predicted abundance among rhizosphere communities from the different sites. From this set, representative KOs were selected to illustrate functions associated with key pathways and stages of hydrocarbon catabolism. These included alkB, alkT and ACADL, which are associated with alkane degradation [69]; ligA, ligB and mhpE, associated with aromatic compound degradation and downstream catabolism [70,71]; nahAa and nahAb, associated with polycyclic aromatic hydrocarbon degradation [72]; and dehH and dhaA, associated with chloroalkane-chloroalkene degradation and the dehalogenation of halogenated compounds [73]. All selected KOs exhibited significantly higher predicted abundances in rhizosphere communities from the contaminated site compared with at least one of the pristine sites. However, differences in communities from plants of pristine sites varied according to the degradation pathway represented. The KOs annotated as ligA, ligB, mhpE, nahAa and nahAb frequently maintained relatively high predicted abundances in Hill communities. KOs associated with alkane degradation, represented by alkB, alkT and ACADL, displayed a stronger separation between contaminated and pristine sites, with ASPA1 generally exhibiting the second highest predicted abundances. The halogenated compound degradation KOs, dehH and dhaA, showed intermediate patterns, with predicted abundances in Hill and ASPA1 remaining higher than those observed in ASPA2.

To identify the bacterial genera potentially associated with these KOs, contributions were summarized at the genus level from stratified PICRUSt2 outputs (Figure 5B). The contribution profiles suggested that the inferred taxonomic contributions were distributed across multiple bacterial genera and varied among the KOs examined. Given that these proportions were estimated by PICRUSt2 from the taxonomic composition of the communities and the genomic information available for related reference organisms, they represent model-based predictions rather than direct measurements of the contribution of each bacterial genus.For alkane degradation, alkB was primarily associated with *Mycobacterium* (37.9%) and *Rhodococcus* (25.1%), which together represented more than 60% of the inferred contribution, while smaller proportions were assigned to *Aeromicrobium* and *Nocardioides.* The ACADL gene showed a similar inferred pattern, with major contributions from *Rhodococcus* (25.7%), *Aeromicrobium* (23.4%) and *Mycobacterium* (19.2%). In contrast, the alkT gene was mainly assigned to unclassified taxa (63.7%), followed by *Rhizobacter* (14.5%) and *Polaromonas* (12.6%). The polycyclic aromatic hydrocarbon degradation genes nahAa and nahAb displayed identical predicted taxonomic contribution profiles. Approximately half of the inferred contribution corresponded to unclassified taxa (49.2%), whereas the largest estimated contributions among classified genera were assigned to *Polaromonas* (36.4%) and *Rhizobacter* (14.4%). For aromatic compound degradation, ligA and ligB showed substantial estimated contributions from unclassified taxa (55.6% and 49.9%, respectively), followed by *Novosphingobium* (17.1% and 14.4%, respectively), *Rhizobacter*, *Sphingomonas* and *Rhodoferax*. As observed for KOs associated with alkane degradation, the largest estimated contributions to mhpE were assigned to *Mycobacterium* (41.7%) and *Rhodococcus* (15.4%), followed by *Aeromicrobium* (8.4%) and *Nocardioides* (6.6%). The halogenated compound degradation genes also displayed distinct inferred taxonomic contribution profiles. For the KO annotated as dehH, unclassified taxa represented the largest estimated contribution (28.2%), while the largest predicted contributions among classified genera were assigned to *Mycobacterium* (12.2%), *Polaromonas* (11.8%), *Rhizobacter* (8.1%), *Bradyrhizobium* (6.6%) and *Devosia* (4.3%). The predicted contribution to dhaA was more broadly distributed across taxa, with unclassified taxa (27.6%) and *Mycobacterium* (25.5%) representing the largest estimated contributions, followed by *Gemmatimonas* (11.8%) and several genera with smaller predicted contributions. The higher predicted abundances of hydrocarbon degradation pathways observed in rhizosphere communities from the contaminated location were also reflected in the predicted abundance patterns of individual KOs, indicating that the functional differences detected at the pathway level were also reflected in specific degradation-related orthologs. Notably, the selected KOs were associated with different stages of hydrocarbon catabolism, including substrate activation, aromatic ring transformation and downstream degradation processes, suggesting that rhizosphere communities from the contaminated location contained a broader predicted set of KOs associated with different stages of hydrocarbon degradation. Although these communities exhibited the highest predicted abundances, some KOs involved in aromatic compound and polycyclic aromatic hydrocarbon degradation also maintained relatively high predicted abundances in Hill rhizosphere communities. This pattern is consistent with the higher predicted abundances of degradation-related pathways previously observed in the Hill functional profile, although the highest predicted values were observed in rhizosphere communities from the contaminated location. Taxonomic contribution analyses suggested that KOs associated with different degradation pathways were assigned to distinct groups of microorganisms. KOs associated with alkane degradation were predicted to be mainly linked to *Mycobacterium* and *Rhodococcus*, whereas polycyclic aromatic hydrocarbon degradation genes were mainly associated with *Polaromonas*. A markedly different inferred taxonomic profile was observed for ligA and ligB, which were largely assigned to *Novosphingobium* and *Sphingomonas*, whereas mhpE, associated with downstream catabolism, showed a profile comparable to that of the alkane-degradation KOs. In contrast, the predicted contributions to KOs associated with halogenated compound degradation were distributed across a broader range of taxa, including *Mycobacterium*, *Polaromonas*, *Rhizobacter*, *Bradyrhizobium* and *Devosia*. These results suggest that the predicted degradation-related KOs were distributed across a broad range of bacterial genera within the rhizosphere microbiome. However, because these associations were inferred from PICRUSt2 predictions, they represent putative functional potential and would require complementary approaches to verify gene presence, expression, and the contribution of these taxa to hydrocarbon degradation in situ. A notable proportion of the predicted contribution to several degradation related KOs were assigned to unclassified taxa. This pattern was particularly evident for alkT, nahAa, nahAb, ligA and ligB, where unclassified microorganisms accounted for approximately half or more of the estimated contribution. The recurrent assignment of predicted contributions to unclassified taxa across multiple degradation pathways further highlights that additional hydrocarbon degrading populations may remain undescribed. Interestingly, some of the genera predicted to contribute to degradation-related KOs were also associated with KOs related to plant growth-promoting traits (Supplementary Figure S1). Compared with the numerous degradation-related genes enriched in rhizosphere communities from contaminated locations, only a limited number of PGPR-related KOs showed higher predicted abundances under contaminated conditions. These KOs were associated with nitrogen fixation, siderophore production, osmotic stress tolerance, quorum sensing, phytohormone-related metabolism and the production of growth-promoting volatile compounds. Bradyrhizobium was predicted to contribute to KOs associated with nitrogen fixation, whereas *Gemmatimonas* showed the largest estimated contribution to KOs associated with siderophore biosynthesis. Rhizobacter was mainly associated with predicted contributions to quorum sensing KOs. *Mycobacterium* and *Rhodococcus*, however, were associated with a broader range of predicted functions, including KOs related to osmotic stress tolerance, volatile compound production and phytohormone-related metabolism.

Two limitations should be considered when interpreting culture-independent results. Because only one chronically contaminated location supporting established *D. antarctica* stands was available for sampling, contamination status was inherently linked to site identity. Consequently, the taxonomic and predicted functional patterns observed at this site cannot be attributed exclusively to hydrocarbon contamination and may also reflect other local environmental characteristics, including soil physicochemical properties. A further limitation concerns the level of biological replication. The number of plants that could be sampled was set by the Antarctic environmental permits under which the study was carried out. Although this limits the resolution of the analyses, it does not affect the robustness of the observed patterns: the differences reported here were detected using conservative statistical criteria and represent the most pronounced contrasts among sites. The compositional differentiation of the contaminated-site rhizosphere and the taxa consistently associated with it are therefore well supported, while the finer structure of variation among sites remains to be resolved with more extensive sampling.

### 3.6. Candidate Taxa for Microbe-Assisted Phytoremediation

The culture-independent analyses identified the rhizosphere as the compartment where site of origin had the strongest effect on community structure. Within this compartment, the community from the chronically contaminated site was separated from the three pristine sites along the dominant ordination axis and was characterized by a distinct set of associated genera. It is also the only rhizosphere community sampled in this study recovered in soil with detectable hydrocarbon concentrations, and therefore the only one in which bacteria were subject to sustained selection under hydrocarbon exposure. The genera consistently associated with it across the different analyses performed in this study were examined in light of previous studies to assess their potential relevance for microbe-assisted phytoremediation.

*Polaromonas* emerged as one of the most relevant candidates. The genus was identified as a biomarker of the contaminated site in both plant compartments and was predicted to contribute substantially to polycyclic aromatic hydrocarbon (PAH) degradation pathways. *Polaromonas* is a psychrotolerant genus widely distributed in polar environments and recognized for its metabolic versatility under cold conditions [74]. Hydrocarbon and chlorinated compound degradation capabilities have been reported for different members of the genus, including *Polaromonas naphthalenivorans*, a naphthalene degrader, and *Polaromonas* sp. JS666, which harbors pathways involved in haloalkane degradation and aromatic compound catabolism [75,76]. In addition to its catabolic potential, *Polaromonas* has been associated with nitrogen fixation in Arctic glacier forefields [74] *Polaromonas* has also been reported as a dominant bacterial taxon in hydrocarbon contaminated soils from 25 de Mayo (King George) Island [77], which provides additional support for its ecological relevance in Antarctic environments affected by petroleum contamination.

*Rhodococcus* was another genus repeatedly highlighted across the different analyses performed in this study. In addition to being identified as a biomarker of the contaminated site in both plant compartments, *Rhodococcus* was associated with one of the broadest predicted functional repertoires, contributing not only to hydrocarbon degradation related functions but also to traits linked to osmotic stress tolerance, volatile compound production and phytohormone related metabolism. These observations are consistent with previous studies describing *Rhodococcus* as a highly versatile bacterial genus whose ecological success has been attributed to its environmental persistence, robustness and genomic plasticity, enabling adaptation to changing environmental conditions and pollutant exposure [78,79]. As a consequence, *Rhodococcus* species possess an extensive catabolic repertoire and can degrade an exceptionally diverse array of hydrocarbon and aromatic pollutants, including BTEX compounds, polycyclic aromatic hydrocarbons and nitrogen containing heterocyclic compounds [80]. Given its remarkable hydrocarbon degrading capabilities, it is not surprising that members of this genus have repeatedly been associated with hydrocarbon degradation in Antarctic environments. The role of *Rhodococcus* under Antarctic conditions has been documented for more than two decades. One of the earliest reports was provided by Bej et al. [81], who isolated *Rhodococcus* strains from soils surrounding Scott Base and demonstrated their ability to utilize alkanes ranging from C6 to C20. Further evidence of the ecological relevance of this genus was provided by Ruberto et al. [82] who demonstrated a positive effect of bioaugmentation with an indigenous alkane degrading *Rhodococcus* strain on diesel fuel biodegradation. In addition to their hydrocarbon degrading capabilities, *Rhodococcus* strains are also able to produce biosurfactants in response to water insoluble substrates such as hydrocarbons [79]. This trait has also been reported in Antarctic isolates, including *Rhodococcus* sp. ADL36, which produces a highly stable lipopeptide biosurfactant with strong emulsifying activity [83].

*Mycobacterium* was another genus consistently associated with rhizosphere communities from the contaminated site. In addition to being identified as a biomarker of this site, members of this genus were among the principal predicted contributors to several of the hydrocarbon-degradation genes examined, including those associated with alkane degradation and with downstream aromatic catabolism. These observations are consistent with previous studies describing *Mycobacterium* as an important component of hydrocarbon degrading communities. Members of the genus *Mycobacterium* have been reported to degrade polycyclic aromatic hydrocarbons ranging from naphthalene to high molecular weight compounds such as pyrene [84]. PAH degrading *Mycobacterium* strains have also been shown to colonize plant roots, utilize root exudates and actively mineralize aromatic hydrocarbons in the rhizosphere [85]. Similar observations have been reported in Arctic plant rhizospheres, where *Mycobacterium* was identified among hydrocarbon degrading bacterial communities harboring *alkB* genes [86].

*Devosia* was also consistently associated with rhizosphere communities from the contaminated site. Unlike *Rhodococcus* and *Mycobacterium*, whose roles in hydrocarbon degradation have been extensively documented, the ecological significance of *Devosia* in contaminated environments only recently started to emerge. A comparative genomic analysis revealed that genes associated with aromatic compound degradation are broadly distributed across *Devosia* genomes, indicating that the utilization of these substrates may represent a common metabolic feature within the genus [87]. Supporting this observation, Wang et al. [88] described a *Devosia* strain isolated from rhizosphere soil capable of degrading two high molecular weight polycyclic aromatic hydrocarbons, pyrene and benzo[a]pyrene. Moreover, its predicted contribution to halogenated compound degradation in the present study suggests a potential role in the transformation of these substrates under Antarctic conditions.

Collectively, these observations provided the ecological and functional context for the subsequent cultivation efforts. During the following Antarctic campaign, we evaluated whether cultivable representatives of these candidate genera could be recovered from the rhizosphere of *D. antarctica* at the contaminated site, with the aim of establishing a collection of candidate strains for further physiological characterization and experimental assessment. A total of 187 bacterial isolates representing 38 genera were recovered.

**Figure 6:**
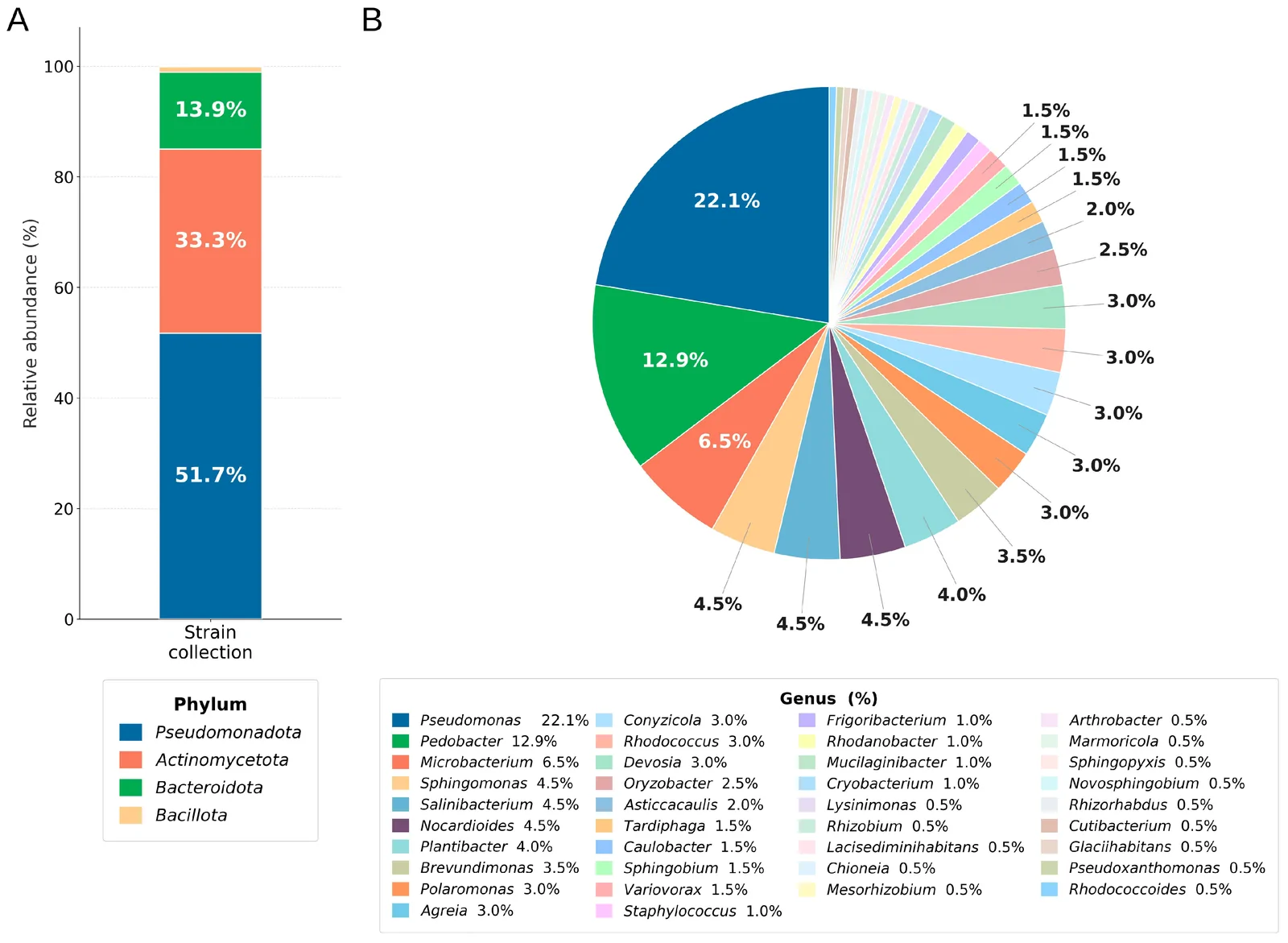
Taxonomic composition of cultivable bacterial isolates recovered from the rhizosphere of *Deschampsia antarctica* growing in hydrocarbon-contaminated soil. (**A**) Relative abundance of bacterial phyla represented in the culture collection. (**B**) Relative abundance of bacterial genera recovered from rhizosphere samples. Percentages indicate the proportion of isolates assigned to each taxonomic group within the collection.

The large number of bacterial isolates recovered enabled comparison between the cultured collection and the bacterial community characterized through culture-independent analyses. As expected, the cultured collection represented only a subset of the taxonomic diversity detected by sequencing (Figure 3A and Figure 6A). Given that the two datasets were generated from samples collected during consecutive Antarctic summer campaigns, the comparison reflects taxonomic correspondence across independent sampling events rather than direct recovery of taxa from the sequenced samples; interannual variability in these communities was not assessed. Nevertheless, the dominant bacterial phyla recovered by cultivation corresponded to those identified by the culture-independent analysis of the contaminated rhizosphere. In the sequencing dataset, Actinomycetota and Pseudomonadota represented 36.8% and 26.9% of the mean relative abundance, respectively, whereas in the cultured collection they accounted for 33.3% and 51.7%, respectively. Thus, although Actinomycetota predominated in the sequencing dataset and Pseudomonadota in the cultured collection, both phyla represented the major bacterial lineages detected by the two approaches. Therefore, although cultivation did not reproduce the full complexity of the bacterial communities, it recovered representative members of the bacterial community associated with *D. antarctica*, suggesting that additional isolation efforts could further increase the diversity of cultivable taxa.

More importantly, the integration of culture-independent (top-down) and culture-dependent (bottom-up) approaches provided a framework for identifying candidate bacterial taxa for microbe-assisted phytoremediation. Ecological and predicted functional patterns identified through culture-independent analyses serve as the basis for strain selection, while cultivation enables the recovery of candidate microorganisms [89]. As a result, *Polaromonas*, *Rhodococcus* and *Devosia* emerged as the strongest candidates because they fulfilled three complementary criteria: they were highlighted by the culture-independent analyses of the contaminated-site rhizosphere, recovered as bacterial isolates during the subsequent field campaign (Figure 6B), and previously reported to possess traits related to hydrocarbon degradation or adaptation to cold contaminated environments. These genera therefore represent an appropriate starting point for subsequent physiological characterization and experimental evaluation.

## 4. Conclusions

Using a combination of culture-independent and culture-dependent approaches, this study provides a first ecological characterization of the bacterial communities associated with *Deschampsia antarctica* in chronically diesel-contaminated and pristine Antarctic soils. The rhizosphere and the endosphere emerged as complementary compartments: the rhizosphere, closely associated with soil properties and showing marked differences at the contaminated site, harbored a diverse community with higher predicted representation of hydrocarbon degradation-related pathways, whereas the host-selected endosphere supported a distinct and less diverse bacterial assemblage potentially relevant to plant fitness. The combined taxonomic, biomarker and predicted functional analyses highlighted *Polaromonas*, *Rhodococcus*, *Mycobacterium* and *Devosia* as taxa associated with the rhizosphere at the contaminated site, three of which were also recovered in culture during the subsequent Antarctic campaign. These cold-adapted genera represent realistic priority targets for sequencing-guided cultivation and future experimental characterization. Their identification, together with the recovery of cultivable representatives, constitutes a first step toward the development of an integrated, microbe-assisted phytoremediation strategy for Antarctic environments.

## Data Availability

The 16S rRNA gene amplicon sequencing data generated in this study have been deposited in the NCBI Sequence Read Archive SRA under BioProject accession number PRJNA1483961.

## Supplementary Materials

The following supporting information can be downloaded at: https://www.mdpi.com/article/10.3390/microorganisms14091917/s1, Table S1: Soil physicochemical characteristics and total petroleum hydrocarbon concentrations of the four sampling sites on 25 de Mayo (King George) Island; Table S2: Sampling design and number of samples collected during the Antarctic summer campaign 2019/2020 from the four sampling sites on 25 de Mayo (King George) Island; Table S3: Complete list of core ASVs identified for each site–compartment group and their taxonomic assignments; Table S4: Weighted nearest sequenced taxon index (NSTI) values obtained from PICRUSt2 for each sample; Table S5: Curated subset of 669 KEGG Orthologs (KOs) included in the predicted functional analysis; Table S6: Differentially abundant KEGG Orthologs (KOs) identified in rhizosphere communities; Table S7: KEGG Orthologs showing differences among endosphere communities (Kruskal–Wallis test, uncorrected *p* < 0.05); Figure S1: Taxonomic contributors to selected plant growth-promoting (PGPR) functions predicted by PICRUSt2 in rhizosphere communities. Stacked bar plots show the mean genus-level contribution to the predicted abundance of significant PGPR-related KOs across sampling sites (ASPA1, ASPA2, Hill and Contaminated).
