# Supplementary material for: Bacterial Communities Associated with Deschampsia antarctica in Chronically Diesel-Contaminated Soils: Candidate Taxa for Microbe-Assisted Phytoremediation
Source: Microorganisms. 2026 Aug 31;14(9):1917. doi: 10.3390/microorganisms14091917 (PMC13609677; doi:10.3390/microorganisms14091917)
Supplement: Supplementary file 1 [file microorganisms-14-01917-s001.zip › microorganisms-4450742-supplementary.pdf]

Bacterial Communities Associated with Deschampsia antarctica in Chronically Diesel-Contaminated Soils: Candidate Taxa for Microbe-Assisted Phytoremediation

Camila Tassano [1], Matías Javier Garavaglia [2], Marcos Leopoldo Esteso [1,3,4], Catalina Basile Dazzi [1,3,4,5], Juan Orlowski [3,4], Walter Patricio Mac Cormack [1], Jaco Vangronsveld [5,6], Sofie Thijs [5], Lucas Adolfo Mauro Ruberto [1,3,4,*] and Francisco Massot [1,3,4,*]

[1] Instituto Antártico Argentino, San Martín 1650, Buenos Aires, Argentina; camilatassano@gmail.com (C.T.)
[2] Laboratorio de Aplicaciones Biotecnológicas y Microbiología, Secretaría de Investigación, Universidad Nacional de Hurlingham, Hurlingham 1688, Buenos Aires, Argentina; matias.garavaglia@unahur.edu.ar
[3] Instituto de Nanobiotecnología, Facultad de Farmacia y Bioquímica, Universidad de Buenos Aires–CONICET, Ciudad Autónoma de Buenos Aires 1113, Buenos Aires, Argentina
[4] Consejo Nacional de Investigaciones Científicas y Técnicas, Buenos Aires 1425, Buenos Aires, Argentina
[5] Environmental Microbiology, Centre for Environmental Sciences, Department of Biology, Faculty of Sciences, Hasselt University, 3590 Diepenbeek, Belgium
[6] Department of Plant Physiology and Biophysics, Institute of Biological Sciences, Maria Curie-Skłodowska University, 20-031 Lublin, Poland
* Correspondence: ulr@mrecic.gov.ar (L.A.M.R.); fkx@mrecic.gov.ar (F.M.)

Table S1. Soil physicochemical characteristics and total petroleum hydrocarbon concentrations of the four sampling sites on 25 de Mayo (King George) Island.

TOC, total organic carbon; OM, organic matter; Available P, available phosphorus; TPH, total petroleum hydrocarbons; LD, limit of detection.

Site	TOC (%)	OM (%)	Available P (mg kg^{-1})	Clay (%)	Silt (%)	Sand (%)	Textural class	TPH (mg kg^{-1})
Hill	1.22	2.22	19.91	6.25	11.25	82.50	Sandy loam	< LD
ASPA1	0.19	0.34	54.35	2.50	1.25	96.25	Sand	< LD
ASPA2	5.77	10.50	525.64	1.00	1.25	97.50	Sand	< LD
Contaminated	2.42	4.40	56.25	1.25	5.00	93.75	Sand	900 ± 50

Table S2. Sampling design and number of samples collected during the Antarctic summer campaign 2019/2020 from the four sampling sites on 25 de Mayo (King George) Island.

Rhizosphere and root endosphere samples were obtained from each sampled plant. ASPA, Antarctic Specially Protected Area.

Site	Rhizosphere samples (n)	Root endosphere samples (n)	Total samples (n)
Hill	3	3	6
ASPA1	3	3	6
ASPA2	3	3	6
Contaminated	6	6	12
**Total**	**15**	**15**	**30**

Table S3. Complete list of core ASVs identified for each site–compartment group and their taxonomic assignments

Core ASVs were defined as ASVs present in at least 50% of samples within each site-compartment group and with a mean relative abundance of at least 0.1%.

Site	Compartment	Genus	Core ASV
ASPA1	Endosphere	Sporosarcina	9
ASPA1	Endosphere	Parafrigoribacterium	8
ASPA1	Endosphere	Herbaspirillum	6
ASPA1	Endosphere	Devosia	4
ASPA1	Endosphere	Galbitalea	3
ASPA1	Endosphere	Mucilaginibacter	3
ASPA1	Endosphere	Nakamurella	3
ASPA1	Endosphere	Rhodanobacter	3
ASPA1	Endosphere	Cryobacterium	2
ASPA1	Endosphere	Mycobacterium	2
ASPA1	Endosphere	Sphingomonas	2
ASPA1	Endosphere	Tundrisphaera	2
ASPA1	Endosphere	Undibacterium	2
ASPA1	Endosphere	Acidovorax	1
ASPA1	Endosphere	Aeromicrobium	1
ASPA1	Endosphere	Allorhizobium-Neorhizobium-Pararhizobium-Rhizobium	1
ASPA1	Endosphere	Arthrobacter	1
ASPA1	Endosphere	Bacillus	1
ASPA1	Endosphere	Candidatus_Protochlamydia	1
ASPA1	Endosphere	Cellulomonas	1
ASPA1	Endosphere	Chryseobacterium	1
ASPA1	Endosphere	Clostridium_sensu_stricto_13	1
ASPA1	Endosphere	Conexibacter	1
ASPA1	Endosphere	Cutibacterium	1
ASPA1	Endosphere	Edaphobaculum	1
ASPA1	Endosphere	Flavobacterium	1
ASPA1	Endosphere	Kocuria	1
ASPA1	Endosphere	Luteolibacter	1

ASPA1	Endosphere	Marmoricola	1
ASPA1	Endosphere	Microbacterium	1
ASPA1	Endosphere	Novosphingobium	1
ASPA1	Endosphere	Pseudarthrobacter	1
ASPA1	Endosphere	Rhodoferax	1
ASPA1	Endosphere	Rhodopseudomonas	1
ASPA1	Endosphere	Shinella	1
ASPA1	Endosphere	Simplicispira	1
ASPA1	Endosphere	TM7a	1
ASPA1	Endosphere	s3t2d-1089	1
ASPA1	Rhizosphere	Sporosarcina	15
ASPA1	Rhizosphere	Parafrigoribacterium	10
ASPA1	Rhizosphere	Arthrobacter	9
ASPA1	Rhizosphere	Nakamurella	6
ASPA1	Rhizosphere	Marmoricola	5
ASPA1	Rhizosphere	Candidatus_Udaeobacter	4
ASPA1	Rhizosphere	Phycicoccus	4
ASPA1	Rhizosphere	Rhodanobacter	4
ASPA1	Rhizosphere	Flavobacterium	3
ASPA1	Rhizosphere	Iamia	3
ASPA1	Rhizosphere	Janibacter	3
ASPA1	Rhizosphere	Luteolibacter	3
ASPA1	Rhizosphere	Mucilaginibacter	3
ASPA1	Rhizosphere	Pseudarthrobacter	3
ASPA1	Rhizosphere	Aeromicrobium	2
ASPA1	Rhizosphere	Aquisphaera	2
ASPA1	Rhizosphere	Cryobacterium	2
ASPA1	Rhizosphere	Deinococcus	2
ASPA1	Rhizosphere	Gelidibacter	2
ASPA1	Rhizosphere	Humibacillus	2

ASPA1	Rhizosphere	Lysobacter	2
ASPA1	Rhizosphere	Massilia	2
ASPA1	Rhizosphere	Nocardioides	2
ASPA1	Rhizosphere	Polaromonas	2
ASPA1	Rhizosphere	Renibacterium	2
ASPA1	Rhizosphere	Simplicispira	2
ASPA1	Rhizosphere	Sphingomonas	2
ASPA1	Rhizosphere	Tundrisphaera	2
ASPA1	Rhizosphere	Allobranchiibius	1
ASPA1	Rhizosphere	Antricoccus	1
ASPA1	Rhizosphere	Arsenicitalea	1
ASPA1	Rhizosphere	Bradyrhizobium	1
ASPA1	Rhizosphere	Candidatus_Nostocoida	1
ASPA1	Rhizosphere	Clostridium_sensu_stricto_13	1
ASPA1	Rhizosphere	Devosia	1
ASPA1	Rhizosphere	Dokdonella	1
ASPA1	Rhizosphere	Galbitalea	1
ASPA1	Rhizosphere	Gemmatimonas	1
ASPA1	Rhizosphere	Intrasporangium	1
ASPA1	Rhizosphere	Mesorhizobium	1
ASPA1	Rhizosphere	Microbacterium	1
ASPA1	Rhizosphere	Mycobacterium	1
ASPA1	Rhizosphere	Oryzihumus	1
ASPA1	Rhizosphere	Paludisphaera	1
ASPA1	Rhizosphere	Parasegetibacter	1
ASPA1	Rhizosphere	Rhizobacter	1
ASPA1	Rhizosphere	Rhodococcus	1
ASPA1	Rhizosphere	Rhodoferax	1
ASPA1	Rhizosphere	Rhodopseudomonas	1
ASPA1	Rhizosphere	Rugamonas	1

ASPA1	Rhizosphere	TM7a	1
ASPA1	Rhizosphere	Tetrasphaera	1
ASPA1	Rhizosphere	Thermomonas	1
ASPA2	Endosphere	Parafrigoribacterium	6
ASPA2	Endosphere	Mycobacterium	4
ASPA2	Endosphere	Rhodanobacter	4
ASPA2	Endosphere	Herbaspirillum	3
ASPA2	Endosphere	Mucilaginibacter	3
ASPA2	Endosphere	Sphingomonas	3
ASPA2	Endosphere	TM7a	3
ASPA2	Endosphere	Galbitalea	2
ASPA2	Endosphere	Ralstonia	2
ASPA2	Endosphere	Tundrisphaera	2
ASPA2	Endosphere	Undibacterium	2
ASPA2	Endosphere	Allorhizobium-Neorhizobium-Pararhizobium-Rhizobium	1
ASPA2	Endosphere	Arthrobacter	1
ASPA2	Endosphere	Bacillus	1
ASPA2	Endosphere	Chryseobacterium	1
ASPA2	Endosphere	Clostridium_sensu_stricto_13	1
ASPA2	Endosphere	Corynebacterium	1
ASPA2	Endosphere	Cutibacterium	1
ASPA2	Endosphere	Dokdonella	1
ASPA2	Endosphere	Kocuria	1
ASPA2	Endosphere	Microbacterium	1
ASPA2	Endosphere	Nakamurella	1
ASPA2	Endosphere	Paludisphaera	1
ASPA2	Endosphere	Paralcaligenes	1
ASPA2	Endosphere	Rhodoferax	1
ASPA2	Endosphere	Rhodopseudomonas	1

ASPA2	Endosphere	Singulisphaera	1
ASPA2	Endosphere	Sporosarcina	1
ASPA2	Endosphere	Taibaiella	1
ASPA2	Rhizosphere	Gemmatimonas	18
ASPA2	Rhizosphere	Tundrisphaera	9
ASPA2	Rhizosphere	Candidatus_Nostocoida	8
ASPA2	Rhizosphere	Nakamurella	7
ASPA2	Rhizosphere	Arthrobacter	6
ASPA2	Rhizosphere	Nocardioides	4
ASPA2	Rhizosphere	Jatrophihabitans	3
ASPA2	Rhizosphere	Parafrigoribacterium	3
ASPA2	Rhizosphere	Aquisphaera	2
ASPA2	Rhizosphere	Dokdonella	2
ASPA2	Rhizosphere	Granulicella	2
ASPA2	Rhizosphere	Longivirga	2
ASPA2	Rhizosphere	Rhodanobacter	2
ASPA2	Rhizosphere	Rhodovastum	2
ASPA2	Rhizosphere	Singulisphaera	2
ASPA2	Rhizosphere	Abditibacterium	1
ASPA2	Rhizosphere	Acidipila	1
ASPA2	Rhizosphere	Acidothermus	1
ASPA2	Rhizosphere	Bradyrhizobium	1
ASPA2	Rhizosphere	Bryobacter	1
ASPA2	Rhizosphere	Candidatus_Solibacter	1
ASPA2	Rhizosphere	Diaminobutyricibacter	1
ASPA2	Rhizosphere	Humibacillus	1
ASPA2	Rhizosphere	Luteolibacter	1
ASPA2	Rhizosphere	Oryzihumus	1
ASPA2	Rhizosphere	Renibacterium	1
ASPA2	Rhizosphere	Rhizobacter	1

ASPA2	Rhizosphere	Rhodococcus	1
ASPA2	Rhizosphere	Rhodoferax	1
Contaminated	Endosphere	Herbaspirillum	6
Contaminated	Endosphere	Flavobacterium	4
Contaminated	Endosphere	Nakamurella	3
Contaminated	Endosphere	Pseudomonas	3
Contaminated	Endosphere	Rhodococcus	3
Contaminated	Endosphere	Rhodoferax	3
Contaminated	Endosphere	Undibacterium	3
Contaminated	Endosphere	Devosia	2
Contaminated	Endosphere	Duganella	2
Contaminated	Endosphere	Galbitalea	2
Contaminated	Endosphere	Mucilaginibacter	2
Contaminated	Endosphere	Mycobacterium	2
Contaminated	Endosphere	Parafrigoribacterium	2
Contaminated	Endosphere	Polaromonas	2
Contaminated	Endosphere	Shewanella	2
Contaminated	Endosphere	Acidovorax	1
Contaminated	Endosphere	Asticcacaulis	1
Contaminated	Endosphere	Aureimonas	1
Contaminated	Endosphere	Bacillus	1
Contaminated	Endosphere	Brevibacillus	1
Contaminated	Endosphere	Candidatus_Symbiobacter	1
Contaminated	Endosphere	Caulobacter	1
Contaminated	Endosphere	Cellulomonas	1
Contaminated	Endosphere	Chryseobacterium	1
Contaminated	Endosphere	Clostridium_sensu_stricto_13	1
Contaminated	Endosphere	Cutibacterium	1
Contaminated	Endosphere	Kocuria	1
Contaminated	Endosphere	Legionella	1

Contaminated	Endosphere	Novosphingobium	1
Contaminated	Endosphere	Nubsella	1
Contaminated	Endosphere	Pedobacter	1
Contaminated	Endosphere	Ralstonia	1
Contaminated	Endosphere	Sphingobium	1
Contaminated	Endosphere	Sphingomonas	1
Contaminated	Endosphere	Staphylococcus	1
Contaminated	Endosphere	Variovorax	1
Contaminated	Endosphere	Williamsia	1
Contaminated	Endosphere	s3t2d-1089	1
Contaminated	Rhizosphere	Gemmatimonas	13
Contaminated	Rhizosphere	Mycobacterium	8
Contaminated	Rhizosphere	Polaromonas	7
Contaminated	Rhizosphere	Rhodococcus	6
Contaminated	Rhizosphere	Gemmata	5
Contaminated	Rhizosphere	CL500-29_marine_group	4
Contaminated	Rhizosphere	Devosia	4
Contaminated	Rhizosphere	Nakamurella	4
Contaminated	Rhizosphere	Parafrigoribacterium	4
Contaminated	Rhizosphere	Rhizobacter	4
Contaminated	Rhizosphere	Cellulomonas	3
Contaminated	Rhizosphere	Cryobacterium	3
Contaminated	Rhizosphere	Mesorhizobium	3
Contaminated	Rhizosphere	Nocardioides	3
Contaminated	Rhizosphere	Paludisphaera	3
Contaminated	Rhizosphere	Aeromicrobium	2
Contaminated	Rhizosphere	Cellulosimicrobium	2
Contaminated	Rhizosphere	Ferruginibacter	2
Contaminated	Rhizosphere	Gaiella	2
Contaminated	Rhizosphere	Galbitalea	2

Contaminated	Rhizosphere	Humibacillus	2
Contaminated	Rhizosphere	KCM-B-112	2
Contaminated	Rhizosphere	Leptothrix	2
Contaminated	Rhizosphere	Lysobacter	2
Contaminated	Rhizosphere	Nitrospira	2
Contaminated	Rhizosphere	Oryzihumus	2
Contaminated	Rhizosphere	Roseomonas	2
Contaminated	Rhizosphere	AAP99	1
Contaminated	Rhizosphere	Afipia	1
Contaminated	Rhizosphere	Arenimonas	1
Contaminated	Rhizosphere	Beijerinckia	1
Contaminated	Rhizosphere	Blastococcus	1
Contaminated	Rhizosphere	Bradyrhizobium	1
Contaminated	Rhizosphere	Bryobacter	1
Contaminated	Rhizosphere	Chryseolinea	1
Contaminated	Rhizosphere	Conexibacter	1
Contaminated	Rhizosphere	Diaminobutyricibacter	1
Contaminated	Rhizosphere	Dokdonella	1
Contaminated	Rhizosphere	Ellin6067	1
Contaminated	Rhizosphere	Fimbriiglobus	1
Contaminated	Rhizosphere	Flavobacterium	1
Contaminated	Rhizosphere	Iamia	1
Contaminated	Rhizosphere	Jatrophihabitans	1
Contaminated	Rhizosphere	Luteolibacter	1
Contaminated	Rhizosphere	Marmoricola	1
Contaminated	Rhizosphere	Microbacterium	1
Contaminated	Rhizosphere	Novosphingobium	1
Contaminated	Rhizosphere	Persicitalea	1
Contaminated	Rhizosphere	Phycicoccus	1
Contaminated	Rhizosphere	Piscinibacter	1

Contaminated	Rhizosphere	Pseudarthrobacter	1
Contaminated	Rhizosphere	Pseudorhodobacter	1
Contaminated	Rhizosphere	Pseudoxanthomonas	1
Contaminated	Rhizosphere	Rhodanobacter	1
Contaminated	Rhizosphere	Rhodobacter	1
Contaminated	Rhizosphere	Skermanella	1
Contaminated	Rhizosphere	Sphingomonas	1
Contaminated	Rhizosphere	Tabrizicola	1
Contaminated	Rhizosphere	Williamsia	1
Hill	Endosphere	Herbaspirillum	3
Hill	Endosphere	Sphingomonas	2
Hill	Endosphere	Allorhizobium-Neorhizobium-Pararhizobium-Rhizobium	1
Hill	Endosphere	Bacillus	1
Hill	Endosphere	Bradyrhizobium	1
Hill	Endosphere	Chryseobacterium	1
Hill	Endosphere	Clostridium_sensu_stricto_13	1
Hill	Endosphere	Cutibacterium	1
Hill	Endosphere	Dyadobacter	1
Hill	Endosphere	Galbitalea	1
Hill	Endosphere	Kocuria	1
Hill	Endosphere	Mycobacterium	1
Hill	Endosphere	Nubsella	1
Hill	Endosphere	Parafrigoribacterium	1
Hill	Endosphere	Pseudomonas	1
Hill	Endosphere	Ralstonia	1
Hill	Endosphere	Rhodopseudomonas	1
Hill	Endosphere	Tardiphaga	1
Hill	Endosphere	Umezawaea	1
Hill	Endosphere	Undibacterium	1

Hill	Endosphere	Variovorax	1
Hill	Rhizosphere	Nakamurella	10
Hill	Rhizosphere	Candidatus_Udaeobacter	9
Hill	Rhizosphere	Gemmatimonas	9
Hill	Rhizosphere	Nocardioides	8
Hill	Rhizosphere	CL500-29_marine_group	7
Hill	Rhizosphere	Sphingomonas	7
Hill	Rhizosphere	Acidiphilium	5
Hill	Rhizosphere	Jatrophihabitans	5
Hill	Rhizosphere	Parafrigoribacterium	5
Hill	Rhizosphere	Oryzihumus	4
Hill	Rhizosphere	Pseudonocardia	4
Hill	Rhizosphere	Bryobacter	3
Hill	Rhizosphere	Candidatus_Nostocoida	3
Hill	Rhizosphere	Devosia	3
Hill	Rhizosphere	Rhizobacter	3
Hill	Rhizosphere	Tundrisphaera	3
Hill	Rhizosphere	Bradyrhizobium	2
Hill	Rhizosphere	Candidatus_Xiphinematobacter	2
Hill	Rhizosphere	Cryobacterium	2
Hill	Rhizosphere	Gaiella	2
Hill	Rhizosphere	Janibacter	2
Hill	Rhizosphere	Kineosporia	2
Hill	Rhizosphere	Novosphingobium	2
Hill	Rhizosphere	Pedobacter	2
Hill	Rhizosphere	Polaromonas	2
Hill	Rhizosphere	Rhodanobacter	2
Hill	Rhizosphere	Rhodococcus	2
Hill	Rhizosphere	Acidipila	1
Hill	Rhizosphere	Actinoplanes	1

Hill	Rhizosphere	Aeromicrobium	1
Hill	Rhizosphere	Allorhizobium-Neorhizobium-Pararhizobium-Rhizobium	1
Hill	Rhizosphere	Angustibacter	1
Hill	Rhizosphere	CM1G08	1
Hill	Rhizosphere	Candidatus_Solibacter	1
Hill	Rhizosphere	Cellulomonas	1
Hill	Rhizosphere	Chthonomonas	1
Hill	Rhizosphere	Clostridium_sensu_stricto_13	1
Hill	Rhizosphere	Conexibacter	1
Hill	Rhizosphere	Dactylosporangium	1
Hill	Rhizosphere	Duganella	1
Hill	Rhizosphere	Ferruginibacter	1
Hill	Rhizosphere	Galbitalea	1
Hill	Rhizosphere	Gemmata	1
Hill	Rhizosphere	Granulicella	1
Hill	Rhizosphere	Haliangium	1
Hill	Rhizosphere	Herbaspirillum	1
Hill	Rhizosphere	JGI_0001001-H03	1
Hill	Rhizosphere	Knoellia	1
Hill	Rhizosphere	Legionella	1
Hill	Rhizosphere	Lysinimonas	1
Hill	Rhizosphere	Lysobacter	1
Hill	Rhizosphere	Mesorhizobium	1
Hill	Rhizosphere	Modestobacter	1
Hill	Rhizosphere	Mycobacterium	1
Hill	Rhizosphere	Nitrospira	1
Hill	Rhizosphere	Phycicoccus	1
Hill	Rhizosphere	Plot4-2H12	1
Hill	Rhizosphere	Singulisphaera	1

Hill	Rhizosphere	Subgroup_10	1

Table S4. Weighted nearest sequenced taxon index (NSTI) values obtained from PICRUSt2 for each sample.

Sample ID	Weighted NSTI
Fran-17	0.0164
Fran-1	0.0244
Fran-2	0.0289
Fran-3	0.0332
Fran-8	0.0408
Fran-18	0.0430
Fran-16	0.0592
Fran-14	0.0658
Fran-11	0.0688
Fran-15	0.0797
Fran-13	0.0852
Fran-10	0.0856
Fran-B	0.0902
Fran-F	0.1525
Fran-L	0.1852
Fran-A	0.1944
Fran-7	0.2042
Fran-N	0.2069
Fran-Q	0.2085
Fran-P	0.2089
Fran-9	0.2100
Fran-H	0.2256
Fran-C	0.2293
Fran-O	0.2308
Fran-G	0.2488
Fran-M	0.2783
Fran-I	0.3343
Fran-K	0.3412
Fran-J	0.4413
Fran-12	0.7133

Table S5. Curated subset of 669 KEGG Orthologs (KOs) included in the predicted functional analysis

Each KEGG Ortholog (KO) is shown once. Associated pathways were collapsed into a single field to avoid duplicated KO entries. Gene names correspond to the KEGG annotation associated with each KO.

KO	Gene name	Associated pathways

K00001	E1.1.1.1, adh; alcohol dehydrogenase [EC:1.1.1.1]	Chloroalkane and chloroalkene degradation; Degradation of aromatic compounds; Fatty acid degradation; Metabolism of xenobiotics by cytochrome P450; Naphthalene degradation
K00002	AKR1A1, adh; alcohol dehydrogenase (NADP+) [EC:1.1.1.2]	Caprolactam degradation; Degradation of aromatic compounds
K00004	budC	Growth-Promoting volatiles
K00022	HADH; 3-hydroxyacyl-CoA dehydrogenase [EC:1.1.1.35]	Caprolactam degradation
K00024	mdh; malate dehydrogenase [EC:1.1.1.37]	Citrate cycle (TCA cycle); Pyruvate metabolism
K00029	maeB; malate dehydrogenase (oxaloacetate-decarboxylating)(NADP+) [EC:1.1.1.40]	Pyruvate metabolism
K00031	IDH1, IDH2, icd, IDP; isocitrate dehydrogenase [EC:1.1.1.42]	Citrate cycle (TCA cycle)
K00055	E1.1.1.90; aryl-alcohol dehydrogenase [EC:1.1.1.90]	Degradation of aromatic compounds; Toluene degradation; Xylene degradation
K00059	fabG, OAR1; 3-oxoacyl-[acyl-carrier protein] reductase [EC:1.1.1.100]	Fatty acid biosynthesis
K00074	paaH, hbd, fadB, mmgB; 3-hydroxybutyryl-CoA dehydrogenase [EC:1.1.1.157]	Benzoate degradation
K00078	DHDH; dihydrodiol dehydrogenase / D-xylose 1-dehydrogenase (NADP) [EC:1.3.1.20 1.1.1.179]	Metabolism of xenobiotics by cytochrome P450
K00079	CBR1; carbonyl reductase 1 [EC:1.1.1.184 1.1.1.189 1.1.1.197]	Metabolism of xenobiotics by cytochrome P450
K00081	CBR2; carbonyl reductase 2 [EC:1.1.1.184]	Metabolism of xenobiotics by cytochrome P450
K00084	CBR3; carbonyl reductase 3 [EC:1.1.1.184]	Metabolism of xenobiotics by cytochrome P450
K00090	ghrB; glyoxylate/hydroxypyruvate/2-ketogluconate reductase [EC:1.1.1.79 1.1.1.81 1.1.1.215]	Pyruvate metabolism
K00101	lldD; L-lactate dehydrogenase (cytochrome) [EC:1.1.2.3]	Pyruvate metabolism
K00102	LDHD, dld; D-lactate dehydrogenase (cytochrome) [EC:1.1.2.4]	Pyruvate metabolism
K00108	betA	Stress tolerance/ osmolytes
K00114	exaA; alcohol dehydrogenase (cytochrome c) [EC:1.1.2.8]	Chloroalkane and chloroalkene degradation
K00116	mqo; malate dehydrogenase (quinone) [EC:1.1.5.4]	Citrate cycle (TCA cycle); Pyruvate metabolism
K00117	gcd	Phosphorus

K00121	frmA, ADH5, adhC; S-(hydroxymethyl)glutathione dehydrogenase / alcohol dehydrogenase [EC:1.1.1.284 1.1.1.1]	Chloroalkane and chloroalkene degradation; Degradation of aromatic compounds; Fatty acid degradation; Glycolysis / Gluconeogenesis; Metabolism of xenobiotics by cytochrome P450; Naphthalene degradation; Pyruvate metabolism
K00128	ALDH; aldehyde dehydrogenase (NAD+) [EC:1.2.1.3]	Chloroalkane and chloroalkene degradation; Fatty acid degradation
K00129	E1.2.1.5; aldehyde dehydrogenase (NAD(P)+) [EC:1.2.1.5]	Metabolism of xenobiotics by cytochrome P450
K00130	betB	Stress tolerance/ osmolytes
K00131	gapN; glyceraldehyde-3-phosphate dehydrogenase (NADP+) [EC:1.2.1.9]	Glycolysis / Gluconeogenesis
K00134	GAPDH, gapA; glyceraldehyde 3-phosphate dehydrogenase (phosphorylating) [EC:1.2.1.12]	Glycolysis / Gluconeogenesis
K00138	aldB; aldehyde dehydrogenase [EC:1.2.1.-]	Glycolysis / Gluconeogenesis; Pyruvate metabolism
K00141	xylC; benzaldehyde dehydrogenase (NAD) [EC:1.2.1.28]	Aminobenzoate degradation; Degradation of aromatic compounds; Toluene degradation; Xylene degradation
K00148	fdhA; glutathione-independent formaldehyde dehydrogenase [EC:1.2.1.46]	Chloroalkane and chloroalkene degradation
K00150	gap2, gapB; glyceraldehyde-3-phosphate dehydrogenase (NAD(P)+) (phosphorylating) [EC:1.2.1.59]	Glycolysis / Gluconeogenesis
K00151	hpaE, hpcC; 5-carboxymethyl-2-hydroxymuconic-semialdehyde dehydrogenase [EC:1.2.1.60]	Degradation of aromatic compounds
K00152	nahF; salicylaldehyde dehydrogenase [EC:1.2.1.65]	Degradation of aromatic compounds; Naphthalene degradation
K00156	poxB; pyruvate dehydrogenase (quinone) [EC:1.2.5.1]	Pyruvate metabolism
K00161	PDHA, pdhA; pyruvate dehydrogenase E1 component subunit alpha [EC:1.2.4.1]	Citrate cycle (TCA cycle); Glycolysis / Gluconeogenesis; Pyruvate metabolism
K00162	PDHB, pdhB; pyruvate dehydrogenase E1 component subunit beta [EC:1.2.4.1]	Citrate cycle (TCA cycle); Glycolysis / Gluconeogenesis; Pyruvate metabolism
K00163	aceE; pyruvate dehydrogenase E1 component [EC:1.2.4.1]	Citrate cycle (TCA cycle); Glycolysis / Gluconeogenesis; Pyruvate metabolism
K00164	OGDH, sucA; 2-oxoglutarate dehydrogenase E1 component [EC:1.2.4.2]	Citrate cycle (TCA cycle)
K00174	korA, oorA, oforA; 2-oxoglutarate/2-oxoacid ferredoxin oxidoreductase subunit alpha [EC:1.2.7.3 1.2.7.11]	Citrate cycle (TCA cycle); Glycolysis / Gluconeogenesis; Pyruvate metabolism

K00175	korB, oorB, oforB; 2-oxoglutarate/2-oxoacid ferredoxin oxidoreductase subunit beta [EC:1.2.7.3 1.2.7.11]	Citrate cycle (TCA cycle); Glycolysis / Gluconeogenesis; Pyruvate metabolism
K00208	fabI; enoyl-[acyl-carrier protein] reductase I [EC:1.3.1.9 1.3.1.10]	Fatty acid biosynthesis
K00209	fabV, ter; enoyl-[acyl-carrier protein] reductase / trans-2-enoyl-CoA reductase (NAD+) [EC:1.3.1.9 1.3.1.44]	Fatty acid biosynthesis
K00212	AKR1C1; 20alpha/3alpha-hydroxysteroid dehydrogenase / dihydrodiol dehydrogenase [EC:1.1.1.149 1.1.1.357 1.3.1.20]	Metabolism of xenobiotics by cytochrome P450
K00216	entA	Iron acquisition (siderophores)
K00217	E1.3.1.32; maleylacetate reductase [EC:1.3.1.32]	Benzoate degradation; Chlorocyclohexane and chlorobenzene degradation; Degradation of aromatic compounds; Fluorobenzoate degradation; Toluene degradation
K00232	ACOX1_3, POX1; acyl-CoA oxidase [EC:1.3.3.6]	Fatty acid degradation
K00239	sdhA, frdA; succinate dehydrogenase flavoprotein subunit [EC:1.3.5.1]	Citrate cycle (TCA cycle)
K00240	sdhB, frdB; succinate dehydrogenase iron-sulfur subunit [EC:1.3.5.1]	Citrate cycle (TCA cycle)
K00241	sdhC, frdC; succinate dehydrogenase cytochrome b subunit	Citrate cycle (TCA cycle)
K00242	sdhD, frdD; succinate dehydrogenase membrane anchor subunit	Citrate cycle (TCA cycle)
K00248	ACADS, bcd; butyryl-CoA dehydrogenase [EC:1.3.8.1]	Fatty acid degradation
K00249	ACADM, acd; acyl-CoA dehydrogenase [EC:1.3.8.7]	Fatty acid degradation
K00252	GCDH, gcdH; glutaryl-CoA dehydrogenase [EC:1.3.8.6]	Benzoate degradation; Fatty acid degradation
K00255	ACADL; long-chain-acyl-CoA dehydrogenase [EC:1.3.8.8]	Fatty acid degradation
K00380	cysJ	Sulfur metabolism
K00381	cysI	Sulfur metabolism
K00382	DLD, lpd, pdhD; dihydrolipoyl dehydrogenase [EC:1.8.1.4]	Citrate cycle (TCA cycle); Glycolysis / Gluconeogenesis; Pyruvate metabolism
K00390	cysH	Sulfur metabolism

K00446	dmpB, xylE; catechol 2,3-dioxygenase [EC:1.13.11.2]	Benzoate degradation; Chlorocyclohexane and chlorobenzene degradation; Degradation of aromatic compounds; Xylene degradation
K00448	pcaG; protocatechuate 3,4-dioxygenase, alpha subunit [EC:1.13.11.3]	Benzoate degradation; Degradation of aromatic compounds; Polycyclic aromatic hydrocarbon degradation
K00449	pcaH; protocatechuate 3,4-dioxygenase, beta subunit [EC:1.13.11.3]	Benzoate degradation; Degradation of aromatic compounds; Polycyclic aromatic hydrocarbon degradation
K00455	hpaD, hpcB; 3,4-dihydroxyphenylacetate 2,3-dioxygenase [EC:1.13.11.15]	Degradation of aromatic compounds
K00462	bphC; biphenyl-2,3-diol 1,2-dioxygenase [EC:1.13.11.39]	Chlorocyclohexane and chlorobenzene degradation; Degradation of aromatic compounds
K00466	iaaM	Phytohormones
K00480	E1.14.13.1; salicylate hydroxylase [EC:1.14.13.1]	Degradation of aromatic compounds; Naphthalene degradation; Polycyclic aromatic hydrocarbon degradation
K00481	pobA; p-hydroxybenzoate 3-monooxygenase [EC:1.14.13.2]	Benzoate degradation; Degradation of aromatic compounds
K00483	hpaB; 4-hydroxyphenylacetate 3-monooxygenase [EC:1.14.14.9]	Degradation of aromatic compounds
K00484	hpaC; flavin reductase (NADH) [EC:1.5.1.36]	Degradation of aromatic compounds
K00487	CYP73A; trans-cinnamate 4-monooxygenase [EC:1.14.14.91]	Degradation of aromatic compounds
K00496	alkB1_2, alkM; alkane 1-monooxygenase [EC:1.14.15.3]	Caprolactam degradation; Fatty acid degradation
K00529	hcaD; 3-phenylpropionate/trans-cinnamate dioxygenase ferredoxin reductase component [EC:1.18.1.3]	Benzoate degradation; Degradation of aromatic compounds; Fatty acid degradation
K00531	anfG; nitrogenase delta subunit [EC:1.18.6.1]; anfG	Chloroalkane and chloroalkene degradation; Nitrogen metabolism
K00604	MTFMT, fmt; methionyl-tRNA formyltransferase [EC:2.1.2.9]	Aminoacyl-tRNA biosynthesis
K00625	pta; phosphate acetyltransferase [EC:2.3.1.8]	Pyruvate metabolism
K00626	ACAT, atoB; acetyl-CoA C-acetyltransferase [EC:2.3.1.9]; E2.3.1.9, atoB; acetyl-CoA C-acetyltransferase [EC:2.3.1.9]	Benzoate degradation; Fatty acid degradation; Pyruvate metabolism
K00627	DLAT, aceF, pdhC; pyruvate dehydrogenase E2 component (dihydrolipoyllysine-residue acetyltransferase) [EC:2.3.1.12]	Citrate cycle (TCA cycle); Glycolysis / Gluconeogenesis; Pyruvate metabolism

K00632	fadA, fadI; acetyl-CoA acyltransferase [EC:2.3.1.16]	Benzoate degradation; Fatty acid degradation
K00645	fabD, MCAT, MCT1; [acyl-carrier-protein] S-malonyltransferase [EC:2.3.1.39]	Fatty acid biosynthesis
K00647	fabB; 3-oxoacyl-[acyl-carrier-protein] synthase I [EC:2.3.1.41]	Fatty acid biosynthesis
K00648	fabH; 3-oxoacyl-[acyl-carrier-protein] synthase III [EC:2.3.1.180]	Fatty acid biosynthesis
K00656	E2.3.1.54, pflD; formate C-acetyltransferase [EC:2.3.1.54]	Pyruvate metabolism
K00658	DLST, sucB; 2-oxoglutarate dehydrogenase E2 component (dihydrolipoamide succinyltransferase) [EC:2.3.1.61]	Citrate cycle (TCA cycle)
K00697	otsA	Stress tolerance/ osmolytes
K00699	UGT; glucuronosyltransferase [EC:2.4.1.17]	Metabolism of xenobiotics by cytochrome P450
K00791	miaA	Phytohormones
K00799	GST, gst; glutathione S-transferase [EC:2.5.1.18]	Metabolism of xenobiotics by cytochrome P450
K00836	ectB	Stress tolerance/ osmolytes
K00845	glk; glucokinase [EC:2.7.1.2]	Glycolysis / Gluconeogenesis
K00850	pfkA, PFK; 6-phosphofructokinase 1 [EC:2.7.1.11]	Glycolysis / Gluconeogenesis
K00860	cysC	Sulfur metabolism
K00873	PK, pyk; pyruvate kinase [EC:2.7.1.40]	Glycolysis / Gluconeogenesis; Pyruvate metabolism
K00886	ppgK; polyphosphate glucokinase [EC:2.7.1.63]	Glycolysis / Gluconeogenesis
K00925	ackA; acetate kinase [EC:2.7.2.1]	Pyruvate metabolism
K00927	PGK, pgk; phosphoglycerate kinase [EC:2.7.2.3]	Glycolysis / Gluconeogenesis
K00937	ppk	Phosphorus
K00955	cysN	Sulfur metabolism
K00957	cysD	Sulfur metabolism
K00958	sat	Sulfur metabolism
K00960	rpo13; DNA-directed RNA polymerase subunit 13 [EC:2.7.7.6]	RNA polymerase
K01006	ppdK; pyruvate, orthophosphate dikinase [EC:2.7.9.1]	Glycolysis / Gluconeogenesis; Pyruvate metabolism
K01007	pps, ppsA; pyruvate, water dikinase [EC:2.7.9.2]	Glycolysis / Gluconeogenesis; Pyruvate metabolism

K01026	pct; propionate CoA-transferase [EC:2.8.3.1]	Pyruvate metabolism
K01031	pcaI; 3-oxoadipate CoA-transferase, alpha subunit [EC:2.8.3.6]	Benzoate degradation
K01032	pcaJ; 3-oxoadipate CoA-transferase, beta subunit [EC:2.8.3.6]	Benzoate degradation
K01034	atoD; acetate CoA/acetoacetate CoA-transferase alpha subunit [EC:2.8.3.8 2.8.3.9]	Aminobenzoate degradation
K01035	atoA; acetate CoA/acetoacetate CoA-transferase beta subunit [EC:2.8.3.8 2.8.3.9]	Aminobenzoate degradation
K01042	selA; L-seryl-tRNA(Ser) seleniumtransferase [EC:2.9.1.1]	Aminoacyl-tRNA biosynthesis
K01053	gnl, RGN; gluconolactonase [EC:3.1.1.17]; E3.1.1.17, gnl, RGN; gluconolactonase [EC:3.1.1.17]	Caprolactam degradation; Degradation of aromatic compounds
K01055	pcaD; 3-oxoadipate enol-lactonase [EC:3.1.1.24]	Benzoate degradation; Degradation of aromatic compounds
K01061	clcD; carboxymethylenebutenolidase [EC:3.1.1.45]; E3.1.1.45; carboxymethylenebutenolidase [EC:3.1.1.45]	Chlorocyclohexane and chlorobenzene degradation; Fluorobenzoate degradation; Toluene degradation
K01069	gloB, gloC, HAGH; hydroxyacylglutathione hydrolase [EC:3.1.2.6]	Pyruvate metabolism
K01071	MCH; medium-chain acyl-[acyl-carrier-protein] hydrolase [EC:3.1.2.21]	Fatty acid biosynthesis
K01075	E3.1.2.23; 4-hydroxybenzoyl-CoA thioesterase [EC:3.1.2.23]	Benzoate degradation
K01077	phoA	Phosphorus
K01087	otsB	Stress tolerance/ osmolytes
K01093	appA	Phosphorus
K01101	E3.1.3.41; 4-nitrophenyl phosphatase [EC:3.1.3.41]	Aminobenzoate degradation
K01113	phoD	Phosphorus
K01141	sbcB, exoI; exodeoxyribonuclease I [EC:3.1.11.1]	Mismatch repair
K01142	xthA, xth; exodeoxyribonuclease III [EC:3.1.11.2]	Base excision repair
K01151	nfo; deoxyribonuclease IV [EC:3.1.21.2]	Base excision repair
K01159	ruvC; crossover junction endodeoxyribonuclease RuvC [EC:3.1.21.10]	Homologous recombination
K01183	chiA	Biocontrol

K01246	tag; DNA-3-methyladenine glycosylase I [EC:3.2.2.20]	Base excision repair
K01247	alkA; DNA-3-methyladenine glycosylase II [EC:3.2.2.21]	Base excision repair
K01252	entB	Iron acquisition (siderophores)
K01253	EPHX1; microsomal epoxide hydrolase [EC:3.3.2.9]	Metabolism of xenobiotics by cytochrome P450
K01399	lasB	Biocontrol
K01426	E3.5.1.4, amiE; amidase [EC:3.5.1.4]	Aminobenzoate degradation; Phytohormones
K01453	nylB; 6-aminohexanoate-oligomer exohydrolase [EC:3.5.1.46]	Caprolactam degradation
K01471	nylA; 6-aminohexanoate-cyclic-dimer hydrolase [EC:3.5.2.12]	Caprolactam degradation
K01501	E3.5.5.1; nitrilase [EC:3.5.5.1]	Aminobenzoate degradation; Phytohormones
K01505	acdS	Ethylene Modulation
K01512	acyP; acylphosphatase [EC:3.6.1.7]	Aminobenzoate degradation; Pyruvate metabolism
K01524	ppx	Phosphorus
K01560	E3.8.1.2; 2-haloacid dehalogenase [EC:3.8.1.2]	Chloroalkane and chloroalkene degradation; Chlorocyclohexane and chlorobenzene degradation
K01561	dehH; haloacetate dehalogenase [EC:3.8.1.3]	Chloroalkane and chloroalkene degradation; Chlorocyclohexane and chlorobenzene degradation
K01563	dhaA; haloalkane dehalogenase [EC:3.8.1.5]	Chloroalkane and chloroalkene degradation; Chlorocyclohexane and chlorobenzene degradation
K01575	budA/alsD	Growth-Promoting volatiles
K01576	mdlC; benzoylformate decarboxylase [EC:4.1.1.7]	Aminobenzoate degradation
K01580	gadB	Quorum sensing
K01595	ppc; phosphoenolpyruvate carboxylase [EC:4.1.1.31]	Pyruvate metabolism
K01596	E4.1.1.32, pckA, PCK; phosphoenolpyruvate carboxykinase (GTP) [EC:4.1.1.32]	Citrate cycle (TCA cycle); Glycolysis / Gluconeogenesis; Pyruvate metabolism
K01607	pcaC; 4-carboxymuconolactone decarboxylase [EC:4.1.1.44]	Benzoate degradation; Degradation of aromatic compounds
K01610	pckA; phosphoenolpyruvate carboxykinase (ATP) [EC:4.1.1.49]	Citrate cycle (TCA cycle); Glycolysis / Gluconeogenesis; Pyruvate metabolism
K01612	bsdC; vanillate/4-hydroxybenzoate decarboxylase subunit C [EC:4.1.1.- 4.1.1.61]	Degradation of aromatic compounds

K01615	gcdA; glutaconyl-CoA decarboxylase subunit alpha [EC:7.2.4.5]	Benzoate degradation
K01617	dmpH, xylI, nahK; 2-oxo-3-hexenedioate decarboxylase [EC:4.1.1.77]	Benzoate degradation; Degradation of aromatic compounds; Xylene degradation
K01623	ALDO; fructose-bisphosphate aldolase, class I [EC:4.1.2.13]	Glycolysis / Gluconeogenesis
K01624	FBA, fbaA; fructose-bisphosphate aldolase, class II [EC:4.1.2.13]	Glycolysis / Gluconeogenesis
K01652	ilvB/alsS	Growth-Promoting volatiles
K01666	mhpE; 4-hydroxy 2-oxovalerate aldolase [EC:4.1.3.39]	Benzoate degradation; Degradation of aromatic compounds; Xylene degradation
K01670	nmsA; naphthyl-2-methylsuccinate synthase alpha subunit [EC:4.1.99.-]	Degradation of aromatic compounds; Naphthalene degradation
K01692	paaF, echA; enoyl-CoA hydratase [EC:4.2.1.17]	Aminobenzoate degradation; Benzoate degradation; Caprolactam degradation; Fatty acid degradation
K01716	fabA; 3-hydroxyacyl-[acyl-carrier protein] dehydratase / trans-2-decenoyl-[acyl-carrier protein] isomerase [EC:4.2.1.59 5.3.3.14]	Fatty acid biosynthesis
K01721	nthA; nitrile hydratase subunit alpha [EC:4.2.1.84]; nthA	Aminobenzoate degradation; Phytohormones; Fluorobenzoate degradation
K01738	cysK	Sulfur metabolism
K01759	GLO1, gloA; lactoylglutathione lyase [EC:4.4.1.5]	Pyruvate metabolism
K01781	mdlA; mandelate racemase [EC:5.1.2.2]	Aminobenzoate degradation
K01782	fadJ; 3-hydroxyacyl-CoA dehydrogenase / enoyl-CoA hydratase / 3-hydroxybutyryl-CoA epimerase [EC:1.1.1.35 4.2.1.17 5.1.2.3]	Benzoate degradation; Caprolactam degradation; Fatty acid degradation
K01785	galM, GALM; aldose 1-epimerase [EC:5.1.3.3]	Glycolysis / Gluconeogenesis
K01792	E5.1.3.15; glucose-6-phosphate 1-epimerase [EC:5.1.3.15]	Glycolysis / Gluconeogenesis
K01803	TPI, tpiA; triosephosphate isomerase (TIM) [EC:5.3.1.1]	Glycolysis / Gluconeogenesis
K01810	GPI, pgi; glucose-6-phosphate isomerase [EC:5.3.1.9]	Glycolysis / Gluconeogenesis
K01821	praC, xylH; 4-oxalocrotonate tautomerase [EC:5.3.2.6]	Benzoate degradation; Degradation of aromatic compounds; Xylene degradation
K01825	fadB; 3-hydroxyacyl-CoA dehydrogenase / enoyl-CoA hydratase / 3-hydroxybutyryl-CoA epimerase / enoyl-CoA isomerase [EC:1.1.1.35 4.2.1.17 5.1.2.3 5.3.3.8]	Benzoate degradation; Caprolactam degradation; Fatty acid degradation

K01826	hpaF, hpcD; 5-carboxymethyl-2-hydroxymuconate isomerase [EC:5.3.3.10]	Degradation of aromatic compounds
K01856	catB; muconate cycloisomerase [EC:5.5.1.1]	Benzoate degradation; Chlorocyclohexane and chlorobenzene degradation; Degradation of aromatic compounds; Fluorobenzoate degradation; Toluene degradation
K01857	pcaB; 3-carboxy-cis,cis-muconate cycloisomerase [EC:5.5.1.2]	Benzoate degradation; Degradation of aromatic compounds
K01860	E5.5.1.7; chloromuconate cycloisomerase [EC:5.5.1.7]	Fluorobenzoate degradation; Toluene degradation
K01865	hab; (hydroxyamino)benzene mutase [EC:5.4.4.1]	Aminobenzoate degradation
K01866	YARS, tyrS; tyrosyl-tRNA synthetase [EC:6.1.1.1]	Aminoacyl-tRNA biosynthesis
K01867	WARS, trpS; tryptophanyl-tRNA synthetase [EC:6.1.1.2]	Aminoacyl-tRNA biosynthesis
K01868	TARS, thrS; threonyl-tRNA synthetase [EC:6.1.1.3]	Aminoacyl-tRNA biosynthesis
K01869	LARS, leuS; leucyl-tRNA synthetase [EC:6.1.1.4]	Aminoacyl-tRNA biosynthesis
K01870	IARS, ileS; isoleucyl-tRNA synthetase [EC:6.1.1.5]	Aminoacyl-tRNA biosynthesis
K01872	AARS, alaS; alanyl-tRNA synthetase [EC:6.1.1.7]	Aminoacyl-tRNA biosynthesis
K01873	VARS, valS; valyl-tRNA synthetase [EC:6.1.1.9]	Aminoacyl-tRNA biosynthesis
K01874	MARS, metG; methionyl-tRNA synthetase [EC:6.1.1.10]	Aminoacyl-tRNA biosynthesis
K01875	SARS, serS; seryl-tRNA synthetase [EC:6.1.1.11]	Aminoacyl-tRNA biosynthesis
K01876	DARS2, aspS; aspartyl-tRNA synthetase [EC:6.1.1.12]	Aminoacyl-tRNA biosynthesis
K01878	glyQ; glycyl-tRNA synthetase alpha chain [EC:6.1.1.14]	Aminoacyl-tRNA biosynthesis
K01879	glyS; glycyl-tRNA synthetase beta chain [EC:6.1.1.14]	Aminoacyl-tRNA biosynthesis
K01880	GARS, glyS1; glycyl-tRNA synthetase [EC:6.1.1.14]	Aminoacyl-tRNA biosynthesis
K01881	PARS, proS; prolyl-tRNA synthetase [EC:6.1.1.15]	Aminoacyl-tRNA biosynthesis
K01883	CARS, cysS; cysteinyl-tRNA synthetase [EC:6.1.1.16]	Aminoacyl-tRNA biosynthesis
K01884	cysS1; cysteinyl-tRNA synthetase, unknown class [EC:6.1.1.16]	Aminoacyl-tRNA biosynthesis

K01885	EARS, gltX; glutamyl-tRNA synthetase [EC:6.1.1.17]	Aminoacyl-tRNA biosynthesis
K01886	QARS, glnS; glutaminyl-tRNA synthetase [EC:6.1.1.18]	Aminoacyl-tRNA biosynthesis
K01887	RARS, argS; arginyl-tRNA synthetase [EC:6.1.1.19]	Aminoacyl-tRNA biosynthesis
K01889	FARSA, pheS; phenylalanyl-tRNA synthetase alpha chain [EC:6.1.1.20]	Aminoacyl-tRNA biosynthesis
K01890	FARSB, pheT; phenylalanyl-tRNA synthetase beta chain [EC:6.1.1.20]	Aminoacyl-tRNA biosynthesis
K01892	HARS, hisS; histidyl-tRNA synthetase [EC:6.1.1.21]	Aminoacyl-tRNA biosynthesis
K01893	NARS, asnS; asparaginyl-tRNA synthetase [EC:6.1.1.22]	Aminoacyl-tRNA biosynthesis
K01897	ACSL, fadD; long-chain-fatty-acid---CoA ligase [EC:6.2.1.3]	Fatty acid biosynthesis; Fatty acid degradation
K01909	mbtM; long-chain-fatty-acid--[acyl-carrier-protein] ligase [EC:6.2.1.20]	Fatty acid degradation
K01961	accC; acetyl-CoA carboxylase, biotin carboxylase subunit [EC:6.4.1.2 6.3.4.14]	Fatty acid biosynthesis
K01962	accA; acetyl-CoA carboxylase carboxyl transferase subunit alpha [EC:6.4.1.2 2.1.3.15]	Fatty acid biosynthesis
K01963	accD; acetyl-CoA carboxylase carboxyl transferase subunit beta [EC:6.4.1.2 2.1.3.15]	Fatty acid biosynthesis
K01971	ligD; bifunctional non-homologous end joining protein LigD [EC:6.5.1.1]	Non-homologous end-joining
K01972	E6.5.1.2, ligA, ligB; DNA ligase (NAD+) [EC:6.5.1.2]	Base excision repair; DNA replication; Mismatch repair; Nucleotide excision repair
K02016	ABC Fe3+-sid.	Iron acquisition (siderophores)
K02036	pstB	Phosphorus
K02037	pstC	Phosphorus
K02038	pstA	Phosphorus
K02040	pstS	Phosphorus
K02160	accB, bccP; acetyl-CoA carboxylase biotin carboxyl carrier protein	Fatty acid biosynthesis
K02314	dnaB; replicative DNA helicase [EC:5.6.2.3]	DNA replication
K02316	dnaG; DNA primase [EC:2.7.7.101]	DNA replication

K02317	dnaT; DNA replication protein DnaT	Homologous recombination
K02335	polA; DNA polymerase I [EC:2.7.7.7]	Base excision repair; DNA replication; Homologous recombination; Nucleotide excision repair
K02337	dnaE; DNA polymerase III subunit alpha [EC:2.7.7.7]	DNA replication; Homologous recombination; Mismatch repair
K02338	dnaN; DNA polymerase III subunit beta [EC:2.7.7.7]	DNA replication; Homologous recombination; Mismatch repair
K02339	holC; DNA polymerase III subunit chi [EC:2.7.7.7]	DNA replication; Homologous recombination; Mismatch repair
K02340	holA; DNA polymerase III subunit delta [EC:2.7.7.7]	DNA replication; Homologous recombination; Mismatch repair
K02341	holB; DNA polymerase III subunit delta' [EC:2.7.7.7]	DNA replication; Homologous recombination; Mismatch repair
K02342	dnaQ; DNA polymerase III subunit epsilon [EC:2.7.7.7]	DNA replication; Homologous recombination; Mismatch repair
K02343	dnaX; DNA polymerase III subunit gamma/tau [EC:2.7.7.7]	DNA replication; Homologous recombination; Mismatch repair
K02344	holD; DNA polymerase III subunit psi [EC:2.7.7.7]	DNA replication; Homologous recombination; Mismatch repair
K02345	holE; DNA polymerase III subunit theta [EC:2.7.7.7]	DNA replication; Homologous recombination; Mismatch repair
K02361	entC	Iron acquisition (siderophores)
K02363	entE	Iron acquisition (siderophores)
K02364	entD	Iron acquisition (siderophores)
K02371	fabK; enoyl-[acyl-carrier protein] reductase II [EC:1.3.1.9]	Fatty acid biosynthesis
K02372	fabZ; 3-hydroxyacyl-[acyl-carrier-protein] dehydratase [EC:4.2.1.59]	Fatty acid biosynthesis
K02402	flhC	Competition/colonization
K02403	flhD	Competition/colonization
K02406	fliC	Competition/colonization
K02433	gatA, QRSL1; aspartyl-tRNA(Asn)/glutamyl-tRNA(Gln) amidotransferase subunit A [EC:6.3.5.6 6.3.5.7]	Aminoacyl-tRNA biosynthesis
K02434	gatB, PET112; aspartyl-tRNA(Asn)/glutamyl-tRNA(Gln) amidotransferase subunit B [EC:6.3.5.6 6.3.5.7]	Aminoacyl-tRNA biosynthesis

K02435	gatC, GATC; aspartyl-tRNA(Asn)/glutamyl-tRNA(Gln) amidotransferase subunit C [EC:6.3.5.6 6.3.5.7]	Aminoacyl-tRNA biosynthesis
K02554	mhpD; 2-keto-4-pentenoate hydratase [EC:4.2.1.80]	Benzoate degradation; Degradation of aromatic compounds; Xylene degradation
K02556	motA	Competition/colonization
K02585	nifB	Nitrogen metabolism
K02586	nifD; nitrogenase molybdenum-iron protein alpha chain [EC:1.18.6.1]; nifD	Chloroalkane and chloroalkene degradation; Nitrogen metabolism
K02587	nifE	Nitrogen metabolism
K02588	nifH; nitrogenase iron protein NifH; nifH	Chloroalkane and chloroalkene degradation; Nitrogen metabolism
K02591	nifK; nitrogenase molybdenum-iron protein beta chain [EC:1.18.6.1]; nifK	Chloroalkane and chloroalkene degradation; Nitrogen metabolism
K02592	nifN	Nitrogen metabolism
K02686	priB; primosomal replication protein N	Homologous recombination
K02851	wecA	Competition/colonization
K02864	RP-L10, MRPL10, rplJ; large subunit ribosomal protein L10	Ribosome
K02867	RP-L11, MRPL11, rplK; large subunit ribosomal protein L11	Ribosome
K02886	RP-L2, MRPL2, RML2, rplB; large subunit ribosomal protein L2	Ribosome
K02906	RP-L3, MRPL3, rplC; large subunit ribosomal protein L3	Ribosome
K02926	RP-L4, MRPL4, rplD; large subunit ribosomal protein L4	Ribosome
K02931	RP-L5, MRPL5, rplE; large subunit ribosomal protein L5	Ribosome
K02933	RP-L6, MRPL6, rplF; large subunit ribosomal protein L6	Ribosome
K02945	RP-S1, rpsA; small subunit ribosomal protein S1	Ribosome
K02950	RP-S12, MRPS12, rpsL; small subunit ribosomal protein S12	Ribosome
K02967	RP-S2, MRPS2, rpsB; small subunit ribosomal protein S2	Ribosome
K02982	RP-S3, rpsC; small subunit ribosomal protein S3	Ribosome

K02986	RP-S4, NAM9, rpsD; small subunit ribosomal protein S4	Ribosome
K02988	RP-S5, MRPS5, rpsE; small subunit ribosomal protein S5	Ribosome
K02992	RP-S7, MRPS7, rpsG; small subunit ribosomal protein S7	Ribosome
K02994	RP-S8, MRPS8, rpsH; small subunit ribosomal protein S8	Ribosome
K03040	rpoA; DNA-directed RNA polymerase subunit alpha [EC:2.7.7.6]	RNA polymerase
K03043	rpoB; DNA-directed RNA polymerase subunit beta [EC:2.7.7.6]	RNA polymerase
K03046	rpoC; DNA-directed RNA polymerase subunit beta' [EC:2.7.7.6]	RNA polymerase
K03048	rpoE; DNA-directed RNA polymerase subunit delta	RNA polymerase
K03060	rpoZ; DNA-directed RNA polymerase subunit omega [EC:2.7.7.6]	RNA polymerase
K03111	ssb; single-strand DNA-binding protein	DNA replication; Homologous recombination; Mismatch repair
K03119	tauD	Sulfur metabolism
K03186	ubiX, bsdB, PAD1; flavin prenyltransferase [EC:2.5.1.129]	Aminobenzoate degradation; Degradation of aromatic compounds
K03268	todC1, bedC1, tcbAa; benzene/toluene/chlorobenzene dioxygenase subunit alpha [EC:1.14.12.3 1.14.12.11 1.14.12.26]	Benzoate degradation; Degradation of aromatic compounds; Toluene degradation
K03330	gatE; glutamyl-tRNA(Gln) amidotransferase subunit E [EC:6.3.5.7]	Aminoacyl-tRNA biosynthesis
K03379	chnB; cyclohexanone monooxygenase [EC:1.14.13.22]	Caprolactam degradation; Degradation of aromatic compounds
K03380	E1.14.13.7; phenol 2-monooxygenase (NADPH) [EC:1.14.13.7]; E1.14.13.7; phenol 2-monooxygenase [EC:1.14.13.7]	Aminobenzoate degradation; Toluene degradation
K03381	catA; catechol 1,2-dioxygenase [EC:1.13.11.1]	Benzoate degradation; Chlorocyclohexane and chlorobenzene degradation; Degradation of aromatic compounds; Fluorobenzoate degradation; Toluene degradation
K03385	nrfA	Nitrogen metabolism
K03391	pcpB; pentachlorophenol monooxygenase [EC:1.14.13.50]	Chlorocyclohexane and chlorobenzene degradation; Fluorobenzoate degradation

K03407	cheA	Competition/colonization
K03413	cheY	Competition/colonization
K03464	catC; muconolactone D-isomerase [EC:5.3.3.4]	Benzoate degradation; Degradation of aromatic compounds
K03469	rnhA, RNASEH1; ribonuclease HI [EC:3.1.26.4]	DNA replication
K03470	rnhB; ribonuclease HII [EC:3.1.26.4]	DNA replication
K03471	rnhC; ribonuclease HIII [EC:3.1.26.4]	DNA replication
K03550	ruvA; holliday junction DNA helicase RuvA	Homologous recombination
K03551	ruvB; holliday junction DNA helicase RuvB [EC:5.6.2.4]	Homologous recombination
K03553	recA; recombination protein RecA	Homologous recombination
K03555	mutS; DNA mismatch repair protein MutS	Mismatch repair
K03572	mutL; DNA mismatch repair protein MutL	Mismatch repair
K03573	mutH; DNA mismatch repair protein MutH	Mismatch repair
K03575	mutY; A/G-specific adenine glycosylase [EC:3.2.2.31]	Base excision repair
K03581	recD; exodeoxyribonuclease V alpha subunit [EC:3.1.11.5]	Homologous recombination
K03582	recB; exodeoxyribonuclease V beta subunit [EC:3.1.11.5]	Homologous recombination
K03583	recC; exodeoxyribonuclease V gamma subunit [EC:3.1.11.5]	Homologous recombination
K03584	recO; DNA repair protein RecO (recombination protein O)	Homologous recombination
K03601	xseA; exodeoxyribonuclease VII large subunit [EC:3.1.11.6]	Mismatch repair
K03602	xseB; exodeoxyribonuclease VII small subunit [EC:3.1.11.6]	Mismatch repair
K03606	wcaJ	Competition/colonization
K03629	recF; DNA replication and repair protein RecF	Homologous recombination
K03648	UNG, UDG; uracil-DNA glycosylase [EC:3.2.2.27]	Base excision repair
K03649	mug; double-stranded uracil-DNA glycosylase [EC:3.2.2.28]	Base excision repair
K03652	MPG; DNA-3-methyladenine glycosylase [EC:3.2.2.21]	Base excision repair

K03655	recG; ATP-dependent DNA helicase RecG [EC:5.6.2.4]	Homologous recombination
K03657	uvrD, pcrA; ATP-dependent DNA helicase UvrD/PcrA [EC:5.6.2.4]	Mismatch repair; Nucleotide excision repair
K03660	OGG1; N-glycosylase/DNA lyase [EC:3.2.2.- 4.2.99.18]	Base excision repair
K03701	uvrA; excinuclease ABC subunit A	Nucleotide excision repair
K03702	uvrB; excinuclease ABC subunit B	Nucleotide excision repair
K03703	uvrC; excinuclease ABC subunit C	Nucleotide excision repair
K03723	mfd; transcription-repair coupling factor (superfamily II helicase) [EC:5.6.2.4]	Nucleotide excision repair
K03763	polC; DNA polymerase III subunit alpha, Gram-positive type [EC:2.7.7.7]	DNA replication; Homologous recombination; Mismatch repair
K03862	vanA; vanillate monooxygenase [EC:1.14.13.82]	Aminobenzoate degradation
K03863	vanB; vanillate monooxygenase ferredoxin subunit	Aminobenzoate degradation
K03898	pvdA	Iron acquisition (siderophores)
K03921	FAB2, SSI2, desA1; acyl-[acyl-carrier protein] desaturase [EC:1.14.19.2 1.14.19.11 1.14.19.26]	Fatty acid biosynthesis
K03922	desA2; acyl-[acyl-carrier protein] desaturase [EC:1.14.19.2 1.14.19.-]	Fatty acid biosynthesis
K04066	priA; primosomal protein N' (replication factor Y) (superfamily II helicase) [EC:5.6.2.4]	Homologous recombination
K04067	priC; primosomal replication protein N''	Homologous recombination
K04072	adhE; acetaldehyde dehydrogenase / alcohol dehydrogenase [EC:1.2.1.10 1.1.1.1]	Chloroalkane and chloroalkene degradation; Degradation of aromatic compounds; Fatty acid degradation; Naphthalene degradation
K04073	mhpF; acetaldehyde dehydrogenase [EC:1.2.1.10]	Benzoate degradation; Degradation of aromatic compounds; Xylene degradation
K04091	ssuD	Sulfur metabolism
K04097	HPGDS; prostaglandin-H2 D-isomerase / glutathione transferase [EC:5.3.99.2 2.5.1.18]	Metabolism of xenobiotics by cytochrome P450
K04098	chqB; hydroxyquinol 1,2-dioxygenase [EC:1.13.11.37]	Benzoate degradation; Chlorocyclohexane and chlorobenzene degradation
K04099	desB, galA; gallate dioxygenase [EC:1.13.11.57]	Aminobenzoate degradation
K04100	ligA; protocatechuate 4,5-dioxygenase, alpha chain [EC:1.13.11.8]	Aminobenzoate degradation; Benzoate degradation; Polycyclic aromatic hydrocarbon degradation

K04101	ligB; protocatechuate 4,5-dioxygenase, beta chain [EC:1.13.11.8]	Aminobenzoate degradation; Benzoate degradation; Polycyclic aromatic hydrocarbon degradation
K04102	pht5; 4,5-dihydroxyphthalate decarboxylase [EC:4.1.1.55]	Polycyclic aromatic hydrocarbon degradation
K04103	ipdC	Phytohormones
K04105	hbaA; 4-hydroxybenzoate-CoA ligase [EC:6.2.1.27 6.2.1.25]	Aminobenzoate degradation; Benzoate degradation; Degradation of aromatic compounds
K04107	hcrC, hbaB; 4-hydroxybenzoyl-CoA reductase subunit gamma [EC:1.1.7.1]	Benzoate degradation; Degradation of aromatic compounds
K04108	hcrA, hbaC; 4-hydroxybenzoyl-CoA reductase subunit alpha [EC:1.1.7.1]	Benzoate degradation; Degradation of aromatic compounds
K04109	hcrB, hbaD; 4-hydroxybenzoyl-CoA reductase subunit beta [EC:1.1.7.1]; hcrB, hbaD; 4-hydroxybenzoyl-CoA reductase subunit beta [EC:1.3.7.9]	Aminobenzoate degradation; Benzoate degradation; Degradation of aromatic compounds
K04110	badA; benzoate-CoA ligase [EC:6.2.1.25]	Aminobenzoate degradation; Benzoate degradation
K04112	bcrC, badD; benzoyl-CoA reductase subunit C [EC:1.3.7.8]	Benzoate degradation; Degradation of aromatic compounds
K04113	bcrB, badE; benzoyl-CoA reductase subunit B [EC:1.3.7.8]	Benzoate degradation; Degradation of aromatic compounds
K04114	bcrA, badF; benzoyl-CoA reductase subunit A [EC:1.3.7.8]	Benzoate degradation; Degradation of aromatic compounds
K04115	bcrD, badG; benzoyl-CoA reductase subunit D [EC:1.3.7.8]	Benzoate degradation; Degradation of aromatic compounds
K04116	aliA; cyclohexanecarboxylate-CoA ligase [EC:6.2.1.-]	Benzoate degradation
K04117	aliB; cyclohexanecarboxyl-CoA dehydrogenase [EC:1.3.99.-]	Benzoate degradation
K04118	pimC; pimeloyl-CoA dehydrogenase large subunit [EC:1.3.1.62]	Benzoate degradation
K04566	lysK; lysyl-tRNA synthetase, class I [EC:6.1.1.6]	Aminoacyl-tRNA biosynthesis
K04567	KARS, lysS; lysyl-tRNA synthetase, class II [EC:6.1.1.6]	Aminoacyl-tRNA biosynthesis
K04755	fdx; ferredoxin, 2Fe-2S	Benzoate degradation
K05297	rubB, alkT; rubredoxin---NAD+ reductase [EC:1.18.1.1]	Fatty acid degradation
K05522	nei; endonuclease VIII [EC:3.2.2.- 4.2.99.18]	Base excision repair

K05549	benA-xylX; benzoate/toluate 1,2-dioxygenase subunit alpha [EC:1.14.12.10 1.14.12.-]	Benzoate degradation; Degradation of aromatic compounds; Fluorobenzoate degradation; Xylene degradation
K05550	benB-xylY; benzoate/toluate 1,2-dioxygenase subunit beta [EC:1.14.12.10 1.14.12.-]	Benzoate degradation; Degradation of aromatic compounds; Fluorobenzoate degradation; Xylene degradation
K05599	antA; anthranilate 1,2-dioxygenase (deaminating, decarboxylating) large subunit [EC:1.14.12.1]	Aminobenzoate degradation
K05600	antB; anthranilate 1,2-dioxygenase (deaminating, decarboxylating) small subunit [EC:1.14.12.1]	Aminobenzoate degradation
K05708	hcaE, hcaA1; 3-phenylpropionate/trans-cinnamate dioxygenase subunit alpha [EC:1.14.12.19]	Degradation of aromatic compounds
K05709	hcaF, hcaA2; 3-phenylpropionate/trans-cinnamate dioxygenase subunit beta [EC:1.14.12.19]	Degradation of aromatic compounds
K05710	hcaC; 3-phenylpropionate/trans-cinnamate dioxygenase ferredoxin component	Degradation of aromatic compounds
K05711	hcaB; 2,3-dihydroxy-2,3-dihydrophenylpropionate dehydrogenase [EC:1.3.1.87]	Degradation of aromatic compounds
K05712	mhpA; 3-(3-hydroxy-phenyl)propionate hydroxylase [EC:1.14.13.127]	Degradation of aromatic compounds
K05713	mhpB; 2,3-dihydroxyphenylpropionate 1,2-dioxygenase [EC:1.13.11.16]	Degradation of aromatic compounds
K05714	mhpC; 2-hydroxy-6-oxonona-2,4-dienedioate hydrolase [EC:3.7.1.14]	Degradation of aromatic compounds
K05783	benD-xylL; dihydroxycyclohexadiene carboxylate dehydrogenase [EC:1.3.1.25 1.3.1.-]	Benzoate degradation; Degradation of aromatic compounds; Fluorobenzoate degradation; Xylene degradation
K05784	benC-xylZ; benzoate/toluate 1,2-dioxygenase reductase component [EC:1.18.1.-]	Benzoate degradation; Degradation of aromatic compounds; Fluorobenzoate degradation; Xylene degradation
K05797	pchF; 4-cresol dehydrogenase (hydroxylating) flavoprotein subunit [EC:1.17.9.1]; pchF; 4-cresol dehydrogenase (hydroxylating) flavoprotein subunit [EC:1.17.99.1]	Toluene degradation
K05921	hpaG; 5-oxopent-3-ene-1,2,5-tricarboxylate decarboxylase / 2-hydroxyhepta-2,4-diene-1,7-dioate isomerase [EC:4.1.1.68 5.3.3.-]	Degradation of aromatic compounds
K05939	aas; acyl-[acyl-carrier-protein]-phospholipid O-acyltransferase / long-chain-fatty-acid--[acyl-carrier-protein] ligase [EC:2.3.1.40 6.2.1.20]	Fatty acid degradation

K06135	pqqB	Phosphorus
K06136	pqqD	Phosphorus
K06137	pqqC	Phosphorus
K06139	pqqE	Phosphorus
K06187	recR; recombination protein RecR	Homologous recombination
K06223	dam; DNA adenine methylase [EC:2.1.1.72]	Mismatch repair
K06445	fadE; acyl-CoA dehydrogenase [EC:1.3.99.-]	Fatty acid degradation
K06446	DCAA; acyl-CoA dehydrogenase [EC:1.3.99.-]	Caprolactam degradation
K06718	ectA	Stress tolerance/ osmolytes
K06720	ectC	Stress tolerance/ osmolytes
K06912	tfdA; alpha-ketoglutarate-dependent 2,4-dichlorophenoxyacetate dioxygenase [EC:1.14.11.-]	Chlorocyclohexane and chlorobenzene degradation; Degradation of aromatic compounds
K06998	phzF	Biocontrol
K07104	catE; catechol 2,3-dioxygenase [EC:1.13.11.2]	Benzoate degradation; Chlorocyclohexane and chlorobenzene degradation; Degradation of aromatic compounds; Xylene degradation
K07173	luxS	Quorum sensing
K07408	CYP1A1; cytochrome P450 family 1 subfamily A1 [EC:1.14.14.1]	Metabolism of xenobiotics by cytochrome P450
K07409	CYP1A2; cytochrome P450 family 1 subfamily A2 [EC:1.14.14.1]	Metabolism of xenobiotics by cytochrome P450
K07410	CYP1B1; cytochrome P450 family 1 subfamily B1 [EC:1.14.14.1]	Metabolism of xenobiotics by cytochrome P450
K07415	CYP2E1; cytochrome P450 family 2 subfamily E1 [EC:1.14.14.-]	Metabolism of xenobiotics by cytochrome P450
K07416	CYP2F; cytochrome P450 family 2 subfamily F [EC:1.14.14.1]	Metabolism of xenobiotics by cytochrome P450
K07420	CYP2S1; cytochrome P450 family 2 subfamily S1 [EC:1.14.14.1]	Metabolism of xenobiotics by cytochrome P450
K07456	mutS2; DNA mismatch repair protein MutS2	Mismatch repair
K07462	recJ; single-stranded-DNA-specific exonuclease [EC:3.1.-.-]	Base excision repair; Homologous recombination; Mismatch repair
K07508	ACAA2; acetyl-CoA acyltransferase 2 [EC:2.3.1.16]	Fatty acid degradation
K07511	ECHS1; enoyl-CoA hydratase [EC:4.2.1.17]	Caprolactam degradation; Fatty acid degradation

K07514	EHHADH; enoyl-CoA hydratase / 3-hydroxyacyl-CoA dehydrogenase / 3,2-trans-enoyl-CoA isomerase [EC:4.2.1.17 1.1.1.35 5.3.3.8]	Caprolactam degradation
K07515	HADHA; enoyl-CoA hydratase / long-chain 3-hydroxyacyl-CoA dehydrogenase [EC:4.2.1.17 1.1.1.211]	Caprolactam degradation
K07516	fadN; 3-hydroxyacyl-CoA dehydrogenase [EC:1.1.1.35]	Benzoate degradation; Fatty acid degradation
K07534	badK; cyclohex-1-ene-1-carboxyl-CoA hydratase [EC:4.2.1.-]	Benzoate degradation; Degradation of aromatic compounds
K07535	badH; 2-hydroxycyclohexanecarboxyl-CoA dehydrogenase [EC:1.1.1.-]	Benzoate degradation; Degradation of aromatic compounds
K07536	badI; 2-ketocyclohexanecarboxyl-CoA hydrolase [EC:3.1.2.-]	Benzoate degradation; Degradation of aromatic compounds
K07537	dch; cyclohexa-1,5-dienecarbonyl-CoA hydratase [EC:4.2.1.100]	Benzoate degradation; Degradation of aromatic compounds
K07538	had; 6-hydroxycyclohex-1-ene-1-carbonyl-CoA dehydrogenase [EC:1.1.1.368]	Benzoate degradation; Degradation of aromatic compounds
K07539	oah; 6-oxocyclohex-1-ene-carbonyl-CoA hydrolase [EC:3.7.1.21]	Benzoate degradation; Degradation of aromatic compounds
K07540	bssA; benzylsuccinate synthase alpha subunit [EC:4.1.99.11]	Degradation of aromatic compounds; Toluene degradation
K07543	bbsE; benzylsuccinate CoA-transferase BbsE subunit [EC:2.8.3.15]	Degradation of aromatic compounds; Toluene degradation
K07544	bbsF; benzylsuccinate CoA-transferase BbsF subunit [EC:2.8.3.15]	Degradation of aromatic compounds; Toluene degradation
K07545	bbsG; (R)-benzylsuccinyl-CoA dehydrogenase [EC:1.3.8.3]	Degradation of aromatic compounds; Toluene degradation
K07546	bbsH; E-phenylitaconyl-CoA hydratase [EC:4.2.1.-]; bbsH; (E)-benzylidenesuccinyl-CoA hydratase [EC:4.2.1.180]	Degradation of aromatic compounds; Toluene degradation
K07547	bbsC; (2S)-[(R)-hydroxy(phenyl)methyl]-succinyl-CoA dehydrogenase BbsC subunit [EC:1.1.1.429]	Degradation of aromatic compounds; Toluene degradation
K07548	bbsD; (2S)-[(R)-hydroxy(phenyl)methyl]-succinyl-CoA dehydrogenase BbsD subunit [EC:1.1.1.429]	Degradation of aromatic compounds; Toluene degradation
K07549	bbsA; benzoylsuccinyl-CoA thiolase BbsA subunit [EC:2.3.1.310]	Degradation of aromatic compounds; Toluene degradation
K07550	bbsB; benzoylsuccinyl-CoA thiolase BbsB subunit [EC:2.3.1.310]	Degradation of aromatic compounds; Toluene degradation

K07782	sdiA	Quorum sensing
K07823	pcaF; 3-oxoadipyl-CoA thiolase [EC:2.3.1.174]	Benzoate degradation
K07824	CYP53A1; benzoate 4-monooxygenase [EC:1.14.14.92]	Benzoate degradation; Degradation of aromatic compounds
K08295	abmG; 2-aminobenzoate-CoA ligase [EC:6.2.1.32]	Aminobenzoate degradation
K08686	cbdA; 2-halobenzoate 1,2-dioxygenase large subunit [EC:1.14.12.13]	Degradation of aromatic compounds; Fluorobenzoate degradation
K08689	bphAa, bphA1, bphA; biphenyl 2,3-dioxygenase subunit alpha [EC:1.14.12.18]	Degradation of aromatic compounds
K08690	bphB; cis-2,3-dihydrobiphenyl-2,3-diol dehydrogenase [EC:1.3.1.56]	Degradation of aromatic compounds
K09458	fabF, OXSM, CEM1; 3-oxoacyl-[acyl-carrier-protein] synthase II [EC:2.3.1.179]	Fatty acid biosynthesis
K09461	E1.14.13.40; anthraniloyl-CoA monooxygenase [EC:1.14.13.40]	Aminobenzoate degradation
K09478	ACADSB; short-chain 2-methylacyl-CoA dehydrogenase [EC:1.3.8.5]	Fatty acid degradation
K09482	gatD; glutamyl-tRNA(Gln) amidotransferase subunit D [EC:6.3.5.7]	Aminoacyl-tRNA biosynthesis
K09698	gltX; nondiscriminating glutamyl-tRNA synthetase [EC:6.1.1.24]	Aminoacyl-tRNA biosynthesis
K09759	aspC, aspS; nondiscriminating aspartyl-tRNA synthetase [EC:6.1.1.23]	Aminoacyl-tRNA biosynthesis
K10215	ethA; monooxygenase [EC:1.14.13.-]	Aminobenzoate degradation
K10216	dmpD, xylF; 2-hydroxymuconate-semialdehyde hydrolase [EC:3.7.1.9]	Benzoate degradation; Degradation of aromatic compounds; Xylene degradation
K10217	dmpC, xylG, praB; aminomuconate-semialdehyde/2-hydroxymuconate-6-semialdehyde dehydrogenase [EC:1.2.1.32 1.2.1.85]	Benzoate degradation; Degradation of aromatic compounds; Xylene degradation
K10218	ligK, galC; 4-hydroxy-4-methyl-2-oxoglutarate aldolase [EC:4.1.3.17]	Benzoate degradation
K10219	ligC; 2-hydroxy-4-carboxymuconate semialdehyde hemiacetal dehydrogenase [EC:1.1.1.312]	Aminobenzoate degradation; Benzoate degradation; Degradation of aromatic compounds
K10220	ligJ; 4-oxalmesaconate hydratase [EC:4.2.1.83]	Benzoate degradation
K10221	ligI; 2-pyrone-4,6-dicarboxylate lactonase [EC:3.1.1.57]	Aminobenzoate degradation; Benzoate degradation

K10222	bphD; 2,6-dioxo-6-phenylhexa-3-enoate hydrolase [EC:3.7.1.8]	Degradation of aromatic compounds
K10563	mutM, fpg; formamidopyrimidine-DNA glycosylase [EC:3.2.2.23 4.2.99.18]	Base excision repair
K10616	cymAa; p-cymene methyl-monooxygenase [EC:1.14.15.25]	Degradation of aromatic compounds; Xylene degradation
K10617	cymB; p-cumic alcohol dehydrogenase	Degradation of aromatic compounds; Xylene degradation
K10618	cymC; p-cumic aldehyde dehydrogenase	Degradation of aromatic compounds; Xylene degradation
K10619	cmtAb; p-cumate 2,3-dioxygenase subunit alpha [EC:1.14.12.25]	Degradation of aromatic compounds; Xylene degradation
K10620	cmtB; 2,3-dihydroxy-2,3-dihydro-p-cumate dehydrogenase [EC:1.3.1.58]	Degradation of aromatic compounds; Xylene degradation
K10621	cmtC, dhbA; 2,3-dihydroxy-p-cumate/2,3-dihydroxybenzoate 3,4-dioxygenase [EC:1.13.11.- 1.13.11.14]	Benzoate degradation; Degradation of aromatic compounds; Xylene degradation
K10622	cmtD, dhbB; HCOMODA/2-hydroxy-3-carboxy-muconic semialdehyde decarboxylase [EC:4.1.1.-]	Benzoate degradation; Degradation of aromatic compounds; Xylene degradation
K10623	cmtE; HOMODA hydrolase [EC:3.7.1.-]	Degradation of aromatic compounds; Xylene degradation
K10676	tfdB; 2,4-dichlorophenol 6-monooxygenase [EC:1.14.13.20]	Chlorocyclohexane and chlorobenzene degradation; Degradation of aromatic compounds
K10700	ebdA; ethylbenzene hydroxylase subunit alpha [EC:1.17.99.2]	Degradation of aromatic compounds
K10701	apc; acetophenone carboxylase [EC:6.4.1.8]	Degradation of aromatic compounds
K10702	todF; 2-hydroxy-6-oxohepta-2,4-dienoate hydrolase [EC:3.7.1.25]	Degradation of aromatic compounds; Xylene degradation
K10742	DNA2; DNA replication ATP-dependent helicase/nuclease Dna2 [EC:5.6.2.3 3.1.-.-]	DNA replication
K10747	LIG1; DNA ligase 1 [EC:6.5.1.1 6.5.1.6 6.5.1.7]	Base excision repair; DNA replication; Mismatch repair; Nucleotide excision repair
K10773	NTHL1, nth; endonuclease III [EC:3.2.2.- 4.2.99.18]	Base excision repair
K10780	fabL; enoyl-[acyl-carrier protein] reductase III [EC:1.3.1.104]	Fatty acid biosynthesis
K10798	PARP3_4; protein mono-ADP-ribosyltransferase 3/4 [EC:2.4.2.-]	Base excision repair

K10800	SMUG1; single-strand selective monofunctional uracil DNA glycosylase [EC:3.2.2.-]	Base excision repair
K10843	ERCC3, XPB; DNA excision repair protein ERCC-3 [EC:5.6.2.4]	Nucleotide excision repair
K10844	ERCC2, XPD; DNA excision repair protein ERCC-2 [EC:5.6.2.3]	Nucleotide excision repair
K10857	exoX; exodeoxyribonuclease X [EC:3.1.11.-]	Mismatch repair
K10979	ku; DNA end-binding protein Ku	Non-homologous end-joining
K11263	bccA, pccA, accA3; acetyl-CoA/propionyl-CoA/long-chain acyl-CoA carboxylase, biotin carboxylase, biotin carboxyl carrier protein [EC:6.4.1.2 6.4.1.3 6.4.1.- 6.3.4.14]	Fatty acid biosynthesis
K11311	antC; anthranilate 1,2-dioxygenase reductase component [EC:1.18.1.-]	Aminobenzoate degradation
K11533	fas; fatty acid synthase, bacteria type [EC:2.3.1.-]	Fatty acid biosynthesis
K11822	SULT2A; bile-salt sulfotransferase [EC:2.8.2.14]	Metabolism of xenobiotics by cytochrome P450
K11936	pgaC	Competition/colonization
K11937	pgaD	Competition/colonization
K11943	nidA; PAH dioxygenase large subunit [EC:1.13.11.-]	Degradation of aromatic compounds; Polycyclic aromatic hydrocarbon degradation
K11944	nidB; PAH dioxygenase small subunit [EC:1.13.11.-]	Degradation of aromatic compounds; Polycyclic aromatic hydrocarbon degradation
K11945	phdF; extradiol dioxygenase [EC:1.13.11.-]	Degradation of aromatic compounds; Polycyclic aromatic hydrocarbon degradation
K11946	phdG; hydratase-aldolase [EC:4.1.2.-]	Degradation of aromatic compounds; Polycyclic aromatic hydrocarbon degradation
K11947	nidD; aldehyde dehydrogenase [EC:1.2.1.-]	Degradation of aromatic compounds; Polycyclic aromatic hydrocarbon degradation
K11948	phdI; 1-hydroxy-2-naphthoate dioxygenase [EC:1.13.11.38]	Polycyclic aromatic hydrocarbon degradation
K11949	phdJ; 4-(2-carboxyphenyl)-2-oxobut-3-enoate aldolase [EC:4.1.2.34]	Polycyclic aromatic hydrocarbon degradation
K12508	fcs; trans-feruloyl-CoA synthase [EC:6.2.1.34]	Aminobenzoate degradation
K13060	luxI/lasI	Quorum sensing
K13063	phzE	Biocontrol
K13239	ECI2, PECI, ECI1; Delta3-Delta2-enoyl-CoA isomerase [EC:5.3.3.8]	Fatty acid degradation

K13299	GSTK1; glutathione S-transferase kappa 1 [EC:2.5.1.18]	Metabolism of xenobiotics by cytochrome P450
K13529	ada-alkA; AraC family transcriptional regulator, regulatory protein of adaptative response / DNA-3-methyladenine glycosylase II [EC:3.2.2.21]	Base excision repair
K13767	fadB; enoyl-CoA hydratase [EC:4.2.1.17]	Benzoate degradation; Fatty acid degradation
K13951	ADH1_7; alcohol dehydrogenase 1/7 [EC:1.1.1.1]	Metabolism of xenobiotics by cytochrome P450
K13952	ADH6; alcohol dehydrogenase 6 [EC:1.1.1.1]	Metabolism of xenobiotics by cytochrome P450
K13953	adhP; alcohol dehydrogenase, propanol-preferring [EC:1.1.1.1]	Chloroalkane and chloroalkene degradation; Degradation of aromatic compounds; Fatty acid degradation; Metabolism of xenobiotics by cytochrome P450; Naphthalene degradation
K13954	yiaY; alcohol dehydrogenase [EC:1.1.1.1]	Chloroalkane and chloroalkene degradation; Degradation of aromatic compounds; Fatty acid degradation; Naphthalene degradation
K13980	ADH4; alcohol dehydrogenase 4 [EC:1.1.1.1]	Metabolism of xenobiotics by cytochrome P450
K14028	mdh1, mxaF; methanol dehydrogenase (cytochrome c) subunit 1 [EC:1.1.2.7]	Chloroalkane and chloroalkene degradation
K14029	mdh2, mxaI; methanol dehydrogenase (cytochrome c) subunit 2 [EC:1.1.2.7]	Chloroalkane and chloroalkene degradation
K14159	rnhA-dnaQ; ribonuclease HI / DNA polymerase III subunit epsilon [EC:3.1.26.4 2.7.7.7]	DNA replication; Homologous recombination; Mismatch repair
K14164	glyQS; glycyl-tRNA synthetase [EC:6.1.1.14]	Aminoacyl-tRNA biosynthesis
K14333	DHBD; 2,3-dihydroxybenzoate decarboxylase [EC:4.1.1.46]	Aminobenzoate degradation; Benzoate degradation
K14334	CMLE; carboxy-cis,cis-muconate cyclase [EC:5.5.1.5]	Benzoate degradation
K14338	cypD_E, CYP102A, CYP505; cytochrome P450 / NADPH-cytochrome P450 reductase [EC:1.14.14.1 1.6.2.4]	Aminobenzoate degradation; Fatty acid degradation
K14417	fcbA; 4-chlorobenzoate-CoA ligase [EC:6.2.1.33]	Fluorobenzoate degradation
K14418	fcbB; 4-chlorobenzoyl-CoA dehalogenase [EC:3.8.1.7]	Fluorobenzoate degradation
K14419	caaD1; alpha-subunit of trans-3-chloroacrylic acid dehalogenase [EC:3.8.1.-]	Chloroalkane and chloroalkene degradation
K14519	aldH; NADP-dependent aldehyde dehydrogenase [EC:1.2.1.4]	Caprolactam degradation; Degradation of aromatic compounds

K14578	nahAb, nagAb, ndoA, nbzAb, dntAb; naphthalene 1,2-dioxygenase ferredoxin component	Aminobenzoate degradation; Degradation of aromatic compounds; Naphthalene degradation; Polycyclic aromatic hydrocarbon degradation
K14579	nahAc, ndoB, nbzAc, dntAc; naphthalene 1,2-dioxygenase subunit alpha [EC:1.14.12.12 1.14.12.23 1.14.12.24]	Aminobenzoate degradation; Degradation of aromatic compounds; Naphthalene degradation; Polycyclic aromatic hydrocarbon degradation
K14580	nahAd, ndoC, nbzAd, dntAd; naphthalene 1,2-dioxygenase subunit beta [EC:1.14.12.12 1.14.12.23 1.14.12.24]	Aminobenzoate degradation; Degradation of aromatic compounds; Naphthalene degradation; Polycyclic aromatic hydrocarbon degradation
K14581	nahAa, nagAa, ndoR, nbzAa, dntAa; naphthalene 1,2-dioxygenase ferredoxin reductase component [EC:1.18.1.7]	Aminobenzoate degradation; Degradation of aromatic compounds; Naphthalene degradation; Polycyclic aromatic hydrocarbon degradation
K14582	nahB, doxE; cis-1,2-dihydro-1,2-dihydroxynaphthalene/dibenzothiophene dihydrodiol dehydrogenase [EC:1.3.1.29 1.3.1.60]	Degradation of aromatic compounds; Naphthalene degradation; Polycyclic aromatic hydrocarbon degradation
K14583	nahC; 1,2-dihydroxynaphthalene dioxygenase [EC:1.13.11.56]	Degradation of aromatic compounds; Naphthalene degradation
K14584	nahD; 2-hydroxychromene-2-carboxylate isomerase [EC:5.99.1.4]	Degradation of aromatic compounds; Naphthalene degradation
K14585	nahE; trans-o-hydroxybenzylidenepyruvate hydratase-aldolase [EC:4.1.2.45]	Degradation of aromatic compounds; Naphthalene degradation
K14586	nmoAB; 2-naphthoate monooxygenase	Naphthalene degradation
K14599	dbfA1; dibenzofuran dioxygenase subunit alpha [EC:1.14.12.-]	Degradation of aromatic compounds; Polycyclic aromatic hydrocarbon degradation
K14600	dbfA2; dibenzofuran dioxygenase subunit beta [EC:1.14.12.-]	Degradation of aromatic compounds; Polycyclic aromatic hydrocarbon degradation
K14601	flnB; 1,1a-dihydroxy-1-hydro-9-fluorenone dehydrogenase	Polycyclic aromatic hydrocarbon degradation
K14602	flnD1; 2'-carboxy-2,3-dihydroxybiphenyl 1,2-dioxygenase large subunit	Polycyclic aromatic hydrocarbon degradation
K14603	flnD2; 2'-carboxy-2,3-dihydroxybiphenyl 1,2-dioxygenase small subunit and ferredoxin fusion protein	Polycyclic aromatic hydrocarbon degradation
K14604	flnE; 2-hydroxy-6-oxo-6-(2'-carboxyphenyl)-hexa-2,4-dienoate hydrolase [EC:3.7.1.-]	Polycyclic aromatic hydrocarbon degradation
K14727	pcaL; 3-oxoadipate enol-lactonase / 4-carboxymuconolactone decarboxylase [EC:3.1.1.24 4.1.1.44]	Benzoate degradation; Degradation of aromatic compounds
K14731	mlhB, chnC; epsilon-lactone hydrolase [EC:3.1.1.83]	Caprolactam degradation; Degradation of aromatic compounds

K14746	ped; (S)-1-phenylethanol dehydrogenase [EC:1.1.1.311]	Degradation of aromatic compounds
K14747	bal; benzoylacetate-CoA ligase [EC:6.2.1.-]	Degradation of aromatic compounds
K14748	etbAa; ethylbenzene dioxygenase subunit alpha [EC:1.14.12.-]	Degradation of aromatic compounds
K14749	etbAb; ethylbenzene dioxygenase subunit beta [EC:1.14.12.-]	Degradation of aromatic compounds
K14750	etbAc; ethylbenzene dioxygenase ferredoxin component	Degradation of aromatic compounds
K14751	etbC; 2,3-dihydroxyethylbenzene 1,2-dioxygenase [EC:1.13.11.-]	Degradation of aromatic compounds
K15054	mdlB; (S)-mandelate dehydrogenase [EC:1.1.99.31]	Aminobenzoate degradation
K15061	ligZ; OH-DDVA oxygenase	Aminobenzoate degradation
K15064	desA; syringate O-demethylase [EC:2.1.1.-]	Aminobenzoate degradation
K15065	desZ; 3-O-methylgallate 3,4-dioxygenase [EC:1.13.11.-]	Aminobenzoate degradation
K15066	ligM; vanillate/3-O-methylgallate O-demethylase [EC:2.1.1.341]	Aminobenzoate degradation
K15242	pcpA; 2,6-dichloro-p-hydroquinone 1,2-dioxygenase [EC:1.13.11.-]	Degradation of aromatic compounds
K15303	AKR7; aflatoxin B1 aldehyde reductase	Metabolism of xenobiotics by cytochrome P450
K15511	boxA; benzoyl-CoA 2,3-epoxidase subunit A [EC:1.14.13.208]	Benzoate degradation
K15512	boxB; benzoyl-CoA 2,3-epoxidase subunit B [EC:1.14.13.208]	Benzoate degradation
K15513	boxC; benzoyl-CoA-dihydrodiol lyase [EC:4.1.2.44]	Benzoate degradation
K15514	boxD; 3,4-dehydroadipyl-CoA semialdehyde dehydrogenase [EC:1.2.1.77]	Benzoate degradation
K15567	nmsB; naphthyl-2-methylsuccinate synthase beta subunit [EC:4.1.99.-]	Degradation of aromatic compounds; Naphthalene degradation
K15568	nmsC; naphthyl-2-methylsuccinate synthase gamma subunit [EC:4.1.99.-]	Degradation of aromatic compounds; Naphthalene degradation
K15569	bnsE; naphthyl-2-methylsuccinate CoA transferase subunit [EC:2.8.3.-]	Degradation of aromatic compounds; Naphthalene degradation
K15570	bnsF; naphthyl-2-methylsuccinate CoA transferase subunit [EC:2.8.3.-]	Degradation of aromatic compounds; Naphthalene degradation

K15571	bnsG; naphthyl-2-methylsuccinyl-CoA dehydrogenase [EC:1.3.99.-]	Degradation of aromatic compounds; Naphthalene degradation
K15572	bnsH; naphthyl-2-hydroxymethylsuccinyl-CoA hydratase [EC:4.2.1.-]	Degradation of aromatic compounds; Naphthalene degradation
K15573	bnsC; naphthyl-2-hydroxymethylsuccinyl-CoA dehydrogenase BnsC subunit [EC:1.1.-.-]	Degradation of aromatic compounds; Naphthalene degradation
K15574	bnsA; naphthyl-2-oxomethyl-succinyl-CoA thiolase subunit [EC:2.3.1.-]	Degradation of aromatic compounds; Naphthalene degradation
K15575	bnsB; naphthyl-2-oxomethyl-succinyl-CoA thiolase subunit [EC:2.3.1.-]	Degradation of aromatic compounds; Naphthalene degradation
K15659	phlD	Biocontrol
K15680	HSD11B1; corticosteroid 11-beta-dehydrogenase isozyme 1 [EC:1.1.1.146]	Metabolism of xenobiotics by cytochrome P450
K15749	dbfB; 2,2',3-trihydroxybiphenyl dioxygenase [EC:1.13.11.-]	Degradation of aromatic compounds
K15750	bphAb, bphA2, bphE; biphenyl 2,3-dioxygenase subunit beta [EC:1.14.12.18]	Degradation of aromatic compounds
K15751	carAa; carbazole 1,9a-dioxygenase [EC:1.14.12.22]	Degradation of aromatic compounds
K15752	carAc; carbazole 1,9a-dioxygenase ferredoxin component	Degradation of aromatic compounds
K15753	carAd; carbazole 1,9a-dioxygenase ferredoxin reductase component	Degradation of aromatic compounds
K15754	carBa; 2'-aminobiphenyl-2,3-diol 1,2-dioxygenase, small subunit [EC:1.13.11.-]	Degradation of aromatic compounds
K15755	carBb; 2'-aminobiphenyl-2,3-diol 1,2-dioxygenase, large subunit [EC:1.13.11.-]	Degradation of aromatic compounds
K15756	carC; 2-hydroxy-6-oxo-6-(2'-aminophenyl)hexa-2,4-dienoate hydrolase [EC:3.7.1.13]	Degradation of aromatic compounds
K15757	xylM; toluene methyl-monooxygenase [EC:1.14.15.26]	Degradation of aromatic compounds; Toluene degradation; Xylene degradation
K15758	xylA; toluene methyl-monooxygenase electron transfer component [EC:1.18.1.3]	Degradation of aromatic compounds; Toluene degradation; Xylene degradation
K15760	tmoA, tbuA1, touA; toluene monooxygenase system protein A [EC:1.14.13.236 1.14.13.-]	Degradation of aromatic compounds; Toluene degradation
K15761	tmoB, tbuU, touB; toluene monooxygenase system protein B [EC:1.14.13.236 1.14.13.-]	Degradation of aromatic compounds; Toluene degradation
K15762	tmoC, tbuB, touC; toluene monooxygenase system ferredoxin subunit	Degradation of aromatic compounds; Toluene degradation

K15763	tmoD, tbuV, touD; toluene monooxygenase system protein D [EC:1.14.13.236 1.14.13.-]	Degradation of aromatic compounds; Toluene degradation
K15764	tmoE, tbuA2, touE; toluene monooxygenase system protein E [EC:1.14.13.236 1.14.13.-]	Degradation of aromatic compounds; Toluene degradation
K15765	tmoF, tbuC, touF; toluene monooxygenase electron transfer component [EC:1.18.1.3]	Degradation of aromatic compounds; Toluene degradation
K15766	absAa; 2-aminobenzenesulfonate 2,3-dioxygenase subunit alpha [EC:1.14.12.14]	Benzoate degradation; Toluene degradation
K15767	absAb; 2-aminobenzenesulfonate 2,3-dioxygenase subunit beta [EC:1.14.12.14]	Benzoate degradation; Toluene degradation
K15768	tsaM; p-toluenesulfonate methyl-monooxygenase oxygenase component TsaM [EC:1.14.13.-]	Degradation of aromatic compounds; Toluene degradation
K15769	tsaB; p-toluenesulfonate methyl-monooxygenase reductase component TsaB [EC:1.5.1.-]	Degradation of aromatic compounds; Toluene degradation
K16049	hsaC; 3,4-dihydroxy-9,10-secoandrosta-1,3,5(10)-triene-9,17-dione 4,5-dioxygenase [EC:1.13.11.25]	Degradation of aromatic compounds
K16050	hsaD; 4,5:9,10-diseco-3-hydroxy-5,9,17-trioxoandrosta-1(10),2-diene-4-oate hydrolase [EC:3.7.1.17]	Degradation of aromatic compounds
K16157	mmoX; methane monooxygenase component A alpha chain [EC:1.14.13.25]	Degradation of aromatic compounds
K16158	mmoY; methane monooxygenase component A beta chain [EC:1.14.13.25]	Degradation of aromatic compounds
K16159	mmoZ; methane monooxygenase component A gamma chain [EC:1.14.13.25]	Degradation of aromatic compounds
K16160	mmoB; methane monooxygenase regulatory protein B	Degradation of aromatic compounds
K16161	mmoC; methane monooxygenase component C [EC:1.14.13.25]	Degradation of aromatic compounds
K16162	mmoD; methane monooxygenase component D	Degradation of aromatic compounds
K16173	acd; glutaryl-CoA dehydrogenase (non-decarboxylating) [EC:1.3.99.32]	Benzoate degradation
K16242	dmpN, poxD; phenol hydroxylase P3 protein [EC:1.14.13.-]; dmpN, poxD, tomA3; phenol/toluene 2-monooxygenase (NADH) P3/A3 [EC:1.14.13.244 1.14.13.243]	Benzoate degradation; Chlorocyclohexane and chlorobenzene degradation; Degradation of aromatic compounds; Toluene degradation
K16243	dmpL, poxC; Phenol hydroxylase P1 protein; dmpL, poxB, tomA1; phenol/toluene 2-	Benzoate degradation; Chlorocyclohexane and chlorobenzene degradation; Degradation of aromatic compounds; Toluene degradation

	monooxygenase (NADH) P1/A1 [EC:1.14.13.244 1.14.13.243]	
K16244	dmpM, poxB; phenol hydroxylase P2 protein; dmpM, poxC, tomA2; phenol/toluene 2-monooxygenase (NADH) P2/A2 [EC:1.14.13.244 1.14.13.243]	Benzoate degradation; Chlorocyclohexane and chlorobenzene degradation; Degradation of aromatic compounds; Toluene degradation
K16245	dmpO, poxE; phenol hydroxylase P4 protein; dmpO, poxE, tomA4; phenol/toluene 2-monooxygenase (NADH) P4/A4 [EC:1.14.13.244 1.14.13.243]	Benzoate degradation; Chlorocyclohexane and chlorobenzene degradation; Degradation of aromatic compounds; Toluene degradation
K16246	dmpP, poxF; phenol hydroxylase P5 protein; dmpP, poxF, tomA5; phenol/toluene 2-monooxygenase (NADH) P5/A5 [EC:1.14.13.244 1.14.13.243]	Benzoate degradation; Chlorocyclohexane and chlorobenzene degradation; Degradation of aromatic compounds; Toluene degradation
K16249	dmpK, poxA; phenol hydroxylase P0 protein; dmpK, poxA, tomA0; phenol/toluene 2-monooxygenase (NADH) P0/A0	Benzoate degradation; Chlorocyclohexane and chlorobenzene degradation; Degradation of aromatic compounds; Toluene degradation
K16268	todC2, bedC2, tcbAb; benzene/toluene/chlorobenzene dioxygenase subunit beta [EC:1.14.12.3 1.14.12.11 1.14.12.26]	Benzoate degradation; Degradation of aromatic compounds; Toluene degradation
K16269	todD, tcbB; cis-1,2-dihydrobenzene-1,2-diol/chlorobenzene dihydrodiol dehydrogenase [EC:1.3.1.19 1.3.1.119]	Benzoate degradation; Degradation of aromatic compounds; Polycyclic aromatic hydrocarbon degradation; Toluene degradation
K16270	todE; 3-methylcatechol 2,3-dioxygenase [EC:1.13.11.-]	Degradation of aromatic compounds; Xylene degradation
K16303	cmtAc; p-cumate 2,3-dioxygenase subunit beta [EC:1.14.12.25]	Degradation of aromatic compounds; Xylene degradation
K16304	cmtAd; p-cumate 2,3-dioxygenase ferredoxin component	Degradation of aromatic compounds; Xylene degradation
K16319	andAc; anthranilate 1,2-dioxygenase large subunit [EC:1.14.12.1]	Aminobenzoate degradation
K16320	andAd; anthranilate 1,2-dioxygenase small subunit [EC:1.14.12.1]	Aminobenzoate degradation
K16363	lpxC-fabZ; UDP-3-O-[3-hydroxymyristoyl] N-acetylglucosamine deacetylase / 3-hydroxyacyl-[acyl-carrier-protein] dehydratase [EC:3.5.1.108 4.2.1.59]	Fatty acid biosynthesis
K16514	galD; 4-oxalomesaconate tautomerase [EC:5.3.2.8]	Benzoate degradation
K16515	galB; 4-oxalomesaconate hydratase [EC:4.2.1.83]	Benzoate degradation
K16566	exoY	Competition/colonization

K16901	K16901; anthranilate 3-monooxygenase (FAD) / 4-hydroxyphenylacetate 3-monooxygenase [EC:1.14.14.8 1.14.14.9]	Degradation of aromatic compounds
K17048	ebdB; ethylbenzene hydroxylase subunit beta	Degradation of aromatic compounds
K17049	ebdC; ethylbenzene hydroxylase subunit gamma	Degradation of aromatic compounds
K17067	mdo; formaldehyde dismutase / methanol dehydrogenase [EC:1.2.98.1 1.1.99.37]	Chloroalkane and chloroalkene degradation
K17222	soxA	Sulfur metabolism
K17224	soxB	Sulfur metabolism
K17226	soxY	Sulfur metabolism
K17683	CYP2A6; cytochrome P450 family 2 subfamily A6 [EC:1.14.14.-]	Metabolism of xenobiotics by cytochrome P450
K17685	CYP2A13; cytochrome P450 family 2 subfamily A13 [EC:1.14.14.1]	Metabolism of xenobiotics by cytochrome P450
K17689	CYP3A4; cytochrome P450 family 3 subfamily A4 [EC:1.14.13.32 1.14.14.55 1.14.14.56 1.14.14.57 1.14.14.73 1.14.14.-]	Metabolism of xenobiotics by cytochrome P450
K17690	CYP3A5; cytochrome P450 family 3 subfamily A5 [EC:1.14.14.1]	Metabolism of xenobiotics by cytochrome P450
K17709	CYP2B6; cytochrome P450 family 2 subfamily B6 [EC:1.14.14.-]	Metabolism of xenobiotics by cytochrome P450
K17712	CYP2D6; cytochrome P450 family 2 subfamily D6 [EC:1.14.14.1]	Metabolism of xenobiotics by cytochrome P450
K17719	CYP2C9; cytochrome P450 family 2 subfamily C9 [EC:1.14.14.51 1.14.14.52 1.14.14.53 1.14.14.-]	Metabolism of xenobiotics by cytochrome P450
K17754	cpnA; cyclopentanol dehydrogenase [EC:1.1.1.163]	Caprolactam degradation; Degradation of aromatic compounds
K18067	pht4; phthalate 4,5-cis-dihydrodiol dehydrogenase [EC:1.3.1.64]	Degradation of aromatic compounds; Polycyclic aromatic hydrocarbon degradation
K18068	pht3; phthalate 4,5-dioxygenase [EC:1.14.12.7]	Degradation of aromatic compounds; Polycyclic aromatic hydrocarbon degradation
K18069	pht2; phthalate 4,5-dioxygenase reductase component [EC:1.18.1.-]	Degradation of aromatic compounds; Polycyclic aromatic hydrocarbon degradation
K18074	tphA2; terephthalate 1,2-dioxygenase oxygenase component alpha subunit [EC:1.14.12.15]	Degradation of aromatic compounds; Polycyclic aromatic hydrocarbon degradation
K18075	tphA3; terephthalate 1,2-dioxygenase oxygenase component beta subunit [EC:1.14.12.15]	Degradation of aromatic compounds; Polycyclic aromatic hydrocarbon degradation

K18076	tphB; 1,2-dihydroxy-3,5-cyclohexadiene-1,4-dicarboxylate dehydrogenase [EC:1.3.1.53]	Degradation of aromatic compounds; Polycyclic aromatic hydrocarbon degradation
K18077	tphA1; terephthalate 1,2-dioxygenase reductase component [EC:1.18.1.-]	Degradation of aromatic compounds; Polycyclic aromatic hydrocarbon degradation
K18087	bphAc, bphA3, bphF; biphenyl 2,3-dioxygenase ferredoxin component	Degradation of aromatic compounds
K18088	bphAd, bphA4, bphG; biphenyl 2,3-dioxygenase ferredoxin reductase component [EC:1.18.1.3]	Degradation of aromatic compounds
K18089	todB, tcbAc; benzene/toluene/chlorobenzene dioxygenase ferredoxin component	Benzoate degradation; Degradation of aromatic compounds; Toluene degradation
K18090	todA, tcbAd; benzene/toluene/chlorobenzene dioxygenase ferredoxin reductase component [EC:1.18.1.3 1.18.1.-]	Benzoate degradation; Degradation of aromatic compounds; Toluene degradation
K18092	etbD; 2-hydroxy-6-oxo-octa-2,4-dienoate hydrolase [EC:3.7.1.-]	Degradation of aromatic compounds
K18199	inhA; cyclohexyl-isocyanide hydratase [EC:4.2.1.103]	Caprolactam degradation
K18227	cmtAa; p-cumate 2,3-dioxygenase ferredoxin reductase component [EC:1.18.1.3]	Degradation of aromatic compounds; Xylene degradation
K18242	nagG; salicylate 5-hydroxylase large subunit [EC:1.14.13.172]	Naphthalene degradation
K18243	nagH; salicylate 5-hydroxylase small subunit [EC:1.14.13.172]	Naphthalene degradation
K18251	phtAa; phthalate 3,4-dioxygenase subunit alpha [EC:1.14.12.-]	Degradation of aromatic compounds; Polycyclic aromatic hydrocarbon degradation
K18252	phtAb; phthalate 3,4-dioxygenase subunit beta [EC:1.14.12.-]	Degradation of aromatic compounds; Polycyclic aromatic hydrocarbon degradation
K18253	phtAc; phthalate 3,4-dioxygenase ferredoxin component	Degradation of aromatic compounds; Polycyclic aromatic hydrocarbon degradation
K18254	phtAd; phthalate 3,4-dioxygenase ferredoxin reductase component [EC:1.18.1.3]	Degradation of aromatic compounds; Polycyclic aromatic hydrocarbon degradation
K18255	phtB; phthalate 3,4-cis-dihydrodiol dehydrogenase [EC:1.3.1.-]	Degradation of aromatic compounds; Polycyclic aromatic hydrocarbon degradation
K18256	phtC; 3,4-dihydroxyphthalate decarboxylase [EC:4.1.1.69]	Polycyclic aromatic hydrocarbon degradation
K18257	phdE; cis-3,4-dihydrophenanthrene-3,4-diol dehydrogenase [EC:1.3.1.49]	Degradation of aromatic compounds; Polycyclic aromatic hydrocarbon degradation
K18275	phdK; 2-formylbenzoate dehydrogenase [EC:1.2.1.78]	Polycyclic aromatic hydrocarbon degradation

K18293	cymAb; p-cymene methyl-monooxygenase electron transfer component [EC:1.18.1.3]	Degradation of aromatic compounds; Xylene degradation
K18364	bphH, xylJ, tesE; 2-oxopent-4-enoate/cis-2-oxohex-4-enoate hydratase [EC:4.2.1.80 4.2.1.132]	Benzoate degradation; Degradation of aromatic compounds; Xylene degradation
K18365	bphI, xylK, nahM, tesG; 4-hydroxy-2-oxovalerate/4-hydroxy-2-oxohexanoate aldolase [EC:4.1.3.39 4.1.3.43]	Benzoate degradation; Degradation of aromatic compounds; Xylene degradation
K18366	bphJ, xylQ, nahO, tesF; acetaldehyde/propanal dehydrogenase [EC:1.2.1.10 1.2.1.87]	Benzoate degradation; Degradation of aromatic compounds; Xylene degradation
K18383	ferB; feruloyl-CoA hydratase/lyase [EC:4.1.2.61]	Aminobenzoate degradation
K18472	accD6; acetyl-CoA/propionyl-CoA carboxylase carboxyl transferase subunit [EC:6.4.1.2 6.4.1.3 2.1.3.15]	Fatty acid biosynthesis
K18473	fabY; acetoacetyl-[acyl-carrier protein] synthase [EC:2.3.1.180]	Fatty acid biosynthesis
K18474	fabM; trans-2-decenoyl-[acyl-carrier protein] isomerase [EC:5.3.3.14]	Fatty acid biosynthesis
K18541	mdlY; mandelamide amidase [EC:3.5.1.86]	Aminobenzoate degradation
K18660	ACSF3; malonyl-CoA/methylmalonyl-CoA synthetase [EC:6.2.1.76 6.2.1.-]	Fatty acid biosynthesis
K19065	mobA; 3-hydroxybenzoate 4-monooxygenase [EC:1.14.13.23]	Benzoate degradation; Degradation of aromatic compounds; Polycyclic aromatic hydrocarbon degradation
K19066	E1.3.8.10; cyclohex-1-ene-1-carbonyl-CoA dehydrogenase [EC:1.3.8.10]	Benzoate degradation
K19067	E1.3.8.11; cyclohexane-1-carbonyl-CoA dehydrogenase [EC:1.3.8.11]	Benzoate degradation
K19290	alg8	Competition/colonization
K19291	alg44	Competition/colonization
K19515	bamB; benzoyl-CoA reductase subunit BamB [EC:1.3.-.-]	Benzoate degradation; Degradation of aromatic compounds
K19516	bamC; benzoyl-CoA reductase subunit BamC [EC:1.3.-.-]	Benzoate degradation; Degradation of aromatic compounds
K19629	tsaD1; 4-(hydroxymethyl)benzenesulfonate dehydrogenase [EC:1.1.1.257]	Toluene degradation
K19630	tsaC1; 4-formylbenzenesulfonate dehydrogenase [EC:1.2.1.62]	Toluene degradation
K19958	bnsD; naphthyl-2-hydroxymethylsuccinyl-CoA dehydrogenase BnsD subunit [EC:1.1.-.-]	Degradation of aromatic compounds; Naphthalene degradation

K19960	chnA; cyclohexanol dehydrogenase [EC:1.1.1.245]	Caprolactam degradation; Degradation of aromatic compounds
K19961	chnD; 6-hydroxyhexanoate dehydrogenase [EC:1.1.1.258]	Caprolactam degradation; Degradation of aromatic compounds
K19962	chnE; 6-oxohexanoate dehydrogenase [EC:1.2.1.63]	Caprolactam degradation
K19963	nylC; 6-aminohexanoate-oligomer endohydrolase [EC:3.5.1.117]	Caprolactam degradation
K20199	pchA; 4-hydroxybenzaldehyde dehydrogenase (NADP+) [EC:1.2.1.96]	Toluene degradation
K20200	pchC; 4-cresol dehydrogenase (hydroxylating) cytochrome subunit	Toluene degradation
K20218	hipH; 4-hydroxyisophthalate hydroxylase	Toluene degradation
K20458	hcl; 3-hydroxybenzoate/4-hydroxybenzoate---CoA ligase [EC:6.2.1.37 6.2.1.27]	Benzoate degradation
K20509	madB, oadB, gcdB, mmdB; carboxybiotin decarboxylase [EC:7.2.4.1]	Benzoate degradation
K20806	bxn; bromoxynil nitrilase [EC:3.5.5.6]	Fluorobenzoate degradation
K20807	nthB; nitrile hydratase subunit beta [EC:4.2.1.84]	Fluorobenzoate degradation
K20808	cbdB; 2-halobenzoate 1,2-dioxygenase small subunit [EC:1.14.12.13]	Degradation of aromatic compounds; Fluorobenzoate degradation
K20809	cbdC; 2-halobenzoate 1,2-dioxygenase electron transfer component [EC:1.18.1.3]	Degradation of aromatic compounds; Fluorobenzoate degradation
K20941	graF; gamma-resorcylate decarboxylase [EC:4.1.1.103]	Benzoate degradation
K20942	graA; resorcinol 4-hydroxylase (FADH2) [EC:1.14.14.27]	Benzoate degradation
K20943	E1.14.13.219; resorcinol 4-hydroxylase (NADPH) [EC:1.14.13.219]	Benzoate degradation
K20944	tsdB; resorcinol 4-hydroxylase (NADH) [EC:1.14.13.220]	Benzoate degradation
K21104	E3.1.1.101; poly(ethylene terephthalate) hydrolase [EC:3.1.1.101]	Polycyclic aromatic hydrocarbon degradation
K21105	E3.1.1.102; mono(ethylene terephthalate) hydrolase [EC:3.1.1.102]	Polycyclic aromatic hydrocarbon degradation
K21317	bmoX; butane monooxygenase alpha subunit [EC:1.14.13.230]	Degradation of aromatic compounds
K21318	bmoY; butane monooxygenase beta subunit [EC:1.14.13.230]	Degradation of aromatic compounds

K21319	bmoZ; butane monooxygenase gamma subunit [EC:1.14.13.230]	Degradation of aromatic compounds
K21320	bmoC; butane monooxygenase reductase	Degradation of aromatic compounds
K21321	bmoB; butane monooxygenase regulatory protein	Degradation of aromatic compounds
K21322	bmoD; butane monooxygenase hypothetical assembly protein	Degradation of aromatic compounds
K21759	bsdD; vanillate/4-hydroxybenzoate decarboxylase subunit D [EC:4.1.1.- 4.1.1.61]	Degradation of aromatic compounds
K22270	nagX; 3-hydroxybenzoate 6-monooxygenase [EC:1.14.13.24]	Benzoate degradation
K22553	CYP199A2; 4-methoxybenzoate monooxygenase (O-demethylating) [EC:1.14.99.15]	Benzoate degradation
K22676	bphB; cis-2,3-dihydrobiphenyl-2,3-diol/cis-2,3-dihydrodiol-DDE dehydrogenase [EC:1.3.1.-]	Degradation of aromatic compounds
K22677	bphD; 2,6-dioxo-6-phenylhexa-3-enoate/TCOA hydrolase [EC:3.7.1.-]	Degradation of aromatic compounds
K22715	dxnA1; dibenzofuran dioxygenase subunit alpha [EC:1.14.12.-]	Degradation of aromatic compounds
K22716	dxnA2; dibenzofuran dioxygenase subunit beta [EC:1.14.12.-]	Degradation of aromatic compounds
K22717	dxnB; 2-hydroxy-6-oxo-6-(2-hydroxyphenoxy)-hexa-2,4-dienoate hydrolase [EC:3.7.1.-]	Degradation of aromatic compounds
K23351	gcdC, mmdC; glutaconyl-CoA/methylmalonyl-CoA decarboxylase subunit gamma	Benzoate degradation
K23352	gcdD, mmdD; glutaconyl-CoA/methylmalonyl-CoA decarboxylase subunit delta	Benzoate degradation
K23790	GSTP; glutathione S-transferase P [EC:2.5.1.18]	Metabolism of xenobiotics by cytochrome P450
K27499	chcB2; cis-4-hydroxycyclohexanecarboxylate dehydrogenase [EC:1.1.1.438]	Benzoate degradation
K27500	chcC1; 4-oxocyclohexanecarboxylate 2-dehydrogenase [EC:1.17.3.5]	Benzoate degradation
K27540	E2.3.1.315; succinyl-CoA:cyclohexane-1-carboxylate CoA transferase [EC:2.3.1.315]	Benzoate degradation
K27672	chcAa; cyclohexane-1-carboxylate 4-trans-hydroxylase [EC:1.14.15.40]	Benzoate degradation
K27673	chcB1; trans-4-hydroxycyclohexanecarboxylate dehydrogenase [EC:1.1.1.226]	Benzoate degradation

K27674	chcC2; 4-oxocyclohex-2-ene-1-carboxylate desaturase	Benzoate degradation
K27848	E2.3.1.318; 3-oxoadipate:acetyl-CoA acetyltransferase [EC:2.3.1.318]	Benzoate degradation
K28556	pimD; pimeloyl-CoA dehydrogenase small subunit [EC:1.3.1.62]	Benzoate degradation

Table S6. Differentially abundant KEGG Orthologs (KOs) identified in rhizosphere communities

The table includes KO identifiers, gene annotations, associated metabolic pathways, the number of significant pairwise comparisons, the lowest FDR-adjusted p-value obtained from Dunn's post hoc tests, and the pairwise comparisons showing significant enrichment in the contaminated site relative to non-contaminated sites (FDR < 0.05). Fold change (FC) values indicate the magnitude of enrichment in contaminated samples; "not detected" indicates that the predicted abundance was zero at the corresponding pristine site.

KO	Gene_name	Pathways	n_sig	Best FDR	Comparisons
K16304	cmtAd; p-cumate 2,3-dioxygenase ferredoxin component	Xylene degradation; Degradation of aromatic compounds	3	0.01492	Contaminated > ASPA1 (FDR=0.0149, FC=not detected); Contaminated > ASPA2 (FDR=0.0149, FC=not detected); Contaminated > Hill (FDR=0.0149, FC=not detected)
K14028	mdh1, mxaF; methanol dehydrogenase (cytochrome c) subunit 1 [EC:1.1.2.7]	Chloroalkane and chloroalkene degradation	3	0.02692	Contaminated > ASPA1 (FDR=0.0385, FC=23); Contaminated > ASPA2 (FDR=0.0385, FC=23); Contaminated > Hill (FDR=0.0269, FC=not detected)
K14029	mdh2, mxaI; methanol dehydrogenase (cytochrome c) subunit 2 [EC:1.1.2.7]	Chloroalkane and chloroalkene degradation	3	0.02692	Contaminated > ASPA1 (FDR=0.0385, FC=23); Contaminated > ASPA2 (FDR=0.0385, FC=23); Contaminated > Hill (FDR=0.0269, FC=not detected)
K14750	etbAc; ethylbenzene dioxygenase ferredoxin component	Degradation of aromatic compounds	3	0.0274	Contaminated > ASPA1 (FDR=0.0452, FC=93.17); Contaminated > ASPA2 (FDR=0.0274, FC=not detected); Contaminated > Hill (FDR=0.0452, FC=13.98)
K18254	phtAd; phthalate 3,4-dioxygenase ferredoxin reductase component [EC:1.18.1.3]	Polycyclic aromatic hydrocarbon degradation; Degradation of aromatic compounds	3	0.02764	Contaminated > ASPA1 (FDR=0.0455, FC=685); Contaminated > ASPA2 (FDR=0.0276, FC=not detected); Contaminated > Hill (FDR=0.0455, FC=7.37)

K05599	antA; anthranilate 1,2-dioxygenase (deaminating, decarboxylating) large subunit [EC:1.14.12.1]	Aminobenzoate degradation	3	0.03692	Contaminated > ASPA1 (FDR=0.0369, FC=not detected); Contaminated > ASPA2 (FDR=0.0369, FC=not detected); Contaminated > Hill (FDR=0.0369, FC=not detected)
K07536	badI; 2-ketocyclohexanecarboxyl-CoA hydrolase [EC:3.1.2.-]	Degradation of aromatic compounds; Benzoate degradation	3	0.03722	Contaminated > ASPA1 (FDR=0.0372, FC=not detected); Contaminated > ASPA2 (FDR=0.0372, FC=not detected); Contaminated > Hill (FDR=0.0372, FC=not detected)
K15061	ligZ; OH-DDVA oxygenase	Aminobenzoate degradation	3	0.03722	Contaminated > ASPA1 (FDR=0.0372, FC=not detected); Contaminated > ASPA2 (FDR=0.0372, FC=not detected); Contaminated > Hill (FDR=0.0372, FC=not detected)
K15065	desZ; 3-O-methylgallate 3,4-dioxygenase [EC:1.13.11.-]	Aminobenzoate degradation	3	0.03722	Contaminated > ASPA1 (FDR=0.0372, FC=not detected); Contaminated > ASPA2 (FDR=0.0372, FC=not detected); Contaminated > Hill (FDR=0.0372, FC=not detected)
K11945	phdF; extradiol dioxygenase [EC:1.13.11.-]	Polycyclic aromatic hydrocarbon degradation; Degradation of aromatic compounds	2	0.02164	Contaminated > ASPA1 (FDR=0.0216, FC=not detected); Contaminated > ASPA2 (FDR=0.0216, FC=not detected)
K01580	gadB	Quorum sensing	2	0.03973	Contaminated > ASPA1 (FDR=0.0397, FC=2.48); Contaminated > ASPA2 (FDR=0.0397, FC=2.72)
K15511	boxA; benzoyl-CoA 2,3-epoxidase subunit A [EC:1.14.13.208]	Benzoate degradation	2	0.007827	Contaminated > ASPA2 (FDR=0.00783, FC=12.93); Contaminated > Hill (FDR=0.0397, FC=5.73)
K03921	FAB2, SSI2, desA1; acyl-[acyl-carrier protein] desaturase [EC:1.14.19.2 1.14.19.11 1.14.19.26]	Fatty acid biosynthesis	2	0.01124	Contaminated > ASPA2 (FDR=0.0112, FC=2.2); Contaminated > Hill (FDR=0.0397, FC=1.81)
K00055	E1.1.1.90; aryl-alcohol dehydrogenase [EC:1.1.1.90]	Xylene degradation; Toluene degradation; Degradation of aromatic compounds	2	0.01598	Contaminated > ASPA1 (FDR=0.0397, FC=3.14); Contaminated > ASPA2 (FDR=0.016, FC=5.07)
K11533	fas; fatty acid synthase, bacteria type [EC:2.3.1.-]	Fatty acid biosynthesis	2	0.01598	Contaminated > ASPA2 (FDR=0.016, FC=30.52); Contaminated > Hill (FDR=0.0216, FC=6.13)
K04117	aliB; cyclohexanecarb	Benzoate degradation	2	0.01973	Contaminated > ASPA1 (FDR=0.0316, FC=166); Contaminated > ASPA2 (FDR=0.0197, FC=not detected)

	oxyl-CoA dehydrogenase [EC:1.3.99.-]				
K00255	ACADL; long-chain-acyl-CoA dehydrogenase [EC:1.3.8.8]	Fatty acid degradation	2	0.0313	Contaminated > ASPA2 (FDR=0.0397, FC=5.64); Contaminated > Hill (FDR=0.0313, FC=4.55)
K02364	entD	Iron acquisition (siderophores)	2	0.04252	Contaminated > ASPA1 (FDR=0.0455, FC=9.17); Contaminated > ASPA2 (FDR=0.0425, FC=10.7)
K04100	ligA; protocatechuate 4,5-dioxygenase, alpha chain [EC:1.13.11.8]	Aminobenzoate degradation; Polycyclic aromatic hydrocarbon degradation; Benzoate degradation	1	0.005393	Contaminated > ASPA2 (FDR=0.00539, FC=10.17)
K04101	ligB; protocatechuate 4,5-dioxygenase, beta chain [EC:1.13.11.8]	Aminobenzoate degradation; Polycyclic aromatic hydrocarbon degradation; Benzoate degradation	1	0.006506	Contaminated > ASPA2 (FDR=0.00651, FC=10.11)
K01909	mbtM; long-chain-fatty-acid--[acyl-carrier-protein] ligase [EC:6.2.1.20]	Fatty acid degradation	1	0.007749	Contaminated > ASPA2 (FDR=0.00775, FC=82.88)
K03922	desA2; acyl-[acyl-carrier protein] desaturase [EC:1.14.19.2 1.14.19.-]	Fatty acid biosynthesis	1	0.007749	Contaminated > ASPA2 (FDR=0.00775, FC=65.59)
K10215	ethA; monooxygenase [EC:1.14.13.-]	Aminobenzoate degradation	1	0.007749	Contaminated > ASPA2 (FDR=0.00775, FC=65.59)
K05713	mhpB; 2,3-dihydroxyphenylpropionate 1,2-dioxygenase [EC:1.13.11.16]	Degradation of aromatic compounds	1	0.007827	Contaminated > ASPA2 (FDR=0.00783, FC=14.32)
K07535	badH; 2-hydroxycyclohexanecarboxyl-CoA dehydrogenase [EC:1.1.1.-]	Degradation of aromatic compounds; Benzoate degradation	1	0.008944	Contaminated > ASPA2 (FDR=0.00894, FC=not detected)
K01666	mhpE; 4-hydroxy 2-oxovalerate aldolase [EC:4.1.3.39]	Xylene degradation; Degradation of aromatic compounds;	1	0.009392	Contaminated > ASPA2 (FDR=0.00939, FC=6.17)

		Benzoate degradation			
K17224	soxB	Sulfur metabolism	1	0.009392	Contaminated > ASPA2 (FDR=0.00939, FC=7.58)
K17754	cpnA; cyclopentanol dehydrogenase [EC:1.1.1.163]	Caprolactam degradation; Degradation of aromatic compounds	1	0.01008	Contaminated > ASPA2 (FDR=0.0101, FC=272.5)
K16320	andAd; anthranilate 1,2-dioxygenase small subunit [EC:1.14.12.1]	Aminobenzoate degradation	1	0.01082	Contaminated > ASPA2 (FDR=0.0108, FC=not detected)
K16049	hsaC; 3,4-dihydroxy-9,10-secoandrosta-1,3,5(10)-triene-9,17-dione 4,5-dioxygenase [EC:1.13.11.25]	Degradation of aromatic compounds	1	0.01124	Contaminated > ASPA2 (FDR=0.0112, FC=6.36)
K00129	E1.2.1.5; aldehyde dehydrogenase (NAD(P)+) [EC:1.2.1.5]	Metabolism of xenobiotics by cytochrome P450	1	0.01318	Contaminated > ASPA2 (FDR=0.0132, FC=499.75)
K05550	benB-xylY; benzoate/toluate 1,2-dioxygenase subunit beta [EC:1.14.12.10 1.14.12.-]	Fluorobenzoate degradation; Xylene degradation; Degradation of aromatic compounds; Benzoate degradation	1	0.0133	Contaminated > ASPA2 (FDR=0.0133, FC=6.31)
K18256	phtC; 3,4-dihydroxyphthalate decarboxylase [EC:4.1.1.69]	Polycyclic aromatic hydrocarbon degradation	1	0.0133	Contaminated > ASPA2 (FDR=0.0133, FC=39.25)
K00496	alkB1_2, alkM; alkane 1-monooxygenase [EC:1.14.15.3]	Caprolactam degradation; Fatty acid degradation	1	0.01598	Contaminated > ASPA2 (FDR=0.016, FC=13.52)
K05297	rubB, alkT; rubredoxin---NAD+ reductase [EC:1.18.1.1]	Fatty acid degradation	1	0.01598	Contaminated > ASPA2 (FDR=0.016, FC=5.95)
K16050	hsaD; 4,5:9,10-diseco-3-hydroxy-5,9,17-trioxoandrosta-1(10),2-diene-4-	Degradation of aromatic compounds	1	0.01598	Contaminated > ASPA2 (FDR=0.016, FC=7.46)

	oate hydrolase [EC:3.7.1.17]				
K18383	ferB; feruloyl-CoA hydratase/lyase [EC:4.1.2.61]	Aminobenzoate degradation	1	0.01834	Contaminated > ASPA2 (FDR=0.0183, FC=not detected)
K03582	recB; exodeoxyribonuclease V beta subunit [EC:3.1.11.5]	Homologous recombination	1	0.02248	Contaminated > ASPA2 (FDR=0.0225, FC=3.01)
K03583	recC; exodeoxyribonuclease V gamma subunit [EC:3.1.11.5]	Homologous recombination	1	0.02248	Contaminated > ASPA2 (FDR=0.0225, FC=3.01)
K10219	ligC; 2-hydroxy-4-carboxymuconate semialdehyde hemiacetal dehydrogenase [EC:1.1.1.312]	Aminobenzoate degradation; Degradation of aromatic compounds; Benzoate degradation	1	0.02248	Contaminated > ASPA2 (FDR=0.0225, FC=6.12)
K04755	fdx; ferredoxin, 2Fe-2S	Benzoate degradation	1	0.02656	Contaminated > ASPA2 (FDR=0.0266, FC=3.82)
K00004	budC	Growth-Promoting volatiles	1	0.04496	Contaminated > ASPA2 (FDR=0.045, FC=3.47)
K04099	desB, galA; gallate dioxygenase [EC:1.13.11.57]	Aminobenzoate degradation	1	0.04687	Contaminated > ASPA2 (FDR=0.0469, FC=5.33)
K04116	aliA; cyclohexanecarboxylate-CoA ligase [EC:6.2.1.-]	Benzoate degradation	1	0.005168	Contaminated > ASPA2 (FDR=0.00517, FC=not detected)
K14333	DHBD; 2,3-dihydroxybenzoate decarboxylase [EC:4.1.1.46]	Aminobenzoate degradation; Benzoate degradation	1	0.005336	Contaminated > ASPA2 (FDR=0.00534, FC=52.37)
K03379	chnB; cyclohexanone monooxygenase [EC:1.14.13.22]	Caprolactam degradation; Degradation of aromatic compounds	1	0.005393	Contaminated > ASPA2 (FDR=0.00539, FC=3.77)
K04073	mhpF; acetaldehyde dehydrogenase [EC:1.2.1.10]	Xylene degradation; Degradation of aromatic compounds; Benzoate degradation	1	0.005393	Contaminated > ASPA2 (FDR=0.00539, FC=5.51)

K06446	DCAA; acyl-CoA dehydrogenase [EC:1.3.99.-]	Caprolactam degradation	1	0.005393	Contaminated > ASPA2 (FDR=0.00539, FC=14.82)
K10220	ligJ; 4-oxalmesaconate hydratase [EC:4.2.1.83]	Benzoate degradation	1	0.006439	Contaminated > ASPA2 (FDR=0.00644, FC=12.28)
K05549	benA-xylX; benzoate/toluate 1,2-dioxygenase subunit alpha [EC:1.14.12.10 1.14.12.-]	Fluorobenzoate degradation; Xylene degradation; Degradation of aromatic compounds; Benzoate degradation	1	0.009302	Contaminated > ASPA2 (FDR=0.0093, FC=9.01)
K07782	sdiA	Quorum sensing	1	0.01342	Contaminated > ASPA2 (FDR=0.0134, FC=15.6)
K17222	soxA	Sulfur metabolism	1	0.01342	Contaminated > ASPA2 (FDR=0.0134, FC=8.42)
K00108	betA	Stress tolerance/ osmolytes	1	0.01598	Contaminated > ASPA2 (FDR=0.016, FC=2.73)
K01471	nylA; 6-aminohexanoate-cyclic-dimer hydrolase [EC:3.5.2.12]	Caprolactam degradation	1	0.01598	Contaminated > ASPA2 (FDR=0.016, FC=7.12)
K01781	mdlA; mandelate racemase [EC:5.1.2.2]	Aminobenzoate degradation	1	0.01882	Contaminated > ASPA2 (FDR=0.0188, FC=8.27)
K03862	vanA; vanillate monooxygenase [EC:1.14.13.82]	Aminobenzoate degradation	1	0.01898	Contaminated > ASPA2 (FDR=0.019, FC=5.64)
K10221	ligI; 2-pyrone-4,6-dicarboxylate lactonase [EC:3.1.1.57]	Aminobenzoate degradation; Benzoate degradation	1	0.01898	Contaminated > ASPA2 (FDR=0.019, FC=11.49)
K00121	frmA, ADH5, adhC; S-(hydroxymethyl) glutathione dehydrogenase / alcohol dehydrogenase [EC:1.1.1.284 1.1.1.1]	Glycolysis / Gluconeogenesis; Pyruvate metabolism; Chloroalkane and chloroalkene degradation; Naphthalene degradation; Metabolism of xenobiotics by cytochrome P450; Degradation of aromatic compounds; Fatty acid degradation	1	0.01898	Contaminated > ASPA2 (FDR=0.019, FC=2.56)

K02361	entC	Iron acquisition (siderophores)	1	0.02248	Contaminated > ASPA2 (FDR=0.0225, FC=13.36)
K01075	E3.1.2.23; 4-hydroxybenzoyl-CoA thioesterase [EC:3.1.2.23]	Benzoate degradation	1	0.02248	Contaminated > ASPA2 (FDR=0.0225, FC=3.43)
K04110	badA; benzoate-CoA ligase [EC:6.2.1.25]	Aminobenzoate degradation; Benzoate degradation	1	0.02248	Contaminated > ASPA2 (FDR=0.0225, FC=10.29)
K15064	desA; syringate O-demethylase [EC:2.1.1.-]	Aminobenzoate degradation	1	0.02572	Contaminated > ASPA2 (FDR=0.0257, FC=not detected)
K00249	ACADM, acd; acyl-CoA dehydrogenase [EC:1.3.8.7]	Fatty acid degradation	1	0.02656	Contaminated > ASPA2 (FDR=0.0266, FC=2.2)
K00836	ectB	Stress tolerance/ osmolytes	1	0.02656	Contaminated > ASPA2 (FDR=0.0266, FC=6.95)
K16514	galD; 4-oxalomesaconate tautomerase [EC:5.3.2.8]	Benzoate degradation	1	0.02656	Contaminated > ASPA2 (FDR=0.0266, FC=71.86)
K18242	nagG; salicylate 5-hydroxylase large subunit [EC:1.14.13.172]	Naphthalene degradation	1	0.02656	Contaminated > ASPA2 (FDR=0.0266, FC=19.5)
K18243	nagH; salicylate 5-hydroxylase small subunit [EC:1.14.13.172]	Naphthalene degradation	1	0.02656	Contaminated > ASPA2 (FDR=0.0266, FC=19.5)
K16319	andAc; anthranilate 1,2-dioxygenase large subunit [EC:1.14.12.1]	Aminobenzoate degradation	1	0.0269	Contaminated > ASPA2 (FDR=0.0269, FC=not detected)
K14419	caaD1; alpha-subunit of trans-3-chloroacrylic acid dehalogenase [EC:3.8.1.-]	Chloroalkane and chloroalkene degradation	1	0.02871	Contaminated > ASPA2 (FDR=0.0287, FC=not detected)
K18092	etbD; 2-hydroxy-6-oxo-octa-2,4-dienoate hydrolase [EC:3.7.1.-]	Degradation of aromatic compounds	1	0.03106	Contaminated > ASPA2 (FDR=0.0311, FC=185.5)
K01561	dehH; haloacetate dehalogenase [EC:3.8.1.3]	Chlorocyclohexane and chlorobenzene degradation; Chloroalkane and chloroalkene degradation	1	0.0313	Contaminated > ASPA2 (FDR=0.0313, FC=4.7)

K01576	mdlC; benzoylformate decarboxylase [EC:4.1.1.7]	Aminobenzoate degradation	1	0.0313	Contaminated > ASPA2 (FDR=0.0313, FC=3.63)
K04098	chqB; hydroxyquinol 1,2-dioxygenase [EC:1.13.11.37]	Chlorocyclohexane and chlorobenzene degradation; Benzoate degradation	1	0.0313	Contaminated > ASPA2 (FDR=0.0313, FC=9.51)
K14578	nahAb, nagAb, ndoA, nbzAb, dntAb; naphthalene 1,2-dioxygenase ferredoxin component	Naphthalene degradation; Aminobenzoate degradation; Polycyclic aromatic hydrocarbon degradation; Degradation of aromatic compounds	1	0.0313	Contaminated > ASPA2 (FDR=0.0313, FC=17.94)
K14581	nahAa, nagAa, ndoR, nbzAa, dntAa; naphthalene 1,2-dioxygenase ferredoxin reductase component [EC:1.18.1.7]	Naphthalene degradation; Aminobenzoate degradation; Polycyclic aromatic hydrocarbon degradation; Degradation of aromatic compounds	1	0.0313	Contaminated > ASPA2 (FDR=0.0313, FC=17.94)
K10676	tfdB; 2,4-dichlorophenol 6-monooxygenase [EC:1.14.13.20]	Chlorocyclohexane and chlorobenzene degradation; Degradation of aromatic compounds	1	0.03135	Contaminated > ASPA2 (FDR=0.0313, FC=1625.04)
K08295	abmG; 2-aminobenzoate-CoA ligase [EC:6.2.1.32]	Aminobenzoate degradation	1	0.03424	Contaminated > ASPA2 (FDR=0.0342, FC=11.38)
K01782	fadJ; 3-hydroxyacyl-CoA dehydrogenase / enoyl-CoA hydratase / 3-hydroxybutyryl-CoA epimerase [EC:1.1.1.35 4.2.1.17 5.1.2.3]	Benzoate degradation; Caprolactam degradation; Fatty acid degradation	1	0.03679	Contaminated > ASPA2 (FDR=0.0368, FC=2.43)
K05712	mhpA; 3-(3-hydroxy-phenyl)propiona	Degradation of aromatic compounds	1	0.03679	Contaminated > ASPA2 (FDR=0.0368, FC=3.68)

	te hydroxylase [EC:1.14.13.127]				
K03464	catC; muconolactone D-isomerase [EC:5.3.3.4]	Degradation of aromatic compounds; Benzoate degradation	1	0.03679	Contaminated > ASPA2 (FDR=0.0368, FC=9.72)
K06718	ectA	Stress tolerance/ osmolytes	1	0.03679	Contaminated > ASPA2 (FDR=0.0368, FC=5.51)
K18472	accD6; acetyl-CoA/propionyl-CoA carboxylase carboxyl transferase subunit [EC:6.4.1.2 6.4.1.3 2.1.3.15]	Fatty acid biosynthesis	1	0.03679	Contaminated > ASPA2 (FDR=0.0368, FC=1.87)
K14727	pcaL; 3-oxoadipate enol-lactonase / 4-carboxymuconolactone decarboxylase [EC:3.1.1.24 4.1.1.44]	Degradation of aromatic compounds; Benzoate degradation	1	0.04113	Contaminated > ASPA2 (FDR=0.0411, FC=4.45)
K01563	dhaA; haloalkane dehalogenase [EC:3.8.1.5]	Chlorocyclohexane and chlorobenzene degradation; Chloroalkane and chloroalkene degradation	1	0.04314	Contaminated > ASPA1 (FDR=0.0431, FC=2.06)
K17226	soxY	Sulfur metabolism	1	0.04314	Contaminated > ASPA2 (FDR=0.0431, FC=2.63)

Abbreviations: FDR, false discovery rate; FC, fold change; KO, KEGG Ortholog. Of the 171 KOs differing among rhizosphere communities, the table lists the 103 with pairwise comparisons against the contaminated site.

Table S7. KEGG Orthologs showing differences among endosphere communities (Kruskal–Wallis test, uncorrected p < 0.05).

KO	Gene	Functional category	Main KEGG pathway(s)	Kruskal-Wallis p-value
K00129	aldehyde dehydrogenase (NAD(P)+)	Aromatic compound degradation	Aldehyde metabolism	0.0174
K00390	cysH	Sulfur metabolism	Sulfur metabolism	0.0481
K00626	atoB (ACAT)	Fatty acid and central metabolism	Benzoate degradation; Fatty acid metabolism; Pyruvate metabolism	0.0483
K00960	rpo13	Transcription	DNA-directed RNA polymerase	0.0390
K01007	ppsA	Central carbon metabolism	Glycolysis/Gluconeogenesis; Pyruvate metabolism	0.0327
K01399	lasB	Extracellular proteolysis	Protease activity	0.0162
K01505	acdS	Plant growth promotion	ACC deaminase	0.0453
K05599	antA	Aromatic compound degradation	Anthranilate degradation	0.0188
K05921	hpaG	Aromatic compound degradation	4-Hydroxyphenylacetate degradation	0.0231
K07535	badH	Aromatic compound degradation	Cyclohexanecarboxylate degradation	0.0167
K10622	cmtD (dhbB)	Aromatic compound degradation	p-Cumate degradation	0.0068
K10676	tfdB	Xenobiotic degradation	Chlorophenol degradation	0.0188
K10780	fabL	Fatty acid metabolism	Fatty acid biosynthesis	0.0143
K13063	phzE	Secondary metabolism	Phenazine biosynthesis	0.0275
K13767	fadB	Fatty acid metabolism	β-oxidation	0.0440
K13953	adhP	Alcohol metabolism	Alcohol dehydrogenase	0.0281
K14750	etbAc	Aromatic compound degradation	Ethylbenzene degradation	0.0049
K16304	cmtAd	Aromatic compound degradation	p-Cumate degradation	0.0049
K18067	pht4	Aromatic compound degradation	Phthalate degradation	0.0068
K18068	pht3	Aromatic compound degradation	Phthalate degradation	0.0068
K18069	pht2	Aromatic compound degradation	Phthalate degradation	0.0068
K18364	bphH / xylJ / tesE	Aromatic compound degradation	Biphenyl degradation	0.0166

Of the 24 KOs differing among endosphere communities, the table lists the 22 with pairwise comparisons against the contaminated site.

Figure S1. Taxonomic contributors to selected plant growth-promoting (PGPR) functions predicted by PICRUSt2 in rhizosphere communities. Stacked bar plots show the mean genus-level contribution to the predicted abundance of significant PGPR-related KOs across sampling sites (ASPA1, ASPA2, Hill and Contaminated).

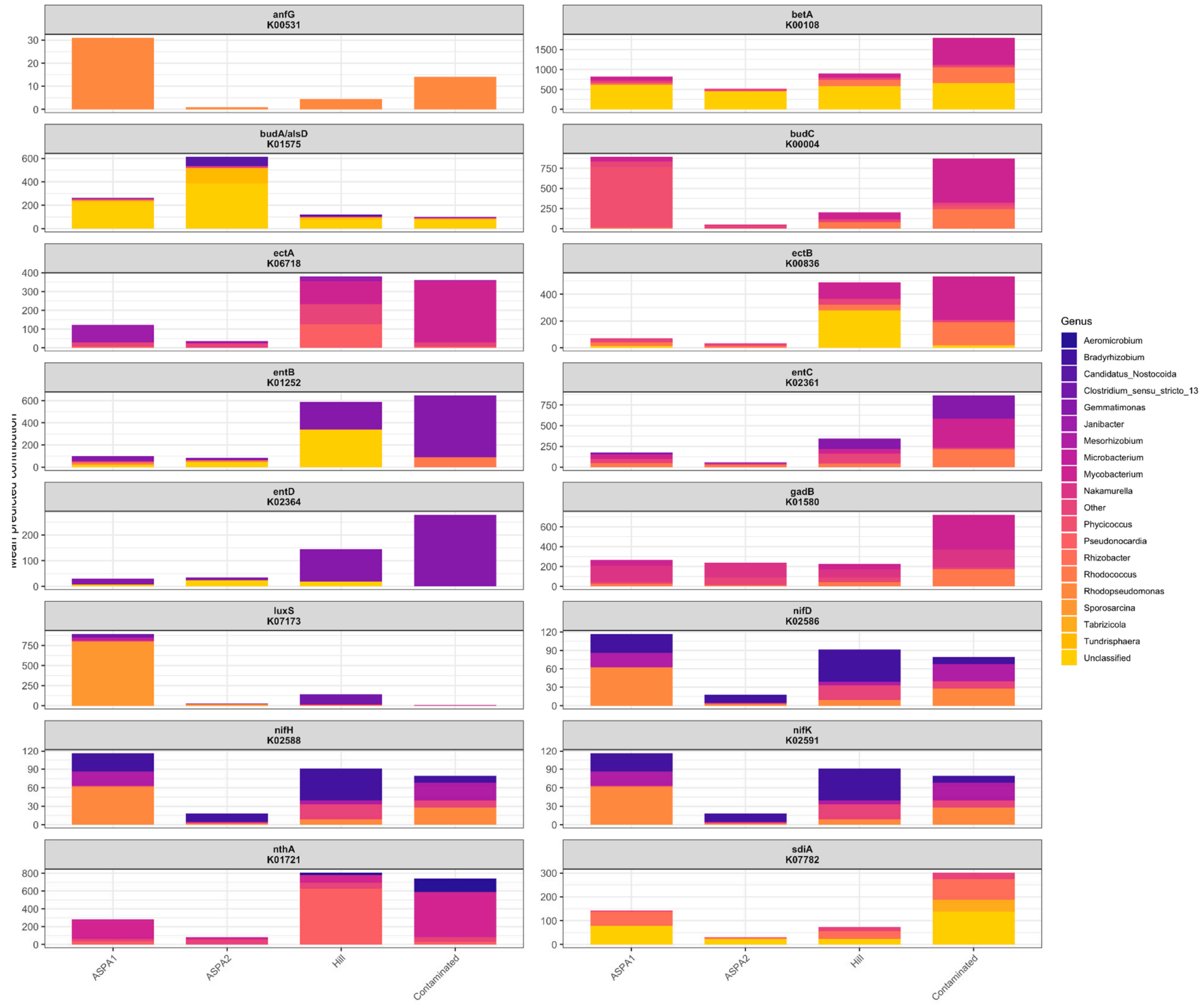
anfG
K00531
betA
K00108
budA/alsD
K01575
budC
K00004
ectA
K06718
ectB
K00836
entB
K01252
entC
K02361
entD
K02364
gadB
K01580
luxS
K07173
nifD
K02586
nifH
K02588
nifK
K02591
nthA
K01721
sdiA
K07782
ASPA1
ASPA2
Hill
Contaminated
Genus
Aeromicrobium
Bradyrhizobium
Candidatus_Nostocoida
Clostridium_sensu_stricto_13
Gemmatimonas
Janibacter
Mesorhizobium
Microbacterium
Mycobacterium
Nakamurella
Other
Phycicoccus
Pseudonocardia
Rhizobacter
Rhodococcus
Rhodopseudomonas
Sporosarcina
Tabrizicola
Tundrisphaera
Unclassified
